# Boronic acids facilitate rapid oxime condensations at neutral pH†
†Dedicated to Andreas Pfaltz on the occasion of his retirement.
‡
‡Electronic supplementary information (ESI) available: Detailed kinetic data, HPLC assays, and characterization data for all new compounds are provided. See DOI: 10.1039/c5sc00921a



**DOI:** 10.1039/c5sc00921a

**Published:** 2015-04-13

**Authors:** Pascal Schmidt, Cedric Stress, Dennis Gillingham

**Affiliations:** a Department of Chemistry , University of Basel , St. Johanns-Ring 19 , CH-4056 , Basel , Switzerland . Email: dennis.gillingham@unibas.ch

## Abstract

We report here the discovery and development of boron-assisted oxime formation as a powerful connective reaction for chemical biology. Oximes proximal to boronic acids form in neutral aqueous buffer with rate constants of more than 10^4^ M^–1^ s^–1^, the largest to date for any oxime condensation. Boron's dynamic coordination chemistry confers an adaptability that seems to aid a number of elementary steps in the oxime condensation. In addition to applications in bioconjugation, the emerging importance of boronic acids in chemical biology as carbohydrate receptors or peroxide probes, and the growing list of drugs and drug candidates containing boronic acids suggest many potential applications.

## Abstract 2

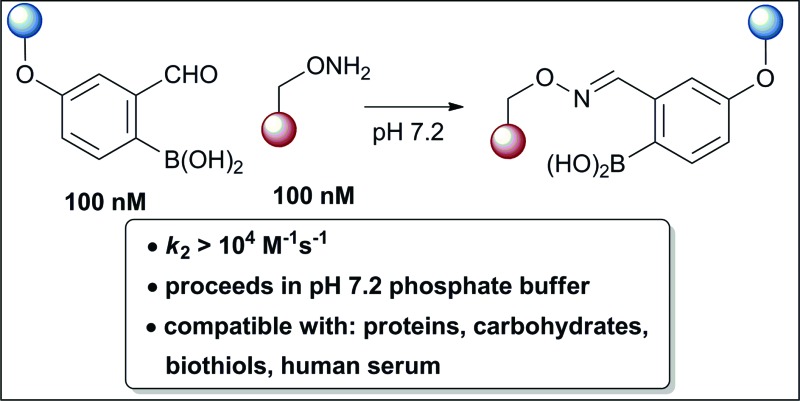
We report here the discovery and development of boron-assisted oxime formation as a powerful connective reaction for chemical biology.

## Introduction

Bioconjugation reactions create multifunctional molecules from monofunctional components, a molecular plug-and-play that is invaluable in modern biological research.^1^ Still underdeveloped, however, are biocompatible (neutral pH, tolerant to biothiols) coupling reactions that proceed fast enough to allow substrate ratios near unity at low concentrations (*i.e.* nM to µM regime).^2^ Certainly the fastest biocompatible reaction is the tetrazine inverse electron demand Diels–Alder with strained olefins,^3,4^ a recent example of which has achieved an astonishingly high rate constant.^5^ Despite the availability of faster reactions the oxime condensation remains a workhorse in chemical biology due to its simplicity and robustness;^6,7^ but the need for added catalysts or low pH to achieve acceptable rates is a distinct disadvantage.

**Fig. 1:**
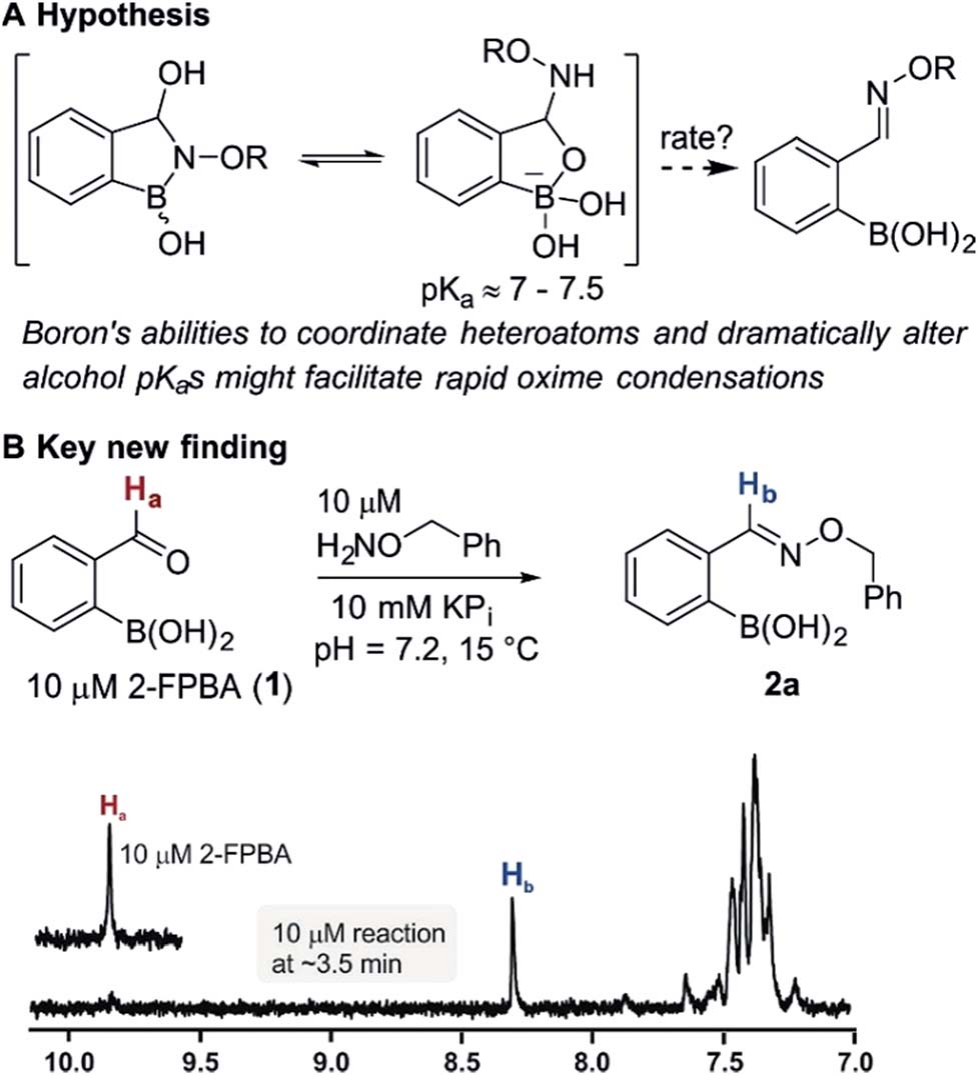
(A) The unique abilities of boron led to the hypothesis that proximal boron substituents would have an impact on oxime condensations; (B) preliminary observations confirming the potential of boron to accelerate oxime formation.

Recent works by us^8^ and others^9,10^ have shown that proximal functional groups can play a decisive role in oxime condensations and related reactions.^11^ Based on these insights, we hypothesized that the Lewis acidity of boron^12^ coupled with its ability to modulate alcohol p*K*_a_s through coordination (panel a, Fig. 1),^13,14^ might facilitate rapid oxime formation (panel b, Fig. 1). Moreover, boron is a chemically versatile element and its presence in oxime products would open the door to many applications in chemical biology. For example boron compounds have found uses in medicinal chemistry,^15,16^ cross-coupling reactions^17,18^ carbohydrate detection,^19,20^ promoting endocytosis,^21^ and reactivity-based peroxide sensing.^22,23^ Boron's rich coordination chemistry is pivotal for many of its applications. For example the N → B dative interaction has been proposed to stabilize otherwise labile Schiff bases, and these iminoboronates have been used in supramolecular chemistry,^24,25^ enantiomer analysis by NMR,^26,27^ and protein labelling.^28,29^

We selected the 2-formylphenylboronic acid (2-FPBA, **1**) scaffold as a model system to explore boron's influence on oxime condensations (Fig. 1). 2-FPBA has previously been shown to modify lysine residues or N-termini in proteins in a process controlled by rapid equilibria.^29^ Little is known, however, about the analogous condensation with α-effect nucleophiles. We have found two cases of alkoxyiminoboronates (AIBs),^27,30^ two cases of *O*-alkylAIBs^31,32^ and two cases of the related hydrazinoiminoboronates;^30,33^ but in each of those studies the coupling itself was not of central importance and therefore not examined in detail. Herein we describe that *O*-alkylAIBs form with remarkable speed and selectivity in neutral aqueous buffer. The reaction is unaffected by proteins, carbohydrates, biothiols, and human serum, and has an equilibrium constant of >10^8^ M^–1^.

## Results and discussion

As shown in panel b of Fig. 1, when 2-FPBA was reacted with *O*-benzylhydroxylamine in a 1 : 1 ratio at neutral pH at 10 µM the reaction was nearly complete after the first proton NMR measurement (>90% conversion at ∼3.5 minutes), or HPLC injection (∼1 minute, see entry 3, Table 1). These observations imply a rate upwards of 10^3^ M^–1^ s^–1^ – several orders of magnitude faster than normal oxime condensations at neutral pH,^34^ prompting us to explore the importance of boronic acid positioning on reaction efficacy. Comparing entries 1–6 in Table 1 makes it clear that the 2-FPBA motif is crucial for an efficient reaction: the corresponding *meta*- and *para*-substituted FPBAs behaved similar to benzaldehyde, delivering nearly undetectable levels of oxime after 90 minutes (*cf.* entry 1 with 5 and 6). More complex hydroxylamines, such as the pentapeptide shown in entry 4, were also excellent substrates, delivering complete conversion after the first injection. Condensations with ketones were slower than with 2-FPBA (entry 7), but still faster than normal ketoxime condensations. Pinacol boronate esters also gave complete conversion after one minute (compares entries 2 & 8) but the hydrolysed boronic acid **2a** was the main product with only 10% of pinacol ester oxime **2f** observed; after ninety minutes only **2a** was present. The ketoximine pinacol product (entry 9), on the other hand, was more stable and comprised 97% of the product after the first injection (the remainder being **2e**); after thirty minutes **2g** had also completely hydrolysed to **2e**. The ability to use pinacol esters is valuable since these are readily obtained from Pd-catalysed routes to boronic esters, and their deprotections in organic solvents are often tricky.

**Table 1:** Probing the importance of boron positioning and substitution on oxime condensations

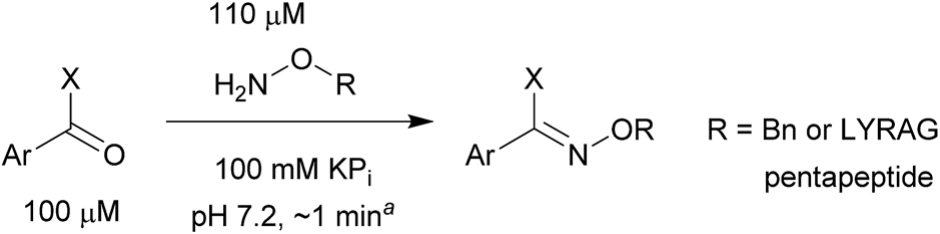					
Entry	Ar	X	Product	Conc (µM)	Conv^*b*^ (%)
1^*c*^	Ph	H	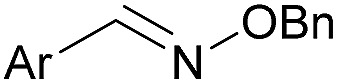	100	<5
2	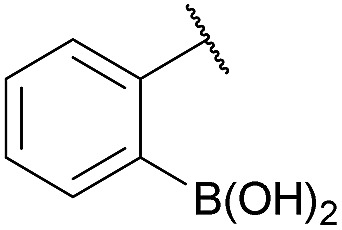	H	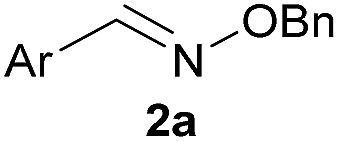	100	>98
3	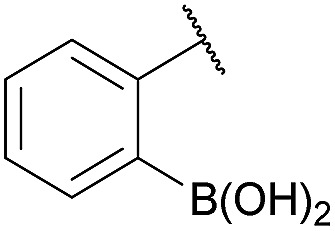	H	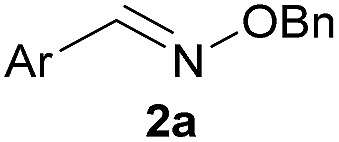	10	>98
4	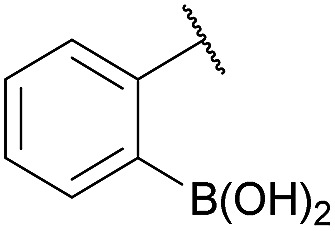	H	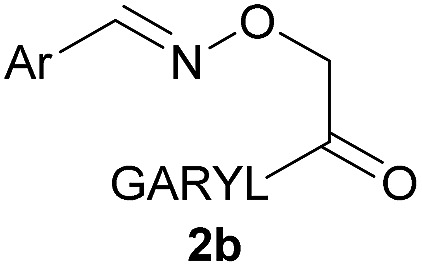	100	>98
5^*c*^	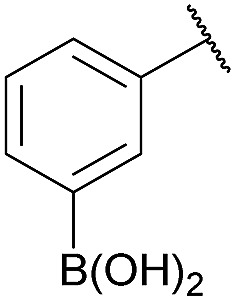	H	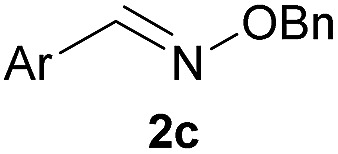	100	<5
6^*c*^	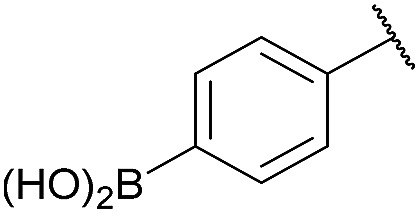	H	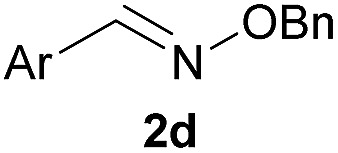	100	<5
7	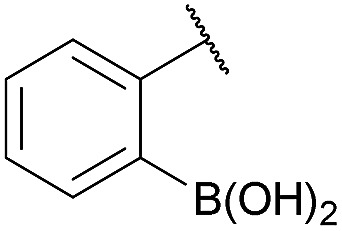	Me	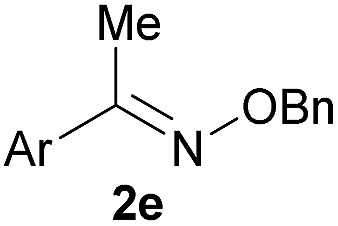	100	94
8	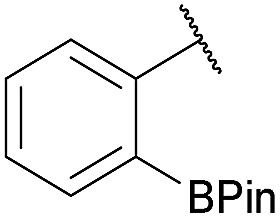	H	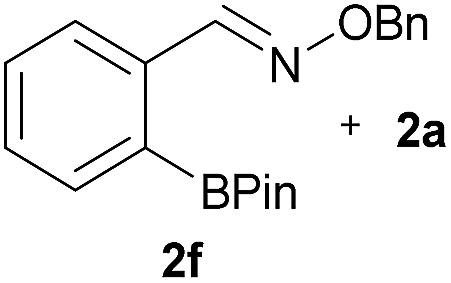	100	>98^*d*^
9	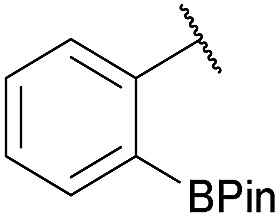	Me	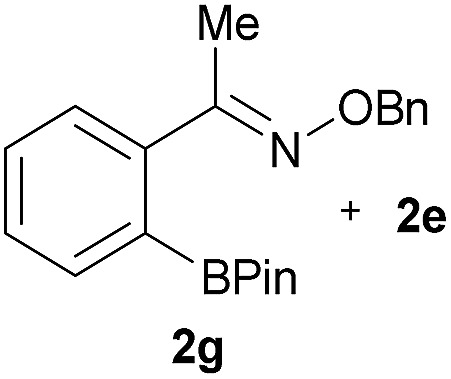	100	>98

^*a*^Time is approximate since samples are injected directly after mixing.

^*b*^Determined by reverse phase HPLC analysis under neutral conditions.

^*c*^Injections at 90 minutes still show <5% conversion.

^*d*^At the first injection approximately 10% of the pinacol ester oxime is observed, but only the hydrolysed product is detected at 90 minutes. KP_i_ = potassium phosphate buffer.

**Fig. 2:**
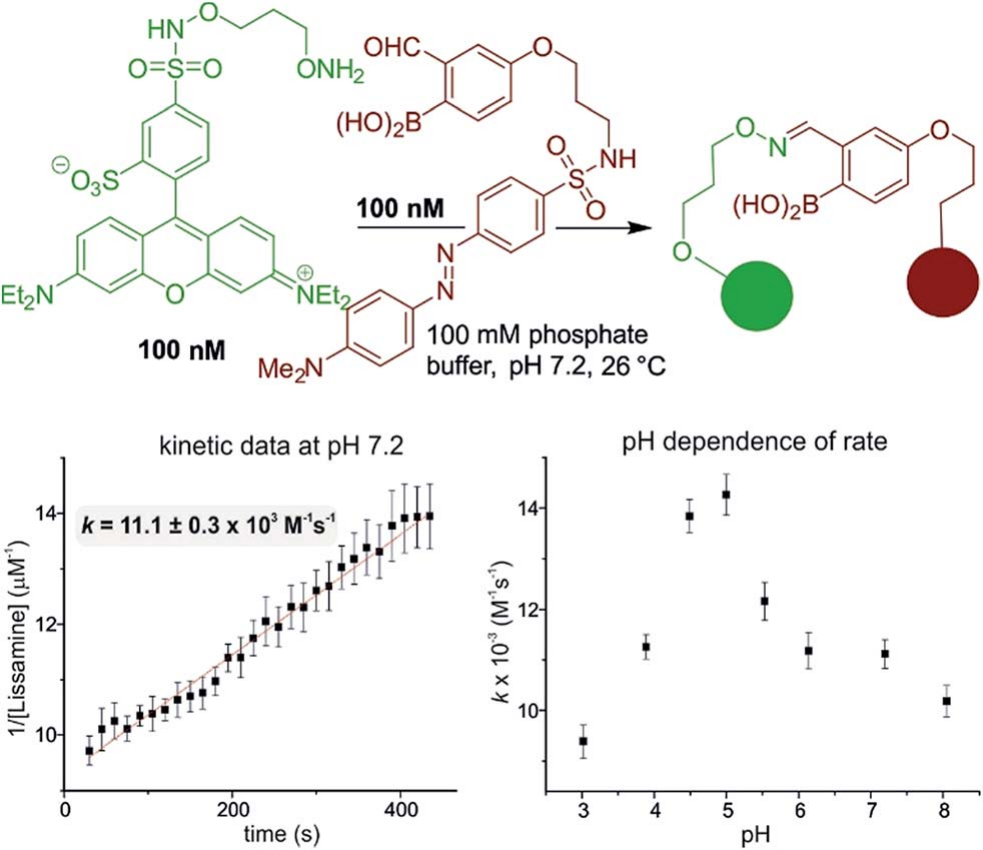
Fluorescence quenching assay to determine rate constants and pH dependence. All measurements were done in triplicate and are corrected by subtracting a control measurement where everything was identical except that the dabcyl moiety lacked a 2-FPBA function.

The reaction is fast and displays second order kinetics. We established the rate in three independent ways: HPLC, NMR, and fluorescence quenching. While the time resolution and sensitivity of HPLC and NMR could provide only a lower bound for the rate constant, fluorescence quenching gave a more quantitative assessment. A hydroxylamine bearing a lissamine fluorophore and a 2-FPBA connected to a quencher were prepared (see Fig. 2 for the structures and the ESI pages S10–11‡ for synthetic details). Scouting experiments suggested that monitoring at 100 nM would be required to obtain sufficient data at early reaction conversion (runs at 500 nM show second order behavior only in the first 100 seconds, see page S26 in the ESI‡ for details). Triplicate rate measurements at 100 nM showed excellent linearity in inverse concentration plots (see Fig. 2) and gave a rate constant of ≈11 000 M^–1^ s^–1^ – several orders of magnitude faster than the fastest neutral pH oxime condensations.^34^ Although fluorescence quenching is an indirect measurement technique, HPLC injections of aliquots from the assay mixture showed only product and starting materials (indicating a clean reaction) and gave conversions qualitatively in agreement with the fluorescence measurements. In addition, the fact that 10 and 1 µM reactions monitored by ^1^H NMR showed >90% conversion at the earliest possible measurements (see Fig. 1, and the ESI Scheme S1‡ for 1 µM case) provides independent verification of a rate constant >10^4^ M^–1^ s^–1^ since the first half-life would have to be less than 90 seconds. The general pH dependence, with a maximum in the range of 4.5–5, is consistent with normal oxime formation^35^ except there is a distortion in the sigmoidal shape at higher pH (*i.e.* 7.2 & 8). The asymmetry in the pH dependence curve is inverted in comparison to a typical oxime condensation (acetone with hydroxylamine),^35^ which shows a more pronounced drop-off in rate at higher pH than at lower pH. Based on the build-up of a tetrahedral intermediate at higher pH, Jencks attributed this pH dependence to the need for a proton in the dehydration step.^35^ Although we have thus far not been able to detect intermediates, we speculate that the near-neutral p*K*_a_ of amine-substituted aqueous boronates^14,36^ provides the ideal environment for acceleration of the normally rate-limiting dehydration.

**Fig. 3:**
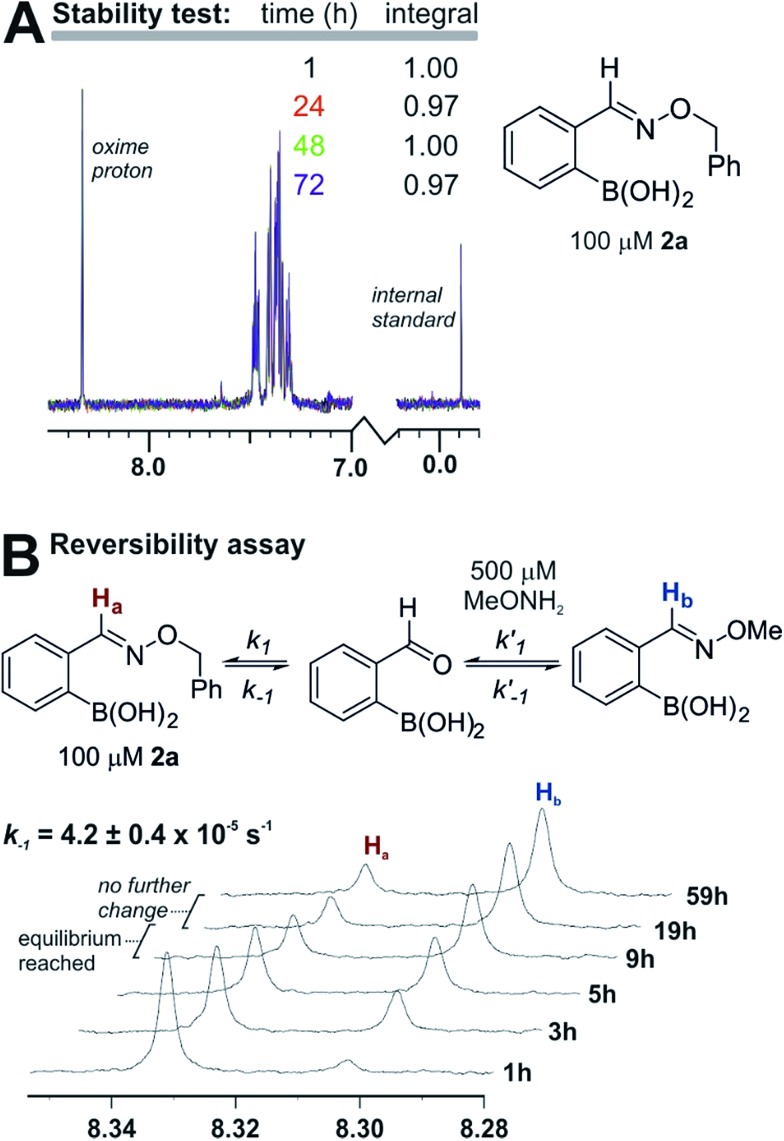
(A) Product **2a** shows no detectable decomposition after three days in 10 mM phosphate buffer at pH 7.2. (B) Product **2a** exchanges with *O*-methylhydroxylamine, reaching equilibrium over several hours in 10 mM phosphate buffer at pH 7.2.

The products are stable and undergo slow equilibration at neutral pH. Proton NMR analysis of compound **2a** (from entry 2 in Table 1) in pH 7.2 phosphate buffer led to little change in concentration (<5%) over the course of three days (see panel a in Fig. 3). We also tested whether **2a** was in equilibrium with the starting materials by adding to the NMR sample a five-fold excess of *O*-methylhydroxylamine. As shown in panel b of Fig. 3, *O*-methylhydroxylamine leads to a new oxime product, with equilibrium being established after 10–15 hours at 100 µM in pH 7.2 phosphate buffer. Although the equilibrium constant is too large to allow a static equilibrium measurement, *k*_–1_ can be calculated directly from the exchange rate between oximes (4.2 ± 0.4 × 10^–5^ s^–1^). From this value and the *k*_1_ value obtained from fluorescence quenching (Fig. 2) an equilibrium constant of 2.6 ± 0.3 × 10^8^ M^–1^ can be estimated. The reversibility assay shown in Fig. 3 would not be able to distinguish between hydrolysis and direct *O*-methylhydroxylamine attack; therefore we also ran the experiment by adding a different boronic acid to **2a** instead of *O*-methylhydroxylamine: the exchange kinetics are the same within experimental error, corroborating the hydrolysis-based mechanism (see the ESI pages S36 and S37‡ for details).

For practical applications it is important for a bioconjugation to proceed in complex environments. We therefore explored the efficacy of the boron-assisted oxime ligation in the presence of common interfering additives (sugars, biothiols, proteins, human serum). The additives led to no detectable reduction in reaction efficiency (see Table 2) with the exception of human serum. Interestingly in human serum the boronic acid in **2a** partially oxidized to a phenol, leading to an apparent loss of material: after the first injection 80% of **2a** was present while after 18 h only 20% remained (see the ESI Fig. S13‡ for details). Even in the oxidized product, however, the oxime was still intact and if both components are added together the mass balance is nearly complete. Furthermore, lysozyme, which has previously been shown to react with 2-FPBA,^29^ showed no modification according to LC-MS, indicating that the hydroxylamine entirely outcompetes nucleophilic amino acid functionalities for 2-FPBA (see Fig. S12 in the ESI‡).

**Table 2:** Tolerance of the condensation to biological interfering agents

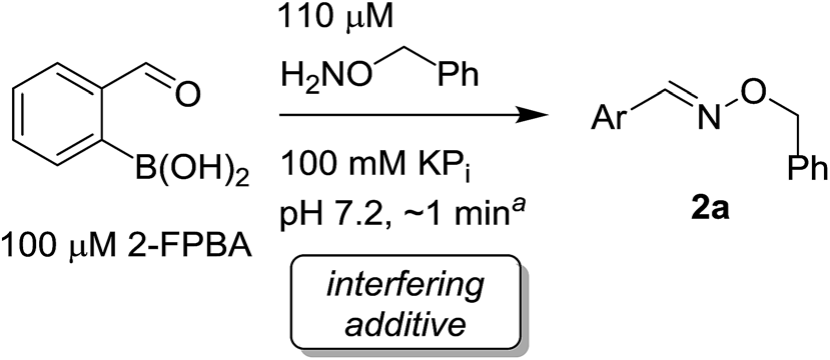			
Entry	Additive (conc)	Normalized **2a** integral^*b*^ (%)	Significance
1	None	100	—
2	Glutathione (5 mM)	98	Biothiols do not interfere
3	Sucrose (100 µM & 5 mM)	106/92^*c*^	Boron chelators do not interfere
4	Lysozyme (100 µM)	105	Amino acid side-chains cannot compete with *O*-alkylhydroxylamine for 2-FPBA
5	Human serum (20% v/v)	80^*d*^	Reaction is compatible with complex media

^*a*^Time is approximate since samples are injected directly after mixing.

^*b*^Determined by reverse phase HPLC analysis under neutral conditions.

^*c*^This reaction was also performed by pre-mixing the boronic acid with the sucrose, with no measurable change in conjugation efficiency.

^*d*^Human serum leads to oxidation of the boronic acid to a phenol by a Baeyer–Villiger type reaction. The 80% number represents only the measurement of **2a**, when the phenol is included nearly complete mass balance is observed. KP_i_ = potassium phosphate buffer.

## Conclusions

A great advantage of the present method over many coupling reactions is the simplicity and ready availability of the starting materials. There are commercial libraries of phenylboronic acid and boronic ester compounds, many of which contain an aldehyde or can be trivially elaborated to incorporate one. Furthermore, the widespread use of oxime conjugations for connective processes at high concentration means that a variety of *O*-alkylhydroxylamines are also available. A shortcoming of the present method in comparison to the classical oxime condensation is the size of required 2-FBPA motif. Although for most applications this should present no difficulties, examples where the compactness of the oxime is critical (such as, for example, as a functional isostere of peptide bonds)^37^ would not be possible. The ability to run conjugations at 1 : 1 ratios of partners at biological pH means that either or both components can be complex, precious materials. Although we have focused on oxime condensation for proof-of-concept the coordinating properties of boron in aqueous media could potentially be exploited to accelerate other important reactions whose rates are limited by the kinetics of Schiff base formation.^11^
