# Supplementary material for: Boronic acids facilitate rapid oxime condensations at neutral pH
Source: Chem Sci. 2015 Apr 13;6(6):3329–33. doi: 10.1039/c5sc00921a (PMC5656983; doi:10.1039/c5sc00921a)
Supplement: Supplementary file 1 [file SC-006-C5SC00921A-s001.pdf]

Electronic Supplementary Material (ESI) for Chemical Science.
This journal is © The Royal Society of Chemistry 2015

Boronic Acids Facilitate Rapid Oxime Condensations at Neutral pH

Electronic Supplementary Information

Pascal Schmidt, Cedric Stress, Dennis Gillingham*

Department of Chemistry, University of Basel, St. Johanns-Ring 19, CH-4056 Basel, Switzerland

* To whom correspondence should be addressed. Email: dennis.gillingham@unibas.ch

Table of Contents

1. Synthesis S3

    1.1 General Experimental Information S3

    1.2 Experimental and Characterization data S4

2. NMR studies on boron accelerated oxime formation S13

    2.1 General Information S13

    2.2 Experimental S13

3. HPLC studies on boron accelerated oxime formation S15

    3.1 General Information S15

    3.2 Corresponding HPLC traces and ESI-MS spectra S16

4. Fluorescent Quench Assay S25

    4.1 General S25

    4.2 Experimental setup S25

    4.3 Data processing S25

    4.4 Results S26

5. NMR stability and reversibility studies S32

    5.1 General Information S32

    5.2 ^{1}H-NMR stability test of oxime **2a** S32

    5.3 ^{1}H-NMR reversibility assay of oxime **2a** S34

6. Boron-assisted ligation in complex environments S39

    6.1 General Information S39

    6.2 Corresponding UPLC/HPLC traces and ESI-MS spectra S40

7. NMR spectra S46

8. References S61

1. Synthesis

1.1 General Experimental Information

1,4-dioxane was freshly distilled from sodium benzophenone ketyl under dry nitrogen prior to use. Other reagents and solvents used were of analytical grade and purchased from Sigma-Aldrich, Alfa Aesar, Apollo Scientific Ltd., Fluka, Fluorochem, ABCR or Acros and used as received. Buffers and HPLC eluents were prepared with nanopure water (resistivity up to 18.2 MΩ). Analytical TLC was performed on Silica gel 60 F_{254} pre-coated aluminium sheets (Merck) or glass plates (Merck) and visualized by fluorescence quenching under UV light at 254 nm. Flash chromatography was performed on SilicaFlash® gel P60 40-63 μm (230-400 mesh) (SiliCycle, Quebec). Concentration under reduced pressure was performed by rotatory evaporation at 40°C (unless otherwise specified).^{1}H, ^{13}C, ^{11}B and 2D-NMR spectra were acquired on a BrukerAvance (400, 500 or 600 MHz proton frequency) spectrometer at 298.15 K. Chemical shifts (δ values) relative to TMS are referenced to the solvent's residual peak and reported in ppm. Multiplicities are reported as follows: *s* = singlet, s_{br} = broad singlet, d = doublet, t = triplet, q = quartet, quint. = quintet, m = multiplet or unresolved and coupling constant *J* in Hz. ESI-MS spectra were recorded on a Bruker Esquire3000+ spectrometer by direct injection in positive polarity of the ion trap detector. High resolution mass spectra (HRMS) were recorded by the mass spectrometric service of the University of Basel on a Bruker maXis 4G QTOF ESI mass spectrometer. Preparative RP-HPLC was carried out on a Shimadzu Prominence UFLC Preparative Liquid Chromatograph using a C18 column (Gemini NX5u, 110Å, AXIA, 250 x 21.2 mm) from Phenomenex with a flow rate of 20 mL/min monitoring and collecting the products at 254 nm if not otherwise specified. The crude compound mixtures were injected as DMSO solutions. Analytical RP-HPLC was carried out on a Shimadzu Prominence UFLC Liquid Chromatograph using a C18 column (Eclipse XDB, 5 μm, 4.6 x 150 mm) from Agilent with a flow rate of 1 mL/min monitoring and collecting the products at 254 nm or 280 nm if not otherwise specified. UPLC-MS was carried out on an Agilent 1290 Infinity system equipped with an Agilent 6130 Quadrupole LC/MS using a C18 column (ZORBAX Eclipse Plus RRHD, 1.8 μm, 2.1 x 50 mm) from Agilent with a flow rate of 0.45 mL/min at 40°C. Elution was done with 0.1% (v/v) formic acid in water/10% (v/v) MeCN (A) and 0.1% (v/v) formic acid in MeCN/10% (v/v) water (B) using the following gradient: 5-90% (B) in 3.5 min, 90% (B) in 1 min, detection at 254 nm, ESI-MS in positive ion mode of the ion trap.

1.2 Experimental and Characterization data

(*E*)-(2-(((benzyloxy)imino)methyl)phenyl)boronic acid (2a)

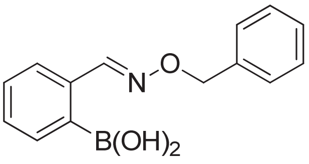

2-Formylphenylboronic acid (49.0 mg, 0.310 mmol, 1.00 eq.) was dissolved in dry MeOH (3 mL) followed by the addition of O-benzylhydroxylamine · HCl (50.0 mg, 0.310 mmol, 1.00 eq.) which was washed down the flask with another 2 mL of dry MeOH. The progress of the reaction was monitored by UPLC-MS. After 15.5 h full conversion of the starting aldehyde was achieved and the solvent was removed under reduced pressure. The crude was purified employing preparative RP-HPLC (gradient: 5% MeCN in H_2O, 0.1% TFA for 3 min, 5-95 % MeCN in H_2O, 0.1% TFA for 25 min) with a flow rate of 20 mL/min monitoring and collecting the product at 254 nm (t_R = 20.7 min). The product fractions were re-analyzed by UPLC-MS and lyophilized. The oxime was obtained as a fluffy white solid (52.0 mg, 0.203 mmol, 65%). TLC (cyclohexane/EtOAc 1:1) R_f = 0.45. ^{1}H-NMR (400 MHz, CD_3CN) δ/ppm: 8.49 (*s*, 1H), 7.74-7.70 (*m*, 1H), 7.63-7.60 (*m*, 1H), 7.46-7.36 (*m*, 6H), 7.36-7.31 (*m*, 1H), 6.49 (*s*, 2H) 5.17 (s, 2H). It has to be mentioned that the ^{1}H-NMR spectral data of the oxime boronic acid **2a** in $CDCl_3$ contained product signals along with some boroxine or other unidentified boronic acid related signals. Measuring the sample in CD_3CN proved to give a cleaner spectrum with no other species present. ^{13}C-NMR (101 MHz, $CDCl_3$) δ/ppm: 153.93, 138.31, 136.47, 134.99, 133.29, 130.93, 129.97, 128.78, 128.76, 128.67, 128.59, 76.73. ^{11}B-NMR (128 MHz, $CDCl_3$) δ/ppm: 29.00. ^{11}B-NMR (128 MHz, CD_3CN) δ/ppm: 29.37. HRMS (ESI): $C_{14}H_{14}BNNaO_3^+$ *calcd.*: 278.0959, *found*: 278.0963.

(*E*)-(2-(1-((benzyloxy)imino)ethyl)phenyl)boronic acid (2e)

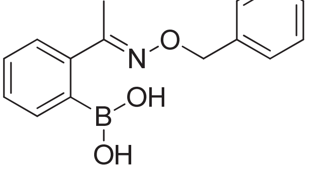

2-Acetylphenylboronic acid (2-APBA) (21.5 mg, 0.129 mmol, 1.00 eq.) was dissolved in dry MeOH (1.5 mL) followed by the addition of O-benzylhydroxylamine · HCl (21.0 mg, 0.130 mmol, 1.01 eq.) which was washed down the flask with another 1 mL of dry MeOH. The progress of the reaction was monitored by UPLC-MS. After 18 h full conversion of the starting ketone was achieved and the solvent was removed under reduced pressure. The crude was purified employing preparative RP-

HPLC (gradient: 2% MeCN in H_2O, 0.1% TFA for 3 min, 2-80 % MeCN in H_2O, 0.1% TFA for 25 min) with a flow rate of 20 mL/min monitoring and collecting the product at 254 nm (t_R = 22.4 min). The product fractions were re-analyzed by UPLC-MS and lyophilized. The oxime **2e** was obtained as a fluffy white solid (20.0 mg, 0.074 mmol, 58%, 98% purity). TLC (19:1 CH_2Cl_2/MeOH) R_f = 0.36. ^{1}H-NMR (400 MHz, DMSO-d_6) δ/ppm: 7.70-7.60 (*m*, 1H), 7.47-7.37 (*m*, 3H), 7.30-7.19 (*m*, 5H), 5.13 (*s*, 2H), 2.21 (*s*, 3H). ^{13}C-NMR (101 MHz, DMSO-d_6) δ/ppm: 162.62, 139.55, 136.63, 132.02, 129.51, 128.98, 128.32, 128.12, 128.01, 126.43, 75.31, 14.70 (one signal for the carbon atom directly attached to the boron was not observed due to the quadrupolar relaxation of the boron atom). ^{11}B-NMR (128 MHz, DMSO-d_6) δ/ppm: 25.15 (after background spectrum subtraction). HRMS (ESI): $C_{15}H_{16}BNNaO_3^+$ *calcd.*: 292.1115, *found*: 292.1120, $C_{16}H_{18}BNNaO_3^+$ *calcd.*: 306.1272, *found*: 306.1277, $C_{17}H_{20}BNNaO_3^+$ *calcd.*: 320.1428, *found*: 320.1433.

2-(4,4,5,5-tetramethyl-1,3,2-dioxaborolan-2-yl)benzaldehyde (3)

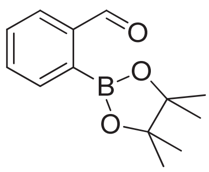

A 25 mL flask equipped with a Dean-Stark apparatus was charged with 2-formylphenylboronic acid (250 mg, 1.67 mmol, 1.00 eq.) and pinacol (216.7 mg, 1.83 mmol, 1.10 eq.) in benzene (10 mL). The mixture was refluxed for 5 h whereupon a ^{1}H-NMR aliquot indicated full conversion of the starting boronic acid. The mixture was dried with Na_2SO_4 (~2 g), filtered and washed with benzene. The solvent was removed under reduced pressure to give the title compound **3** as light yellow oil (387 mg, 1.67 mmol, quant.). TLC (cyclohexane/EtOAc 5:1) R_f = 0.50. ^{1}H-NMR (400 MHz, $CDCl_3$) δ/ppm: 10.54 (*s*, 1H), 7.97-7.94 (*m*, 1H), 7.87-7.84 (*m*, 1H), 7.62-7.53 (*m*, 2H), 1.40 (*s*, 12H). ^{13}C-NMR (101 MHz, $CDCl_3$) δ/ppm: 194.79, 141.39, 135.61, 133.13, 130.88, 128.04, 84.55, 25.02 (one signal for the carbon atom directly attached to the boron was not observed due to the quadrupolar relaxation of the boron atom). ^{11}B-NMR (128 MHz, $CDCl_3$) δ/ppm: 31.28. HRMS (ESI): $C_{13}H_{18}BO_3^+$ *calcd.*: 233.1344, *found*: 233.1344, $C_{13}H_{17}BNaO_3^+$ *calcd.*: 255.1163, *found*: 255.1164.

(*E*)-2-(4,4,5,5-tetramethyl-1,3,2-dioxaborolan-2-yl)benzaldehyde O-benzyl oxime (2f)

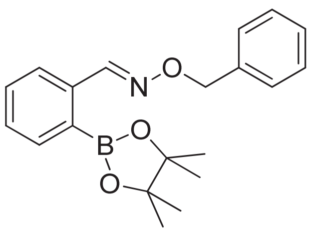

Compound **3** (25.0 mg, 0.106 mmol, 1.00 eq.) was dissolved in dry MeOH (2 mL) followed by the addition of O-benzylhydroxylamine · HCl (17.0 mg, 0.106 mmol, 1.00 eq.) which was washed down the flask with another 1 mL of dry MeOH. The progress of the reaction was monitored by UPLC-MS. After 14.5 h full conversion of the starting aldehyde was achieved and the solvent was removed under reduced pressure. The crude was purified by flash chromatography (10:1 cyclohexane/EtOAc). The oxime **2f** was obtained as a colorless (33.0 mg, 0.098 mmol, 92%, 98% purity.). TLC (cyclohexane/EtOAc 5:1) R_f = 0.63. ^{1}H-NMR (400 MHz, $CDCl_3$) δ/ppm: 8.95 (*s*, 1H), 7.98-7.95 (*m*, 1H), 7.86-7.83 (*m*, 1H), 7.47-7.41 (*m*, 3H), 7.41-7.35 (*m*, 3H), 7.35-7.32 (*m*, 1H), 5.24 (*s*, 2H), 1.34 (*s*, 12H). ^{13}C-NMR (101 MHz, $CDCl_3$) δ/ppm: 150.46, 138.03, 137.97, 136.31, 131.18, 128.84, 128.53, 127.98, 125.45, 84.15, 76.39, 25.01 (HMQC indicated that the protons at 7.47 and 7.37 ppm show a cross peak for the carbon at 128.84 ppm which is an overlay of two carbon signals - one signal for the carbon atom directly attached to the boron was not observed due to the quadrupolar relaxation of the boron atom). ^{11}B-NMR (128 MHz, $CDCl_3$) δ/ppm: 31.09. HRMS (ESI): $C_{20}H_{25}BNO_3^+$ *calcd.*: 338.1922, *found*: 338.1923, $C_{20}H_{24}BNNaO_3^+$ *calcd.*: 360.1741, *found*: 360.1744, $C_{20}H_{25}BKNO_3^+$ *calcd.*: 376.1481, *found*: 376.1479.

1-(2-(4,4,5,5-tetramethyl-1,3,2-dioxaborolan-2-yl)phenyl)ethanone (4)

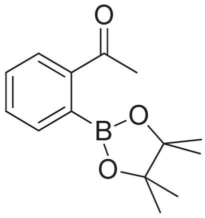

To a mixture of *bis*-(pinacolato)diboron (280 mg, 1.10 mmol, 1.50 eq.), NaOAc (241 mg, 2.94 mmol, 4.00 eq.) and $Pd(PPh_3)_2Cl_2$ (25.8 mg, 0.037 mmol, 0.05 eq.) in 1,4-dioxane (5.0 mL) was added 2-Bromoacetophenone (100 µL, 0.734 mmol, 1.00 eq.). The resulting mixture was stirred at 90°C for 15 h and the solvent was removed under reduced pressure to give a grey semisolid. The crude was purified by flash chromatography on a Biotage Isolera Four device (5:1 to 4:1 cyclohexane/EtOAc). The product **4** was isolated as yellow oil (83 mg, 0.331 mmol, 45%, 98% purity). TLC (cyclohexane/EtOAc 5:1) R_f = 0.18. ^{1}H-NMR (400 MHz, $CDCl_3$) δ/ppm: 7.83 (*dt*, J = 7.8, 0.9 Hz, 1H), 7.57-7.49 (*m*, 2H), 7.47-7.40 (*m*, 1H), 2.61 (*s*, 3H), 1.44 (*s*, 12H). ^{13}C-NMR (101 MHz, $CDCl_3$)

δ/ppm: 199.94, 140.63, 132.62, 132.35, 129.00, 128.57, 83.85, 25.63, 25.04 (one signal for the carbon atom directly attached to the boron was not observed due to the quadrupolar relaxation of the boron atom). ^{11}B-NMR (128 MHz, $CDCl_3$) δ/ppm: 31.26. HRMS (ESI): $C_8H_9BNaO_3^+$ *calcd.*: 187.0537, *found*: 187.0540 (free Boronic acid), $C_{14}H_{19}BNaO_3^+$ *calcd.*: 269. 1319, *found*: 269.1320. LRMS (ESI): 147.1 $[M-C_6H_{11}O]^+$, 247.1 $[M+H]^+$, 269.2 $[M+Na]^+$, 285.1 $[M+K]^+$, 515.4 $[2M+Na]^+$.

(*E*)-1-(2-(4,4,5,5-tetramethyl-1,3,2-dioxaborolan-2-yl)phenyl)ethanone O-benzyl oxime (2g)

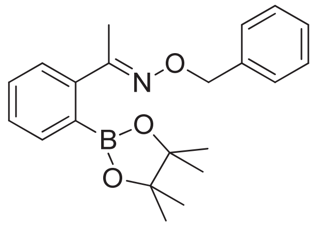

4 (20.0 mg, 0.080 mmol, 1.00 eq.) was dissolved in dry MeOH (1.5 mL) followed by the addition of O-benzylhydroxylamine · HCl (13.0 mg, 0.081 mmol, 1.01 eq.) which was washed down the flask with another 1 mL of dry MeOH. The progress of the reaction was monitored by UPLC-MS. After 18 h full conversion of the starting ketone was achieved and the solvent was removed under reduced pressure. The crude was purified by flash chromatography (6:1 cyclohexane/EtOAc). The early fractions contained 12 mg of desired product. The fractions which had to be eluted with (19:1 CH_2Cl_2/MeOH) didn't contain the free boronic acid product as expected, instead another 10 mg of desired product were isolated. This observation is probably attributed to a charged product species containing an additional methoxy group on the boron atom which changes its polarity significantly. The oxime **2g** was obtained as colorless oil (22.0 mg, 63.0 µmol, 79%). TLC (6:1 cyclohexan/EtOAc) R_f = 0.52. NMR spectroscopy indicated a mixture of stereoisomers of 11:1.

Major stereoisomer:

^{1}H-NMR (400 MHz, $CDCl_3$) δ/ppm: 7.72 (*dd*, J = 7.5, 0.9 Hz, 1H), 7.49-7.37 (*m*, 3H), 7.37-7.26 (*m*, 4H), 7.25-7.06 (*m*, 1H), 5.23 (*s*, 2H), 2.27 (*s*, 3H), 1.34 (*s*, 12H). ^{13}C-NMR (101 MHz, $CDCl_3$) δ/ppm: 159.26, 143.32, 138.37, 134.98, 130.44, 128.43, 127.99, 127.72, 127.59, 127.38, 83.95, 75.94, 24.98, 17.11. ^{11}B-NMR (128 MHz, $CDCl_3$) δ/ppm: 31.65. HRMS (ESI): $C_{21}H_{27}BNO_3^+$ *calcd.*: 352.2079, *found*: 352.2085, $C_{21}H_{26}BNNaO_3^+$ *calcd.*: 374.1898, *found*: 374.1906.

Minor stereoisomer:

^{1}H-NMR (400 MHz, $CDCl_3$) δ/ppm: 7.84 (*dd*, J = 7.2, 1.0 Hz, 1H), 7.49-7.37 (*m*, 3H), 7.37-7.26 (*m*, 4H), 7.25-7.06 (*m*, 1H), 5.04 (*s*, 2H), 2.18 (*s*, 3H), 1.30 (*s*, 12H). ^{13}C-NMR (101 MHz, $CDCl_3$) δ/ppm: 142.84, 138.92, 135.41, 130.94, 128.19, 127.32, 127.96, 127.23, 126.14, 83.88, 75.15, 24.90, 23.02. ^{11}B-NMR (128 MHz, $CDCl_3$) δ/ppm: 31.65.

***tert*-butyl (3-bromopropyl)carbamate**

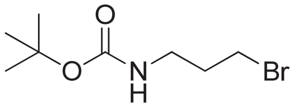

This compound was synthesized according to a published procedure.[1] Analytical data was in agreement with the reported data.

***tert*-butyl (3-(4-bromo-3-formylphenoxy)propyl)carbamate (5)**

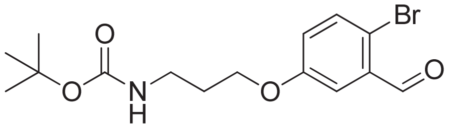

An oven dried 25 mL flask equipped with a stir bar was charged with 2-bromo-5-hydroxy benzaldehyde (480 mg, 2.34 mmol, 1.00 eq.), *tert*-butyl (3-bromopropyl)carbamate[1] (575 mg, 2.37 mmol, 1.01 eq.) and dissolved in 6 mL of dry DMF. To the stirring solution was added K_2CO_3 (970 mg, 7.02 mmol, 3.00 eq.) whereupon the color turned to bright yellow. The heterogeneous mixture was stirred at 80°C and the progress of the reaction was monitored by UPLC-MS. After 21 h (still starting benzaldehyde derivative) water (50 mL) was added and the mixture extracted with EtOAc (3 x 15 mL). The combined organics were dried over Na_2SO_4, filtered and the solvent removed under reduced pressure to give a slightly orange oil. The crude was purified twice by flash chromatography (5:1 cyclohexane/EtOAc) but the starting benzaldehyde derivative (8% according to ^{1}H-NMR) could not be removed effectively from the product. Compound **5** was obtained as slightly yellow oil (654 mg, 1.68 mmol, 72%) in 92% purity. TLC (cyclohexane/EtOAc 5:1) R_f = 0.22. ^{1}H-NMR (400 MHz, $CDCl_3$) δ/ppm: 10.30 (*s*, 1H), 7.52 (*d*, J = 8.8 Hz, 1H), 7.40 (*d*, J = 3.2 Hz, 1H), 7.03 (*dd*, J = 8.8, 3.2 Hz, 1H), 4.69 (s_{br}, 1H), 4.05 (*t*, J = 6.0 Hz, 2H), 3.36-3.27 (*m*, 2H), 1.99 (*quint*, J = 6.3 Hz, 2H), 1.44 (*s*, 9H). ^{13}C-NMR (101 MHz, $CDCl_3$) δ/ppm: 191.90, 158.60, 156.15, 134.75, 134.09, 123.54, 118.18, 113.51, 79.59, 66.36, 37.91, 29.60, 28.55. HRMS (ESI): $C_{10}H_{13}BrNO_2^+$ *calcd.*: 258.0124, *found*: 258.0121, $C_{15}H_{20}BrNNaO_4^+$ *calcd.*: 380.0468, *found*: 380.0465, $C_{16}H_{24}BrNNaO_5^+$ *calcd.*: 412.0730, *found*: 412.0727.

(*E*)-*N*-(3-(4-bromo-3-formylphenoxy)propyl)-4-((4-(dimethylamino)phenyl)diazenyl)benzenesulfonamide (6)

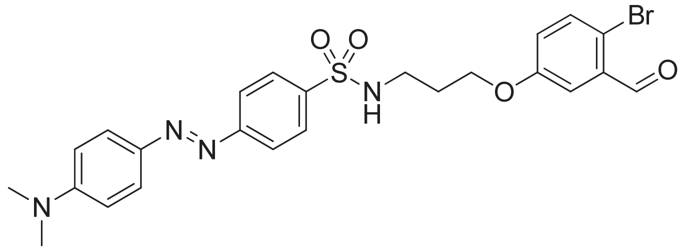

Compound **5** (113 mg, 0.284 mmol, 1.00 eq., 90% purity) was dissolved in dry CH_2Cl_2 (2 mL) followed by the addition of TFA (0.50 mL). The mixture was stirred at room temperature and the progress of the reaction was monitored by UPLC-MS. After 25 min full conversion of the starting material was observed. The solvents were removed under reduced pressure to give an orange oil which was extensively dried under high vacuum. The mass balance was too high which was attributed to residual TFA in the sample. Without further purification the crude TFA salt of the free amine was dissolved in dry EtOH (10 mL) and cooled to 0°C. To the stirring solution was added Dabsyl chloride (123 mg, 323 mmol, 1.00 eq., 97.5 % purity) followed by NEt_3 (2 mL). It has to be noticed that the excess of base was chosen to neutralize the TFA salt of the free amine as well as the residual TFA which could not be removed by high vacuum treatment. The mixture was allowed to warm to room temperature and the progress of the reaction was monitored by UPLC-MS. The starting amine was still present after 18 h at which point the reaction was stopped and the solvent was removed under reduced pressure. The UPLC-MS chromatograms after 1 h, 3 h and 18 h were identical in terms of starting material/product ratio and the reaction could have been stopped earlier. The crude was purified by flash chromatography on a Biotage Isolera Four device (3:1 cyclohexane/EtOAc, isocratic). The product was isolated as a red solid (41.0 mg, 69.2 µmol, 21%, 92% purity). An analytical sample was purified employing preparative RP-HPLC (gradient: 20% MeCN in H_2O, 0.1% TFA for 3 min, 20-95 % MeCN in H_2O, 0.1% TFA for 25 min) with a flow rate of 20 mL/min monitoring and collecting the product at 254 nm (t_R = 23.9 min). The product fractions were re-analyzed by UPLC-MS and lyophilized to give **6** as a dark red crystalline solid. TLC (cyclohexane/EtOAc 1:1) R_f = 0.55. ^{1}H-NMR (400 MHz, $CDCl_3$) δ/ppm: 10.24 (*s*, 1H), 7.96-7.89 (*m*, 4H), 7.88-7.82 (*m*, 2H), 7.49 (*d*, J = 8.8 Hz, 1H), 7.30 (*d*, J = 3.2, 1H), 6.95 (*dd*, J = 8.8, 3.2 Hz, 1H), 6.82-6.76 (*m*, 2H), 4.80 (*t*, J = 6.6 Hz, 1H), 3.98 (*t*, J = 5.8 Hz, 2H), 3.23 (*q*, J = 6.2 Hz, 2H), 3.14 (*s*, 6H), 1.97 (*quint.*, J = 6.2 Hz, 2H). ^{13}C-NMR (101 MHz, $CDCl_3$) δ/ppm: 191.78, 158.21, 155.17, 153.44, 143.54, 139.23, 134.78, 134.05, 128.18, 126.37, 123.36, 122.59, 118.37, 113.46, 112.00, 65.81, 40.63, 40.61, 29.16. HRMS (ESI): $C_{24}H_{26}BrN_4O_4S^+$ *calcd.*: 545.0853, *found*: 545.0842, $C_{24}H_{25}BrN_4NaO_4S^+$ *calcd.*: 567.0672, *found*: 567.0666, $C_{25}H_{29}BrN_4NaO_5S^+$ *calcd.*: 599.0934, *found*: 599.0929.

(*E*)-(4-(3-(4-((4-(dimethylamino)phenyl)diazenyl)phenylsulfonamido)propoxy)-2-formylphenyl)boronic acid (7)

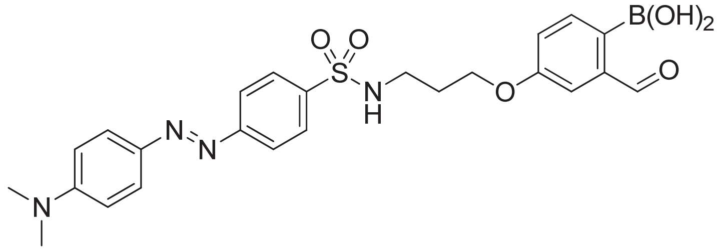

A flame dried 5 mL Schlenk tube under argon equipped with a stir bar was charged with compound **6** (9.00 mg, 17.0 mmol, 1.00 eq.) and dissolved in 1 mL of freshly distilled 1,4-dioxane. To the stirring solution was added *bis*-(pinacolato)diboron (8.60 mg, 34.0 mmol, 2.05 eq.), NaOAc (4.30 mg, 52 mmol, 3.18 eq.) and $Pd(PPh_3)_2Cl_2$ (1.20 mg, 2.0 mmol, 0.10 eq.) and washed down the pressure tube with 0.5 mL of 1,4-dioxane. The pressure tube was closed and the mixture heated to 90°C. The progress of the reaction was monitored by UPLC-MS and after 16 h the mixture was allowed to cool to room temperature and filtered over a plug of cotton. The solvent was then removed under reduced pressure to give 23 mg of a crude orange residue. The crude was purified employing preparative RP-HPLC (gradient: 10% MeCN in H_2O, 0.1% TFA for 13 min, 10-98 % MeCN in H_2O, 0.1% TFA for 30 min) with a flow rate of 20 mL/min monitoring and collecting the product at 254 nm (t_R = 31.4 min). The product fractions were re-analyzed by UPLC-MS and lyophilized to give **7** as a fluffy dark red solid (3.7 mg, 6.25 μmol, 38%). The compound isolated was pure according to a UPLC-MS run and HRMS (ESI) (see chromatogram and corresponding ESI-MS trace below). ^{1}H-NMR (400 MHz, DMSO-d_6) δ/ppm: 10.18 (*s*, 1H), 7.94-7.85 (*m*, 4H), 7.85-7.76 (*m*, 3H), 7.58 (*d*, J = 8.1 Hz, 1H), 7.32 (*d*, J = 2.5 Hz, 1H), 7.14 (*dd*, J =8.3, 2.5 Hz, 1H), 6.88-6.84 (*m*, 2H), 4.03 (*t*, J = 6.2 Hz, 2H), 3.09 (*s*, 6H), 3.01-2.93 (*m*, 2H), 1.89-1.80 (*m*, 2H). ^{13}C-NMR (101 MHz, DMSO-d_6) δ/ppm: 194.22, 159.14, 154.49, 153.14, 142.62, 141.35, 140.17, 135.58, 127.80, 125.44, 122.27, 119.61, 112.99, 111.63, 64.77, 39.88, 39.35 (overlapping with DMSO signal - identified by DEPT135), 28.79 (one signal for the carbon atom directly attached to the boron was not observed due to the quadrupolar relaxation of the boron atom). ^{11}B-NMR (128 MHz, DMSO-d_6) δ/ppm: 20.11 (measured in a 701-PQ NMR tube using 2048 scans and after background spectrum subtraction). HRMS (ESI): $C_{24}H_{28}BN_4O_6S^+$ *calcd.*: 511.1817, *found*: 511.1826, $C_{25}H_{30}BN_4O_6S^+$ *calcd.*: 525.1974, *found*: 525.1979, $C_{24}H_{27}BN_4NaO_6S^+$ *calcd.*: 533.1637, *found*: 533.1638.

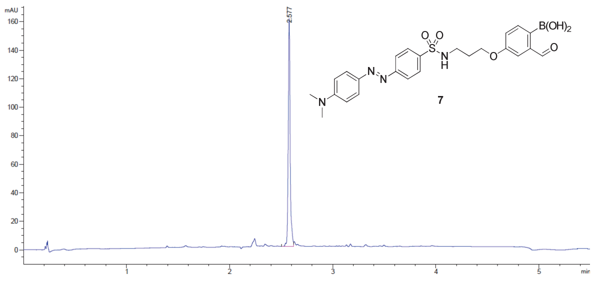

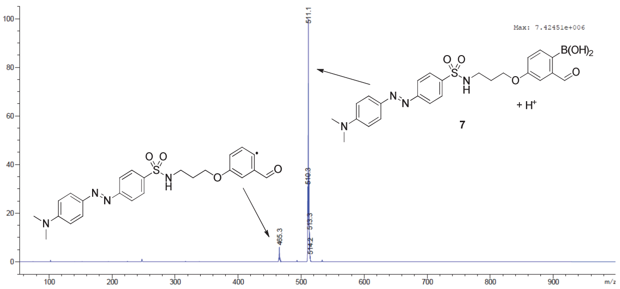

5-(*N*-(2-(aminooxy)ethoxy)sulfamoyl)-2-(6-(diethylamino)-3-(diethyliminio)-3H-xanthen-9-yl)benzenesulfonate (8)

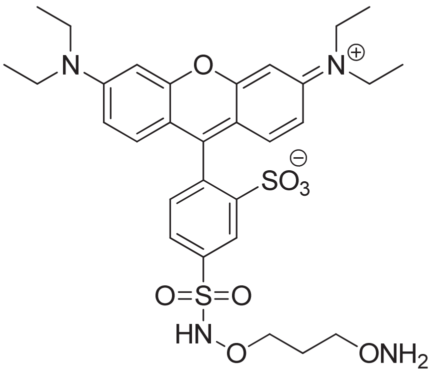

An oven dried 10 mL flask under argon was charged with *O*,*O*'-1,3-Propanediylbishydroxylamine dihydrochloride (13.0 mg, 0.072 mmol, 1.10 eq.) and dry EtOH (4 mL). The suspension was cooled to 0°C before triethylamine (46 µL, 0.330 mmol, 5.00 eq.) was added. After 5 min Sulforhodamine B acid chloride (40.0 mg, 66.0 µmol, 1.00 eq.) was added as a solid and the reaction allowed to warm to room temperature. The mixture was stirred for 2 h and UPLC-MS indicated a product peak (647.3 Da) along with a peak corresponding to the dimer (1187.3 Da). The solvent was removed under reduced pressure to give a purple solid (86 mg). The crude was purified employing preparative RP-HPLC (gradient: 25% MeCN in H_2O, 0.1% TFA for 3 min, 25-80 % MeCN in H_2O, 0.1% TFA for 25 min) with a flow rate of 20 mL/min monitoring and collecting the product at 254 nm (t_R = 13.0 min). The product fraction were re-analyzed by UPLC-MS and lyophilized to give **8** as a dark purple fluffy solid (11.6 mg, 18.0 µmol, 27%). ^{1}H-NMR (400 MHz, DMSO-d_6) δ/ppm: 10.78 (*s*, 1H), 10.50 (s_{br}, 2H), 8.50 (*d*, J = 1.9 Hz, 1H), 8.01 (*dd*, J = 8.0, 1.9 Hz, 1H), 7.57 (*d*, J = 8.0 Hz, 1H), 7.06 (*dd*, J = 2.4, 9.5 Hz, 2H), 6.96 (*d*, J = 2.4 Hz, 2H), 6.93 (*d*, J = 9.4 Hz, 2H), 4.07-4.00 (*m*, 4H), 3.70-3.58 (*m*, 8H), 1.94 (*quint.*, J = 6.2 Hz, 2H), 1.21 (*t*, J = 7.0 Hz, 12H). ^{13}C-NMR (101 MHz, DMSO-d_6) δ/ppm: 157.08, 156.76, 155.04, 147.51, 138.12, 134.29, 132.41, 130.83, 127.52, 113.77, 113.34, 95.47, 72.80, 71.26, 45.27, 26.32, 12.48. HRMS (ESI): $C_{30}H_{39}N_4O_8S_2^+$ *calcd.*: 647.2204, *found*: 647.2206, $C_{30}H_{38}N_4NaO_8S_2^+$ *calcd.*: 669.2023, *found*: 669.2021.

(*E*)-*N*-(3-(aminooxy)propoxy)-4-((4-(dimethylamino)phenyl)diazenyl)benzenesulfonamide (9)

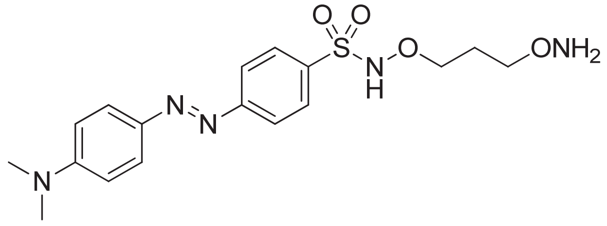

An oven dried flask under argon was charged with 4-(Dimethylamino)azobenzen-4'-sulfonyl chloride (111 mg, 0.333 mmol, 1.00 eq.), *O*,*O*'-1,3-Propanediylbishydroxylamine dihydrochloride (66.0 mg, 0.365 mmol, 1.10 eq.) and dry EtOH (12 mL). Triethylamine (170 µL, 1.22 mmol, 3.67 eq.) was

added and the resulting mixture was stirred at room temperature for 2 h. The solvent was removed under reduced pressure to give a red solid. The crude was adsorbed onto RP silica gel and purified by flash chromatography on a Biotage Isolera Four device (gradient: 0% MeCN for 4 min, 0-78 % MeCN for 32 min, 78-100% MeCN for 4 min) with a flow rate of 50 mL/min monitoring and collecting the product at 254 nm (t_R = 27.0 min). The pure fractions were lyophilized to give hydroxylamine **9** as an orange crystalline solid (51.0 mg, 130 µmol, 39%). ^{1}H-NMR (400 MHz, DMSO-*d*$_6$) δ/ppm: 10.46 (*s*, 1H), 8.00-7.91 (*m*, 4H), 7.88-7.80 (*m*, 2H), 6.90-6.82 (*m*, 2H), 5.92 (*s*, 2H), 3.93 (*t*, J = 6.4 Hz, 2H), 3.53 (*t*, J = 6.4 Hz, 2H), 3.10 (*s*, 6H), 1.76 (*quint.*, J = 6.4 Hz, 2H). ^{13}C-NMR (101 MHz, DMSO-*d*$_6$) δ/ppm: 155.25, 153.24, 142.62, 136.80, 129.40, 125.55, 122.01, 111.62, 73.55, 71.30, 39.85, 26.93. HRMS (ESI): $C_{17}H_{24}N_5O_4S^+$ *calcd.*: 394.1544, *found*: 394.1542, $C_{17}H_{23}N_5NaO_4S^+$ *calcd.*: 416.1363, *found*: 416.1358.

(*E*)-2-hydroxybenzaldehyde O-benzyl oxime (10)

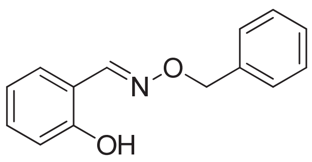

Salicylaldehyde (31.0 mg, 249 µmol, 1.00 eq.) was dissolved in dry MeOH (3 mL) followed by the addition of O-benzylhydroxylamine · HCl (40.0 mg, 0.248 mmol, 1.00 eq.) which was washed down the flask with another 2 mL of dry MeOH. The progress of the reaction was monitored by UPLC-MS. After 21 h full conversion of the starting aldehyde was achieved and the solvent was removed under reduced pressure. The crude was passed through a short plug of silica eluting the desired compound with cyclohexane/EtOAc (6:1). The oxime **10** was obtained as colorless oil which crystallized upon drying under high vacuum (56.0 mg, 248 µmol, quant.). TLC (cyclohexane/EtOAc 6:1) R_f = 0.59. ^{1}H-NMR (400 MHz, $CDCl_3$) δ/ppm: 9.80 (*s*, 1H), 8.22 (*s*, 1H), 7.44-7.32 (*m*, 5H), 7.30-7.24 (*m*, 1H), 7.14 (*dd*, J = 7.7, 1.6 Hz, 1H), 6.99-6.94 (*m*, 1H), 6.92-6.87 (*dt*, J = 7.6, 1.1 Hz, 1H), 5.18 (*s*, 2H). ^{13}C-NMR (101 MHz, $CDCl_3$) δ/ppm: 157.56, 152.05, 136.66, 131.41, 130.93, 128.77, 128.74, 128.54, 119.73, 116.88, 116.44, 77.02. HRMS (ESI): $C_{14}H_{14}NO_2^+$ *calcd.*: 228.1019, *found*: 228.1021.

2. NMR studies on boron accelerated oxime formation

2.1 General Information

The NMR experiments on boron accelerated oxime formation were performed at 288 K on a BrukerAvance III NMR spectrometer operating at 600.27 MHz, equipped with a ^{1}H-^{13}C/^{15}N/^{19}F-D cryogenic QCI probe head with *z*-axis pulsed field gradients. All 1D proton experiments were recorded using exactly the same parameters (relaxation delay of 0.5 s and an acquisition time of 2.0 s). The data was processed using TopSpin software from Bruker with a line broadening of 0.5 Hz and manually baseline corrected. Chemical shifts were referenced to the residual solvent peak of acetonitrile (1.94 ppm) and TMSP-d_4 (3-(trimethylsilyl)-2,2',3,3'-tetradeuteropropionic acid) was used as an internal standard. 2-FPBA was purchased from ABCR and O-benzylhydroxylamine was neutralized from its HCl salt according to a published procedure.[2] The pH (no correction for deuterium) was adjusted using a 827 pH Lab – Metrohm equipped with a glass minitrode which was pre-rinsed with D_2O.

2.2 Experimental

Stock solutions for both 2-FPBA and O-benzylhydroxylamine were freshly prepared in CD_3CN. A K_3PO_4 buffer (KP_i) stock solution was freshly prepared in D_2O adjusting the pH to 7.2 with concentrated DCl from ABCR (DCl, 38% wt% in D_2O, 99.5% atom%D). A TMSP-d_4 stock solution was freshly prepared in D_2O. All experiments were conducted at 15°C (288 K) in standard NMR tubes (throw away quality) which were rinsed thoroughly with D_2O and oven dried prior to use.

Setup for the ^{1}H-NMR experiment at 10 μM:

1. 440 μL of phosphate buffer (12.5 mM) were mixed inside the NMR tube with 50 μL of O-benzylhydroxylamine (110 μM) and 10 μL of TMSP-d_4 (0.275 mM). The sample was then locked to acetonitrile and shimmed before the addition of the 2-FPBA.
2. 50 μL of 2-FPBA (110 μM) were then added and the reaction followed by ^{1}H-NMR (32 scans) without additional shimming. The time between adding 2-FPBA to the NMR tube and recording of the last scan was determined to be ~ 3.5 min.

The final volume of the NMR sample was 550 μL with the following final concentrations:

10 μM in each O-benzylhydroxylamine and 2-FPBA, respectively. 10 mM phosphate buffer and 5 μM TMSP-d_4 with a ratio of 4:1 (10 mM KP_i in D_2O/CD_3CN).

For comparison a reference spectrum of 2-FPBA was recorded at 10 μM with the same solvent ratio of 4:1 (10 mM KP_i in D_2O/CD_3CN). The setup for the ^{1}H-NMR experiment at 1 μM was identical using

the same stock solutions except that the final solvent ratio was 54:1 (10 mM KP_i in D_2O/CD_3CN) and scans were increased to 128. The time between adding 2-FPBA to the NMR tube and recording of the last scan was determined to be ~ 8.5 min. An overview of the three ^{1}H-NMR spectra (selected region of 10.2 – 6.9 ppm) is shown in Scheme S1.

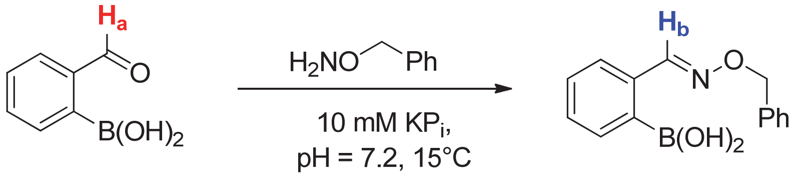

2-FPBA (**1**) **2a**

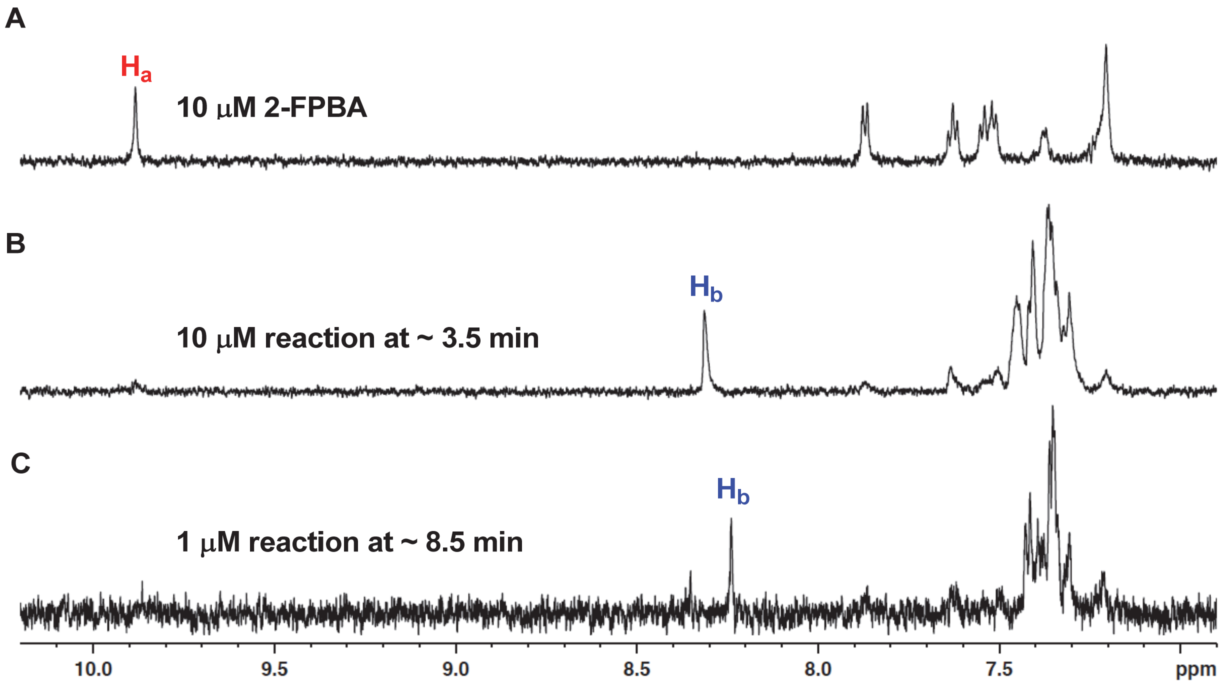

Scheme S1: All spectra were recorded at 15°C. (A) 10 µM 2-FPBA (9.88 ppm, H_a) reference spectrum in 4:1 (10 mM KP_i in D_2O/CD_3CN); (B) 10 µM reaction (8.31 ppm, H_b) spectrum in 4:1 (10 mM KP_i in D_2O/CD_3CN) after ~ 3.5 min; (C) 1 µM reaction (8.24 ppm, H_b) spectrum in 54:1 (10 mM KP_i in D_2O/CD_3CN) after ~ 8.5 min – the difference in the chemical shift is attributed to the solvent ratio.

3. HPLC studies on boron accelerated oxime formation

3.1 General Information

All substrate stock solutions were prepared in HPLC grade MeCN except for the LYRAG pentapeptide monohydroxylamine (H_3N-Aoa-Tyr-Arg-Ala-Gly-$CONH_2$)[3] which was prepared in nanopure water and stored at -20°C. The concentration of the peptide was determined using a NanoDrop 2000 spectrophotometer from Thermo Scientific with a path length of 1 mm and $\varepsilon_{(LYRAG\ 280\ nm)}$ = 1490 M^{-1} cm^{-1}. The different stock solutions for condensation were allowed to warm to room temperature, vortexed and centrifuged prior to mixing and combined in the following order in an PP HPLC vial (BGB, Part Number: PPSV0903K): 1) 100 mM potassium phosphate buffer pH = 7.2, 2) hydroxylamine, 3) aldehyde or ketone. The resulting solution was briefly mixed with a Gilson Pipetman before injection (time between mixing and injection is around 30 seconds). The reactions (Table S1 entries 1-9) were followed by RP-HPLC on a Shimadzu Prominence UFLC Liquid Chromatograph using a C18 column (Eclipse XDB, 5 µm, 4.6 x 150 mm) from Agilent (gradient: 2-98% MeCN containing 1% H_2O in H_2O containing 1% MeCN over 18 min, flow rate 1 mL/min, room temperature, injection volume of 20 µL, neutral conditions) monitoring and collecting the products at 254 nm or 280 nm if not otherwise specified.

Table S1: Probing the importance of boron positioning and substitution on oxime condensations.

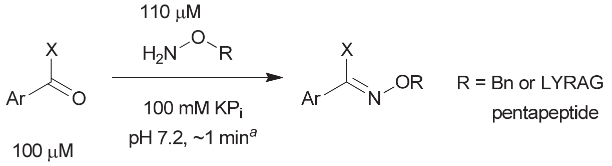

Entry	Ar	X	Product	Conc (µM)	Conv (%)[b]
1	Ph	H	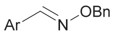	100	<5
2	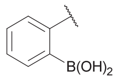	H	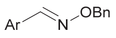 2a	100	>98
3	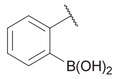	H	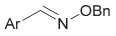 2a	10	>98
4	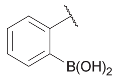	H	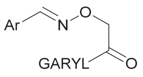 2b	100	>98
5	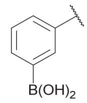	H	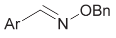 2c	100	<5
6	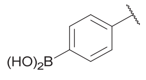	H	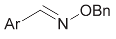 2d	100	<5
7	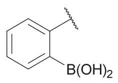	Me	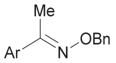 2e	100	94

**8**	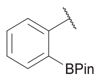	H	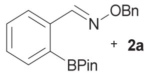 **2f**	100	>98[d]
**9**	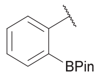	Me	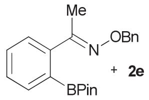 **2g**	100	>98

[a] Time is approximate since samples are injected directly after mixing; [b] Determined by reverse phase HPLC analysis under neutral conditions; [c] Injections at 90 minutes still show <5% conversion; [d] At the first injection approximately 10% of the pinacol ester oxime is observed, but only the hydrolyzed product is detected at 1.5 h. KP_i = potassium phosphate buffer.

3.2 Corresponding HPLC traces and ESI-MS spectra

Since the conversions in entries 1, 5 and 6 of Table S1 are <5% the corresponding HPLC data is not shown. It is noteworthy to mention that O-benzylhydroxylamine is not UV active and cannot be monitored under these HPLC conditions. The slight excess of 1.10 eq. is used to discriminate pipetting errors from the establishment of an equilibrium; a small excess will not affect the equilibrium but will negate the possibility of small pipetting errors leading to incomplete conversion. All conversion numbers are related to the disappearance of the peak area of 2-FPBA at 254 nm on the first injection after ~1 min. A reference chromatogram of 2-FPBA at 100 µM is shown in Figure S1.

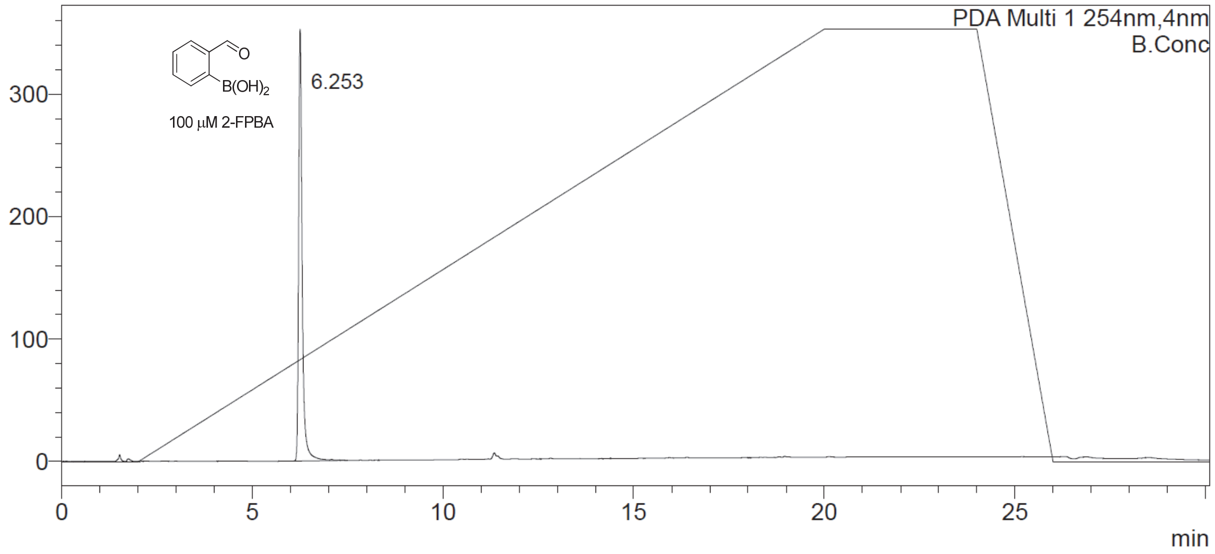

Figure S1: HPLC chromatogram of a 100 µM solution of 2-FPBA monitored at 254 nm including MeCN gradient which was identical for all reactions monitored by RP-HPLC under neutral conditions.

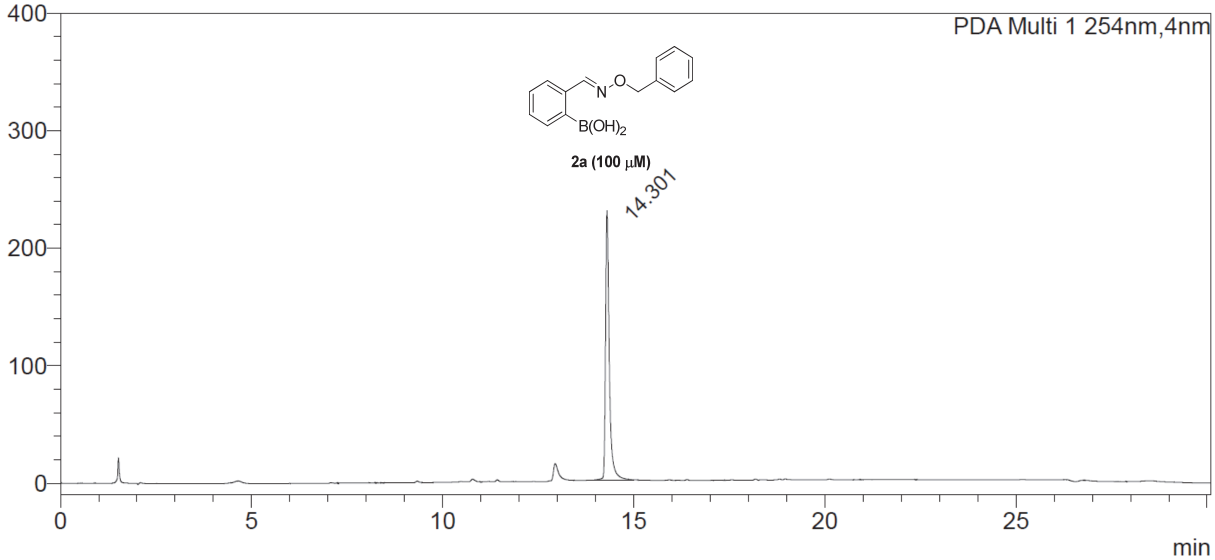

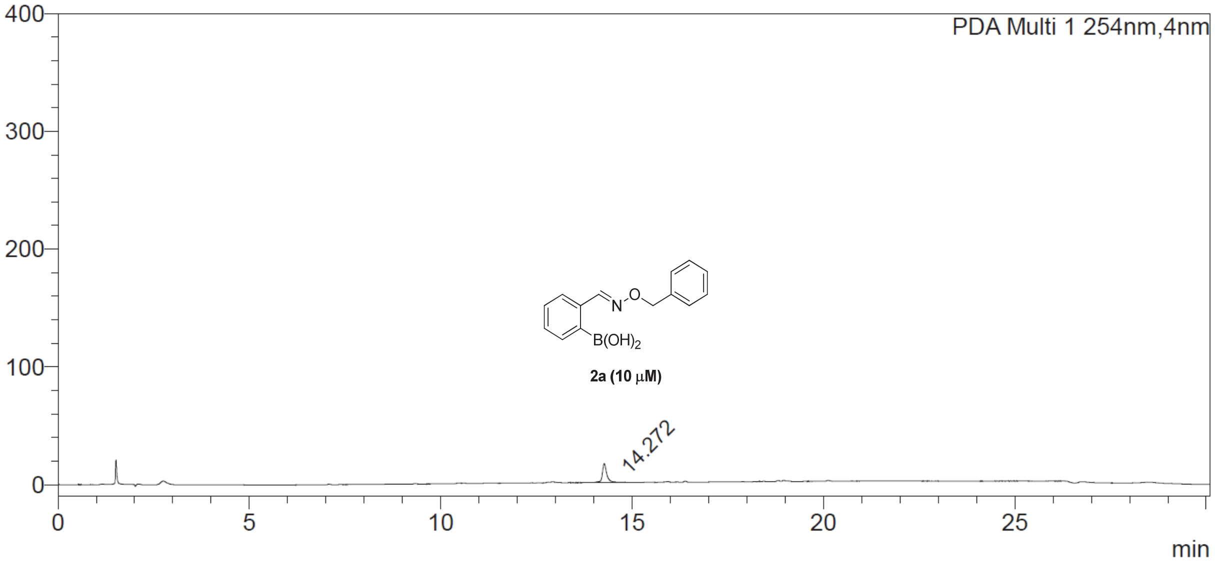

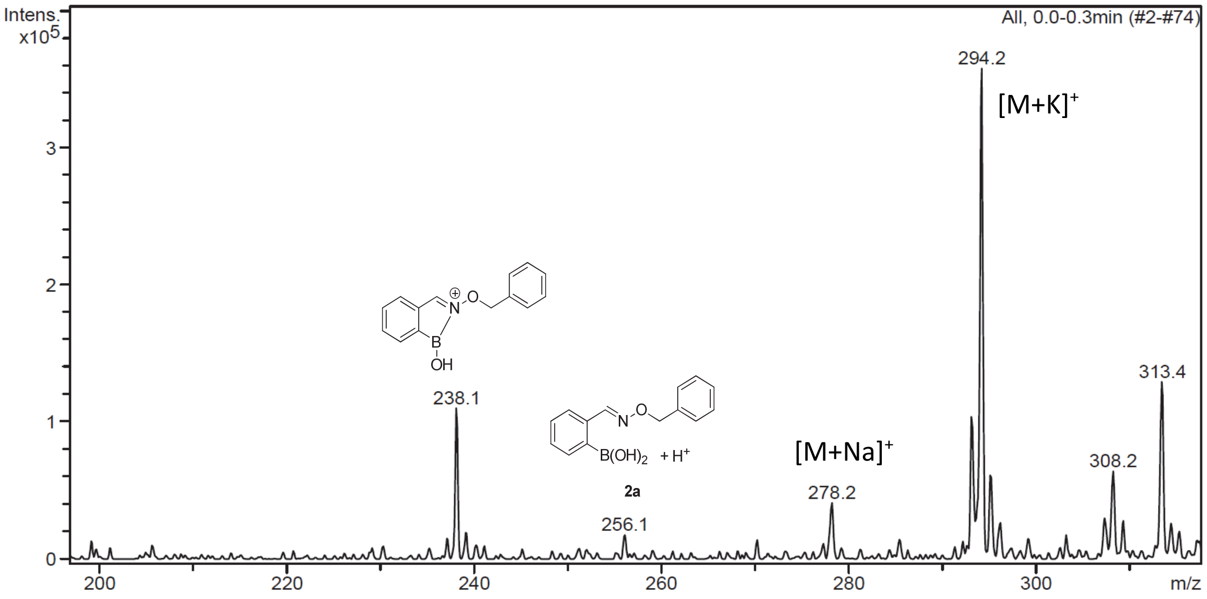

Table S1 Entries 2 and 3 | **Top:** HPLC trace at 254 nm after ~1 min at 100 μM (top) and 10 μM (middle) – full conversion of 2-FPBA is achieved along with the product peak of oxime **2a**; **Bottom:**

ESI-MS spectra of the peak at t_R = 14.3 min (100 μM). The major species observed is the potassium adduct and the charged oxime which lacks a hydroxyl group.

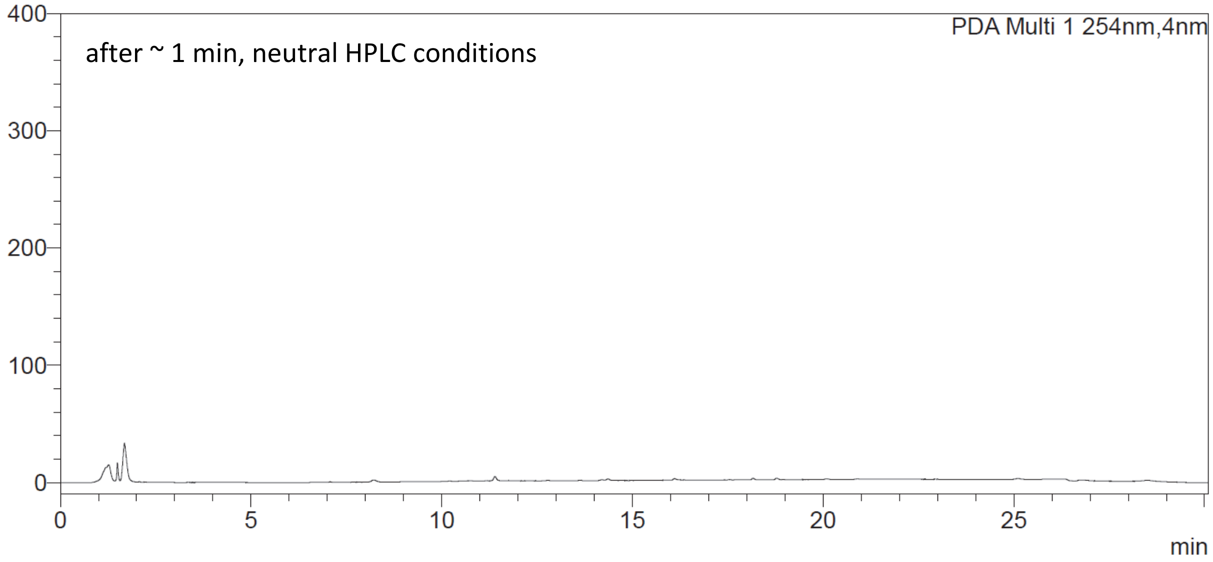

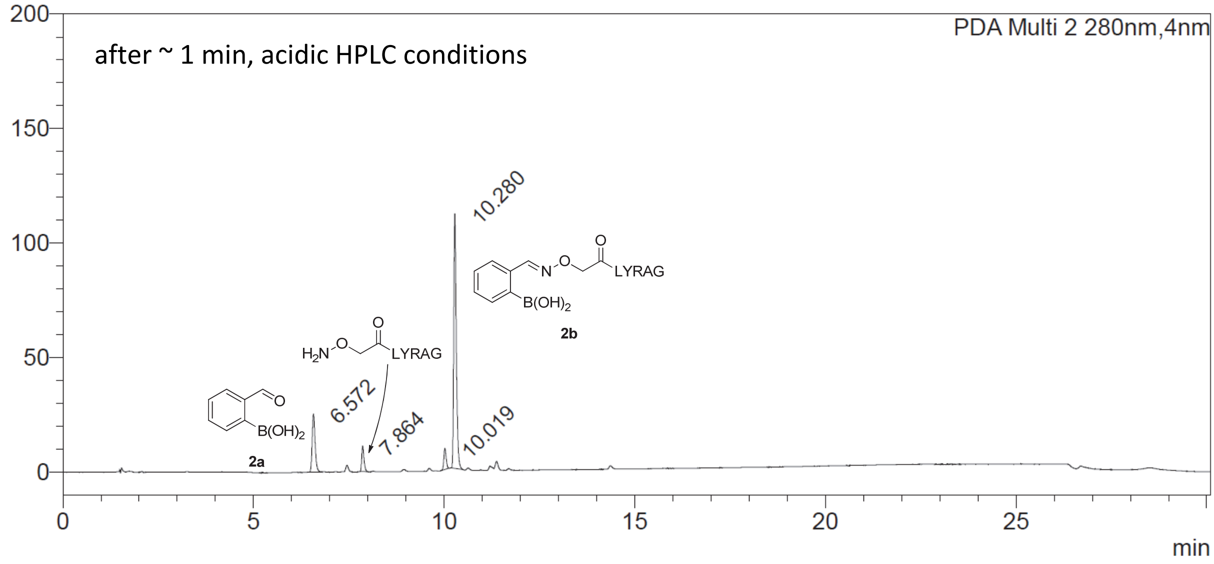

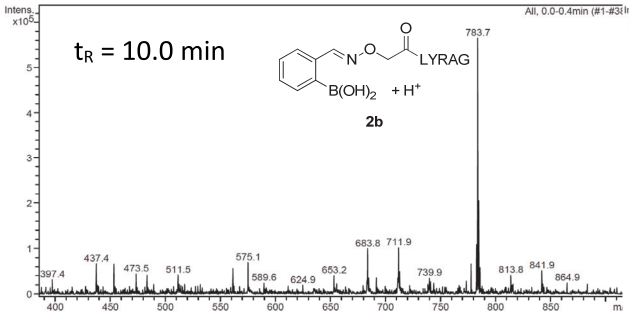

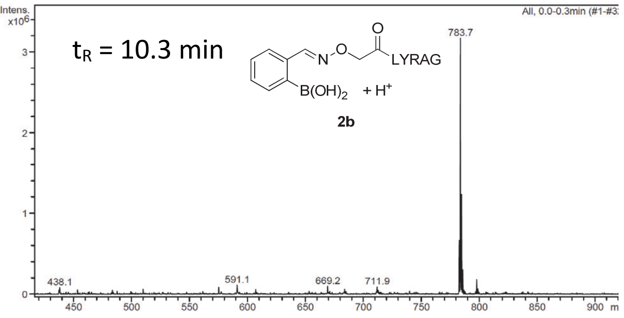

Table S1 Entry 4│ **Top:** HPLC trace at 254 nm after ~1 min – full conversion of 2-FPBA is achieved but the pentapeptide oxime **2b** cannot be detected under neutral HPLC conditions at 254 and 280 nm; **Middle:** HPLC trace at 280 nm after ~1 min under acidic conditions (eluent additive of 0.1% TFA) – 65% conversion is achieved of both the boronic acid (t_R = 6.5 min) and the pentapeptide

hydroxylamine (t_R = 7.8 min). It is noteworthy to mention that acidic HPLC conditions were found to compete in oxime hydrolysis and even after hours starting material peaks were still present which was never observed under neutral HPLC conditions. The desired pentapeptide oxime **2b** (t_R = 10.0 and 10.3 min) is assumed to be present as the *cis*- and *trans*-isomer since both peaks have the same mass. **Bottom:** ESI-MS of the peaks at 10.0 and 10.3 min suggesting the same mass of 783.7 for oxime **2b**.

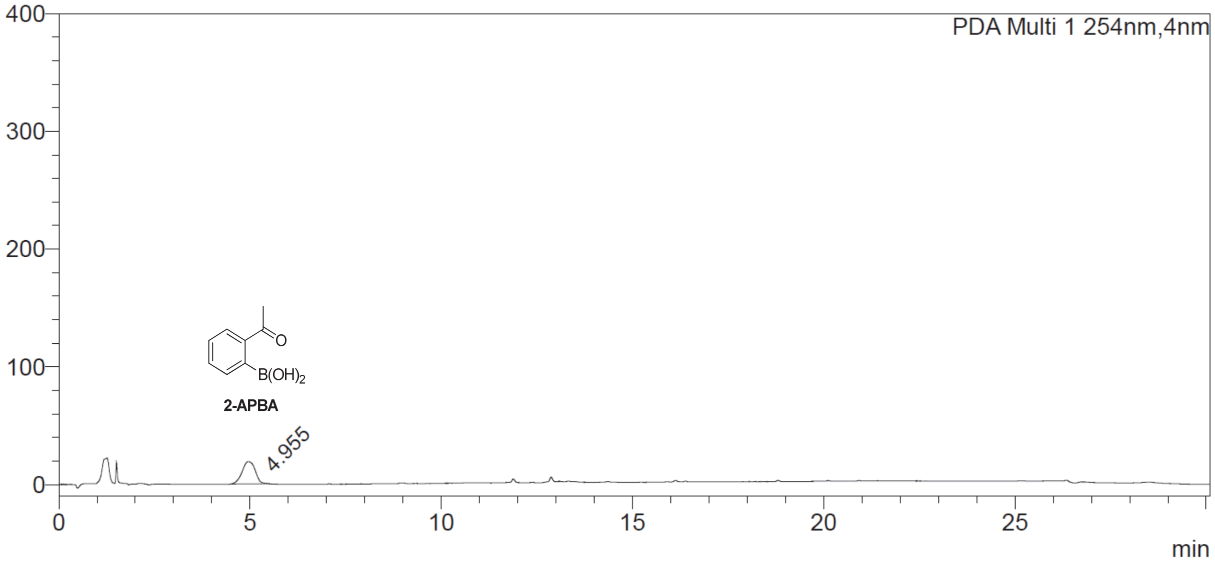

Figure S2: HPLC chromatogram of a 100 µM solution of 2-APBA.

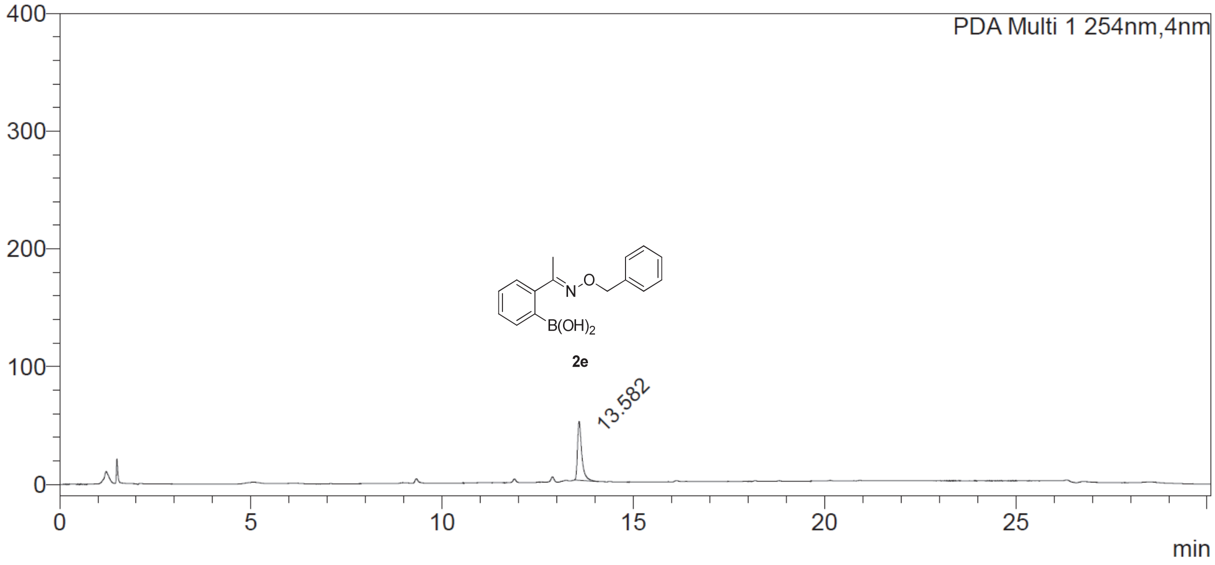

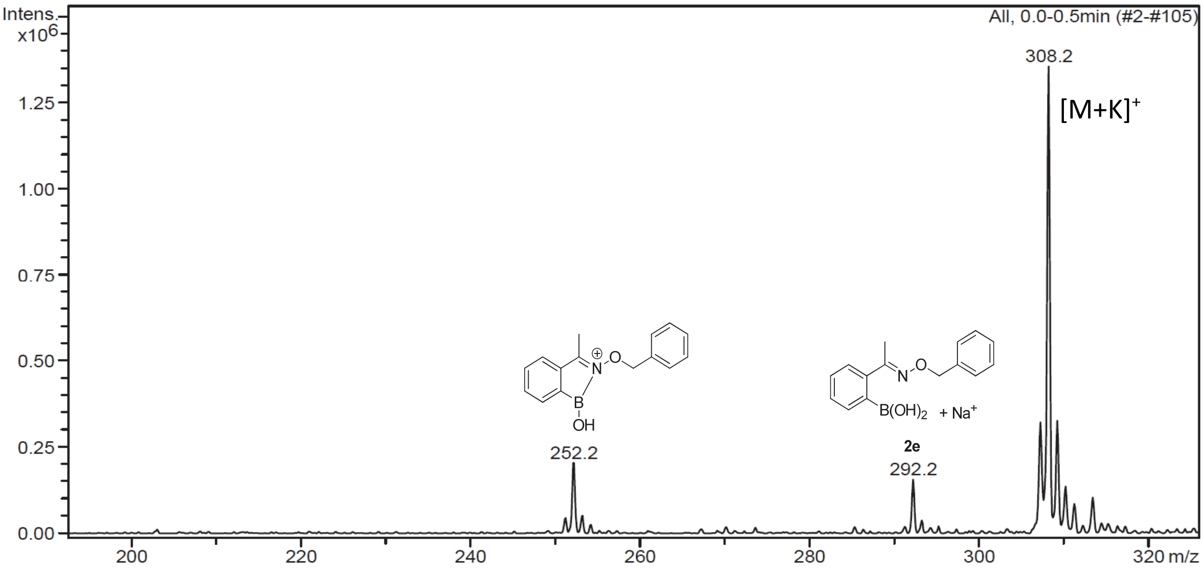

Table S1 Entry 7 **|** **Top:** HPLC trace at 254 nm after ~1 min – 94% conversion of 2-APBA is achieved along with the product peak of oxime **2e**; **Bottom:** ESI-MS spectra of the peak at t_R = 14.5 min. The major species observed is the potassium adduct and the charged oxime lacking a hydroxyl group.

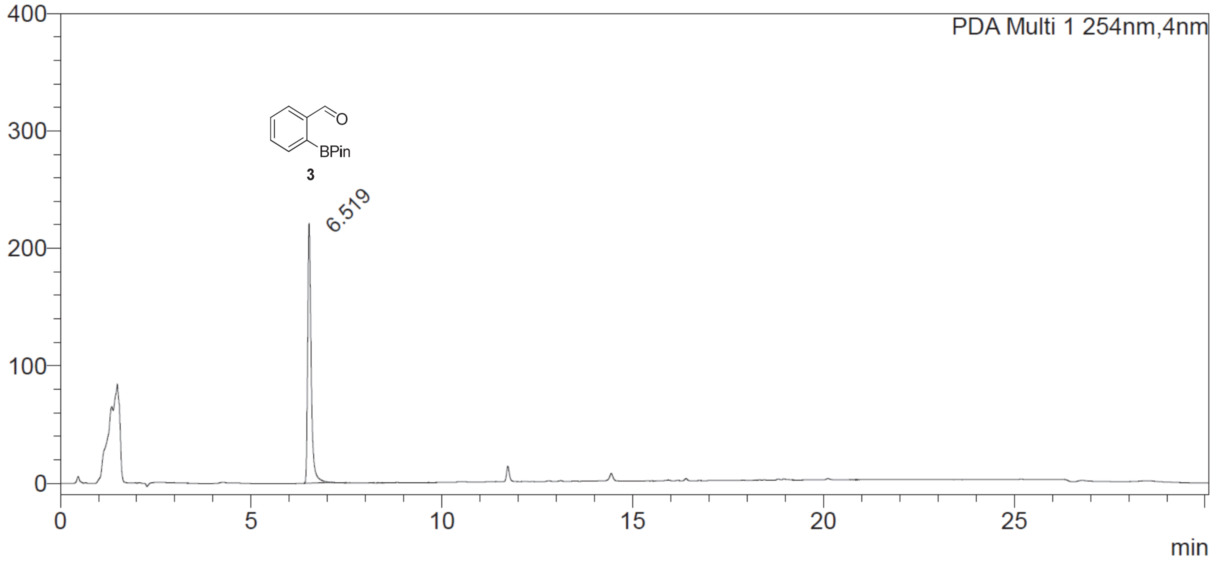

Figure S3: HPLC chromatogram of a 100 µM solution of **3** monitored at 254 nm.

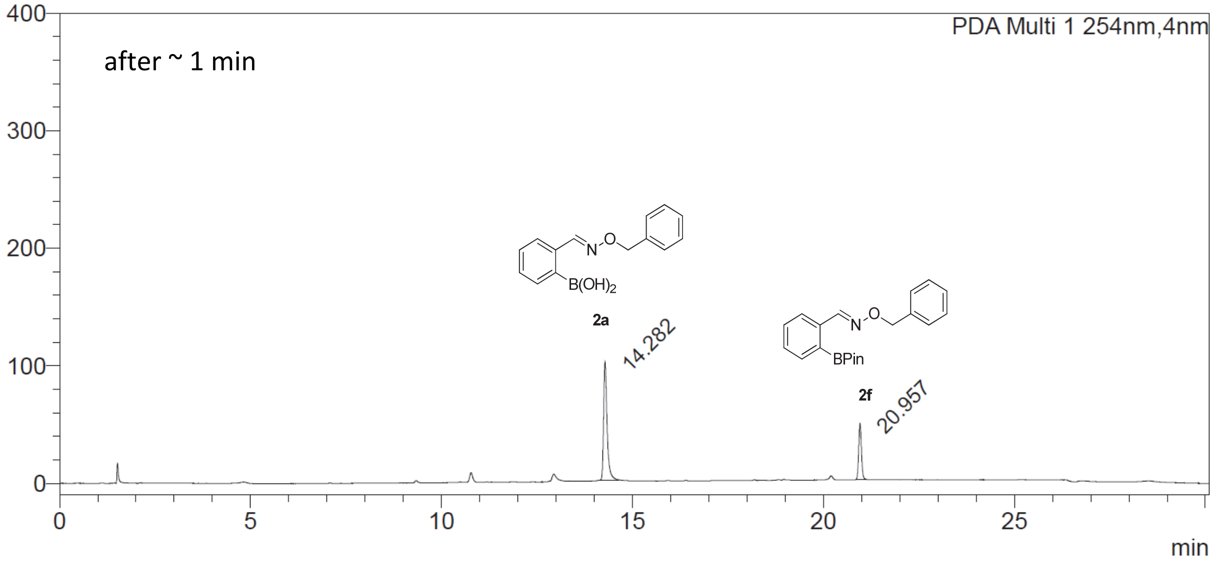

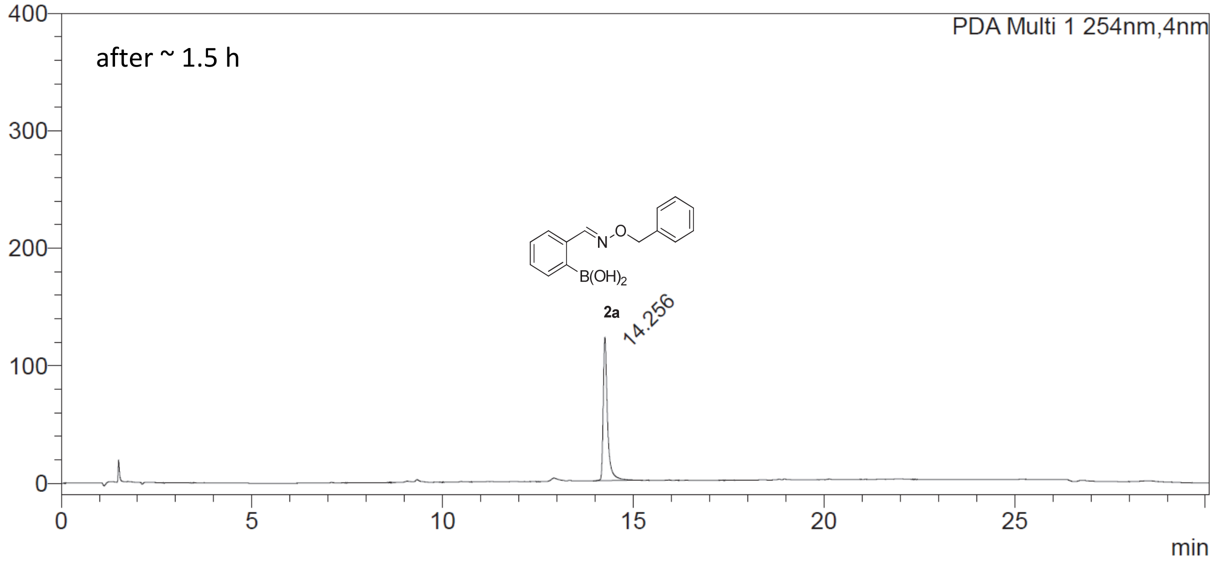

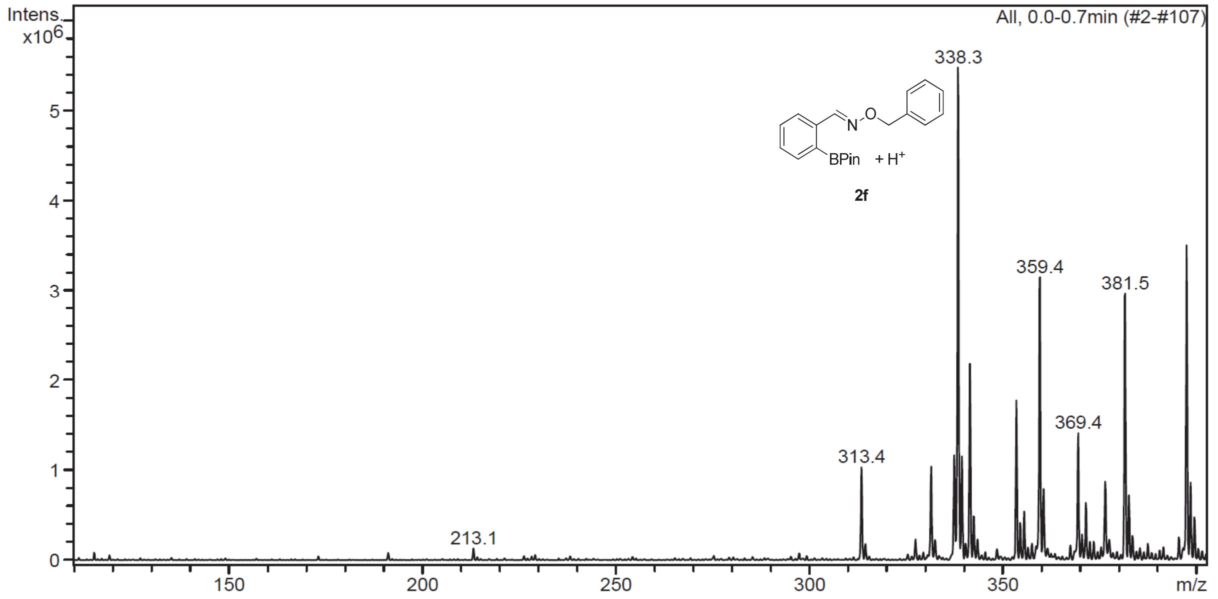

Table S1 Entry 8 | HPLC traces at 254 nm after ~1 min (**top**) and ~ 1.5 h (**middle**) – full conversion of **3** is achieved along with the product peaks of oxime **2f** and the hydrolyzed boronic acid oxime **2a**

which is the exclusive product after 1.5 h; **Bottom:** ESI-MS spectra of the peak at t_R = 20.9 min. The ESI-MS spectra of the peak at t_R = 14.3 min is already shown above for table entry 2.

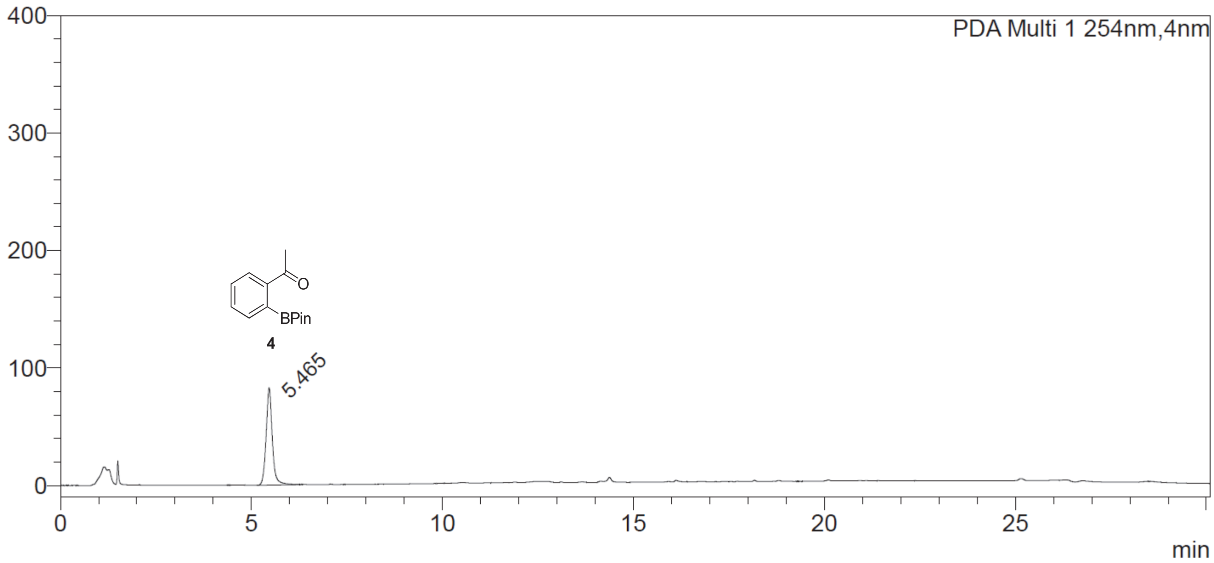

Figure S4: HPLC chromatogram of a 100 µM solution of **4** monitored at 254 nm.

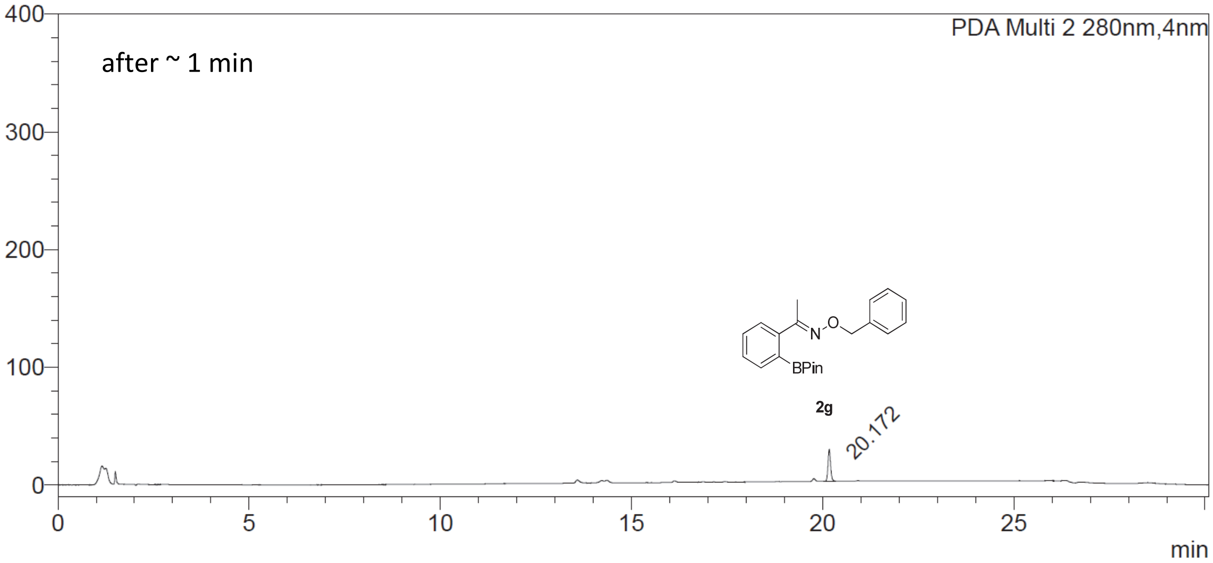

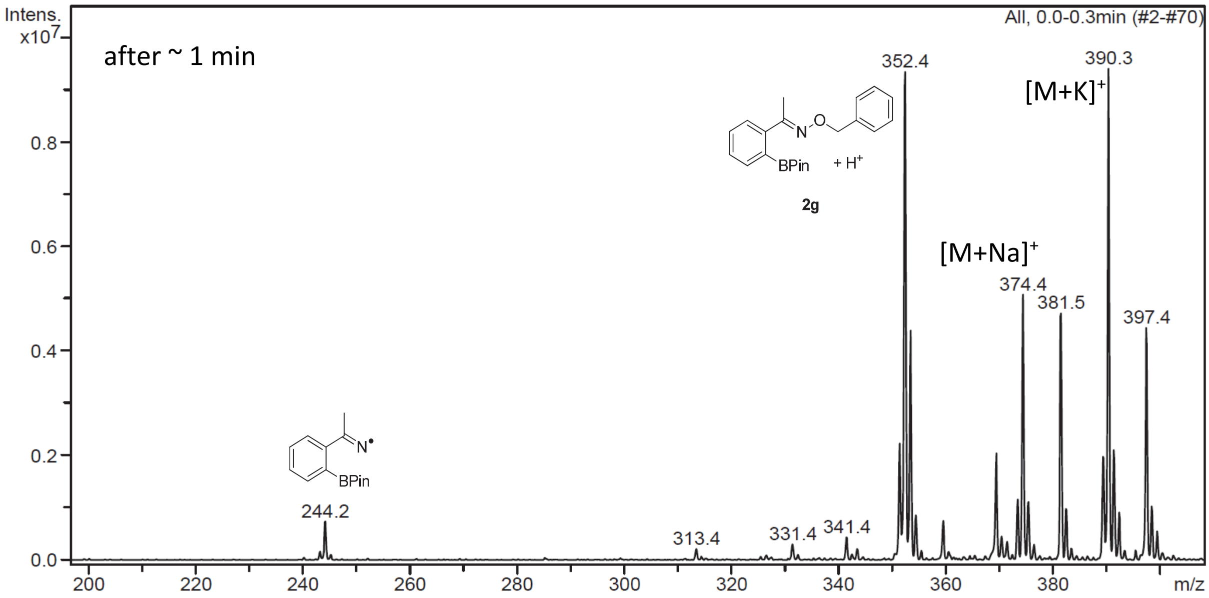
Intens.
x10^7
All, 0.0-0.3min (#2-#70)
after ~ 1 min
352.4
390.3
[M+K]+
BPin
+ H+
2g
[M+Na]+
374.4
381.5
397.4
BPin
244.2
313.4
331.4
341.4
m/z

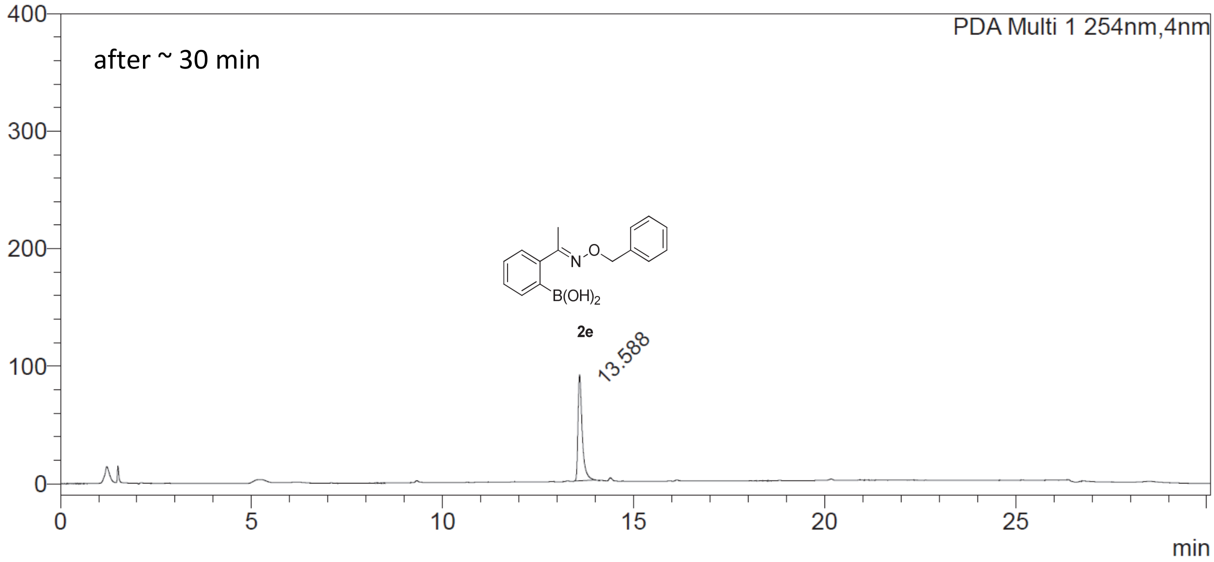
PDA Multi 1 254nm,4nm
after ~ 30 min
B(OH)2
2e
13.588
min

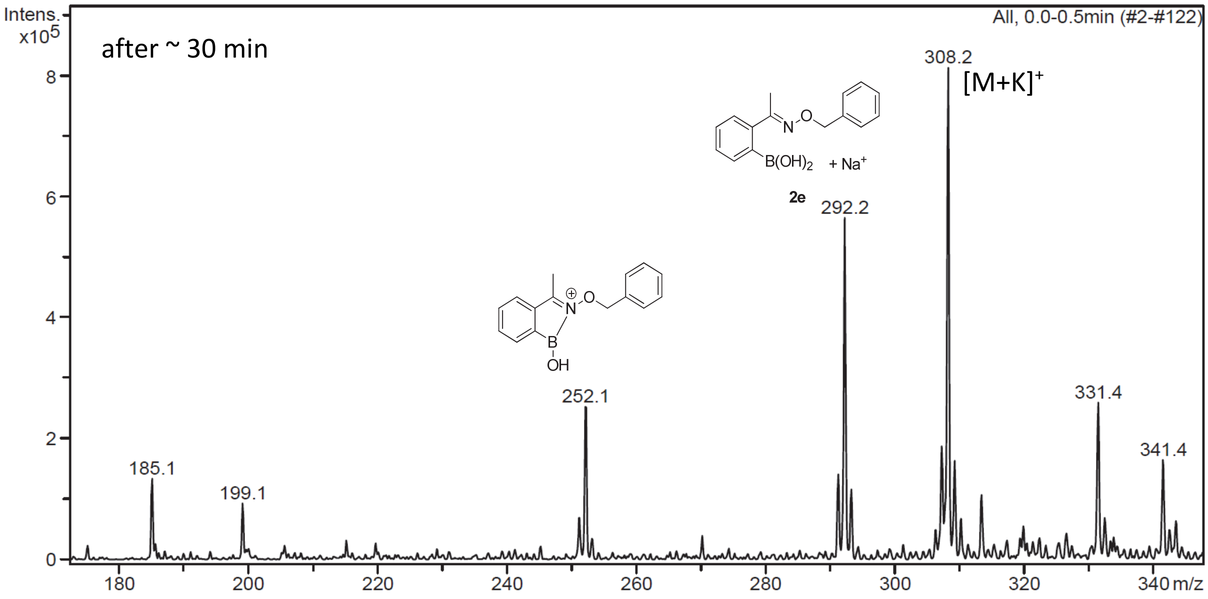
Intens.
x10^5
All, 0.0-0.5min (#2-#122)
after ~ 30 min
308.2
[M+K]+
B(OH)2
+ Na+
2e
292.2
OH
252.1
331.4
341.4
185.1
199.1
m/z

Table S1 Entry 9 | HPLC traces at 254 nm after ~1 min (**top**) and ~ 1.5 h (**bottom**) with their corresponding ESI-MS spectra – full conversion of **4** is achieved along with the product peaks of oxime **2g** which hydrolyzes in less than 30 minutes to give exclusively oxime **2e**.

4. Fluorescence Quenching Assay

4.1 General Information

The fluorescence quenching experiments were conducted using a TECAN Infinite M1000 PRO fluorescence plate reader and the data was analyzed with the TECAN i-control software. All experiments were performed using a Nunclon flat black 96-well Microplate from Thermo Scientific. The fluorescence was monitored at the maximum absorption of lissamine derivative **8** (566 nm) in potassium phosphate buffer (100 mM, pH 3.02, 3.89, 4.49, 5.00, 5.53, 6.14, 7.20 and 8.05). Stock solutions of the reaction partners in DMSO were freshly prepared and data was recorded the same day. The higher concentrated stock solutions were stored at -20°C and wrapped in aluminum foil to minimize bleaching. Prior to analysis all samples were gently allowed to warm up to room temperature in the absence of light and vortexed for 30 seconds to allow homogeneity.

4.2 Experimental setup

2 µL of a lissamine derivative **8** stock solution (50 µM or 10 µM, in DMSO) were added to a well filled with 196 µL of phosphate buffer solution (100 mM) with the respective pH followed by 2 µL of a dabsy derivative **7** stock solution (50 µM or 10 µM, in DMSO) to give a final volume of 200 µL (500 nM or 100 nM). The mixture was immediately mixed with a pipette to ensure homogeneity followed by monitoring the fluorescence at 566 nm. The control experiment was set up in analogy by adding 2 µL of a lissamine derivative **8** stock solution (50 µM or 10 µM, in DMSO) to a well filled with 196 µL of phosphate buffer solution (100 mM, pH = 5.00) followed by 2 µL of a dabsy derivative **7** stock solution (10 µM in DMSO). The mixture was immediately mixed with a pipette to ensure homogeneity followed by monitoring the fluorescence at 566 nm and 26°C. Both wells were analyzed simultaneously over a period of 7 minutes with data points recorded every 15 seconds. The time delay between mixing the samples and recording the first data point was determined to be 30 seconds.

4.3 Data processing

All experiments were triplicates and the data was processed in Microsoft Excel 2010 and plotted in OriginPro 8. The collected readout (fluorescence signal) was corrected by subtracting the control measurement with lissamine derivative **9** as well as for the 30 seconds delay time. The corrected readout was then converted into a concentration [Lissamine] and the triplicate average of 1/[Lissamine] in μM^{-1} was plotted against the corrected time points in seconds. Only data points which lie within the linear region of the plot were considered (435 seconds for 0.1 µM). All error bars represent the standard deviation of the respective data series and were determined with OriginPro 8. The value for k [M^{-1} s^{-1}] was determined as the slope of the linear fit of the respective graph.

4.4 Results

Attached to this supporting information is also an Excel table containing selected data in order to show the reader how the readout signals are processed to evaluate the numbers for *k* at the respective pH. This table contains also information on the following reactions:

500 nM, pH 7.20:

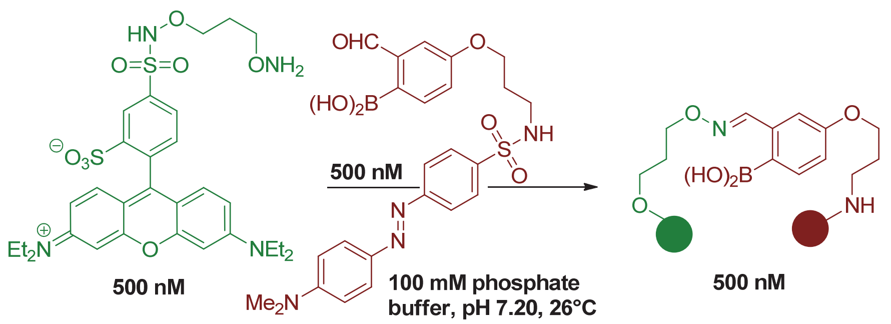

The reaction shows second order behavior only in the first 105 seconds and is too fast to get accurate kinetic data on the reaction at this concentration (Figure S5). The collected data is uncorrected for the time and the control measurement.

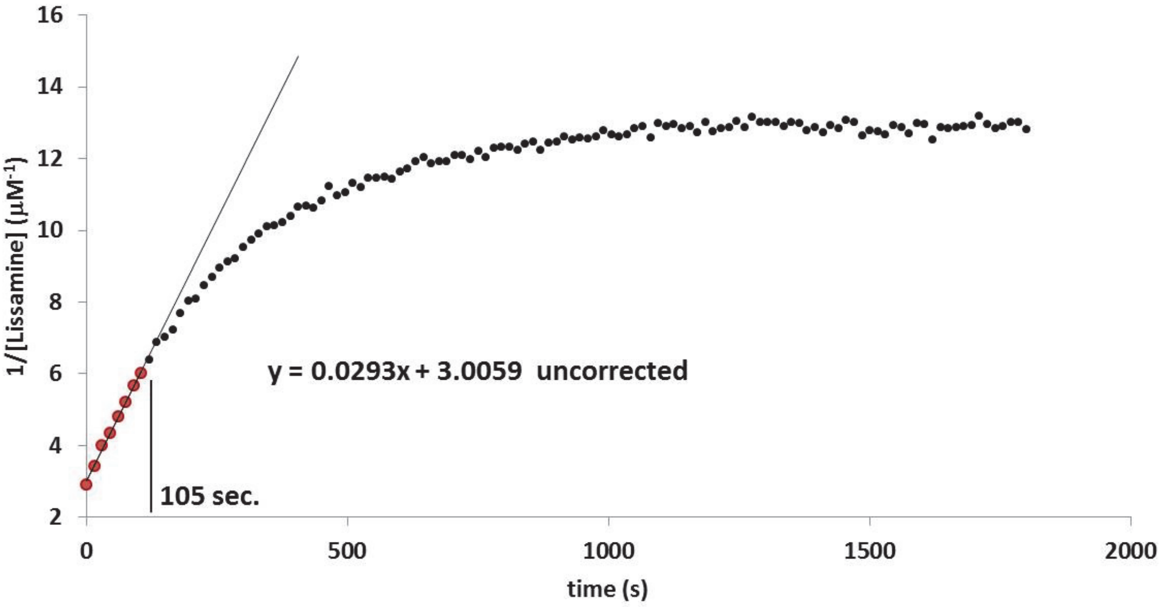

Figure S5: Fluorescence quench assay at 500 nM in 100 mM KP_i at pH 7.20 and 26°C. • Data points recorded over 30 min; • Data points within the linear region of the first 105 seconds fitted by a trend line.

Control reaction, 100 nM, pH 5.00:

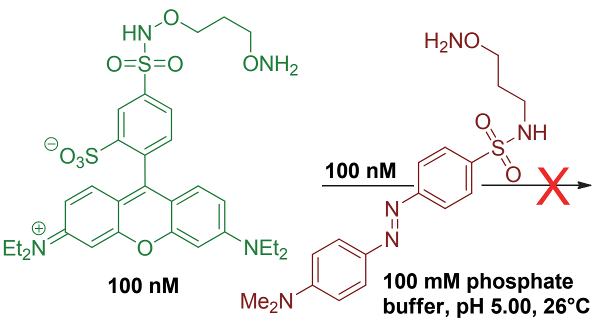

This control experiment was performed to get correction values (see additional Excel file - highlighted in green) which are a combination of self-bleaching and induced bleaching/quenching by the dabsyl moiety. The correction value at each time point is the difference of the fluorescence readout of the above control reaction and the fluorescence readout of the reaction at the respective concentration. All measurements are corrected by these values.

100 nM, pH 5.00, k = 14263 ± 405 M^{-1} s^{-1}:

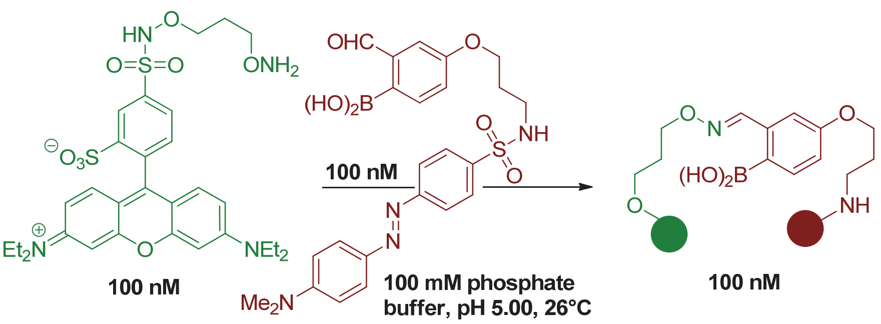

The reaction shows second order behavior up to 405 seconds and excellent linearity in inverse concentration plot (Figure S6). The collected data is corrected for the time and the control measurement.

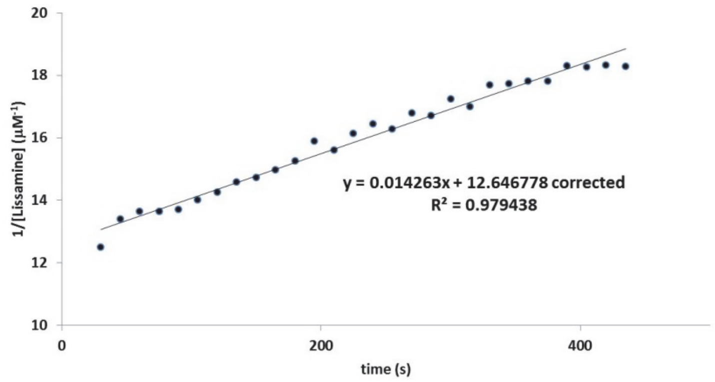

Figure S6: Fluorescence quench assay at 100 nM in 100 mM KP_i at pH 5.00 and 26°C. • Data points (corrected for time and the control measurement) recorded over 435 seconds are fitted by a trend line

(▬). The slope multiplied by 10^6 gives the rate constant k [M^{-1} s^{-1}] for this particular oxime condensation reaction at the respective pH.

All pH profile measurements were evaluated in Excel and plotted in OriginPro 8. All data presented is the average of triplicates and the error represents the standard error [M^{-1} s^{-1}]. Values for k [M^{-1} s^{-1}] represent the slope of the trend line which is obtained from the average data points collected in the first 405 seconds. Table S2 summarizes the results for the kinetic pH dependency of the oxime formation. These results are additionally plotted in a graph for better visualization (Figure S7).

Table S2: Results of the pH profile measurements at 100 nM. Values determined from triplicate measurements at 26°C.

pH	k [M^{-1} s^{-1}]	standard error [M^{-1} s^{-1}]
3.02	9386	± 325
3.89	11259	± 244
4.49	13839	± 328
5.00	14263	± 405
5.53	12161	± 377
6.14	11181	± 360
7.20	11113	± 284
8.05	10180	± 310

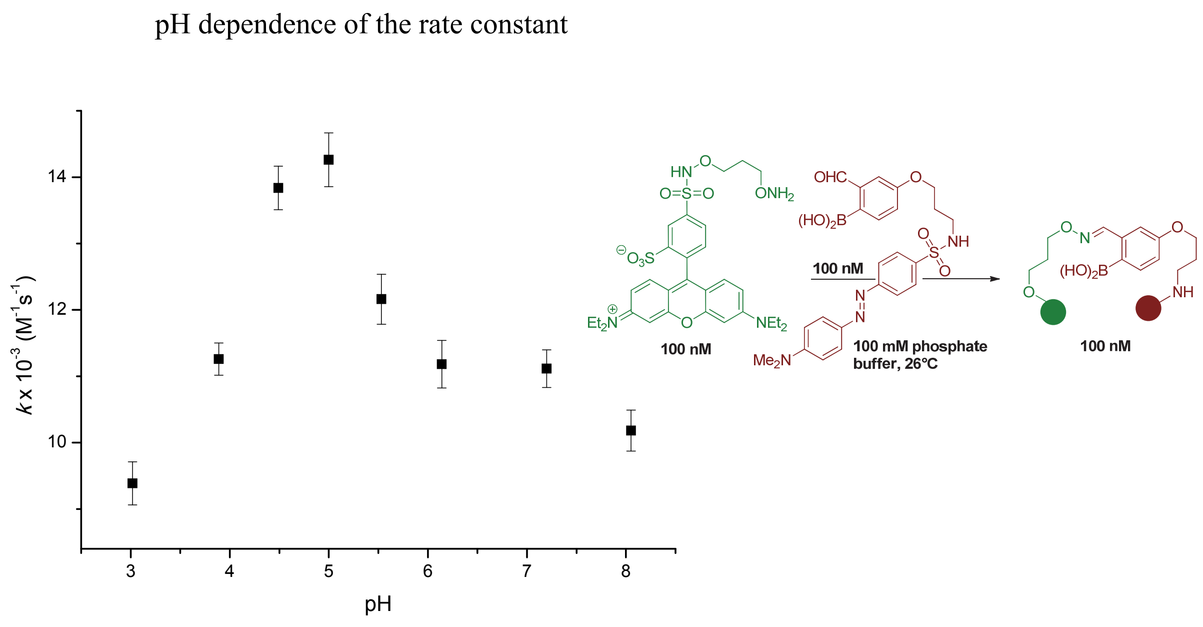

Figure S7: Rate constant k vs. pH plot of the oxime formation at 100 nM.

The following eight inverse concentration plots (pH 3.02 – 8.05) were generated in OriginPro 8 from processing raw data in Excel (see additional supporting Excel sheet for selected examples). Average data points ■ include standard error bars and a trend line (▬). The slope represents the rate constant [μM^{-1} s^{-1}] and is multiplied by a factor of 10^6 to obtain the final magnitude for k [M^{-1} s^{-1}]. The standard error in k is evaluated in the same manner.

pH 3.02

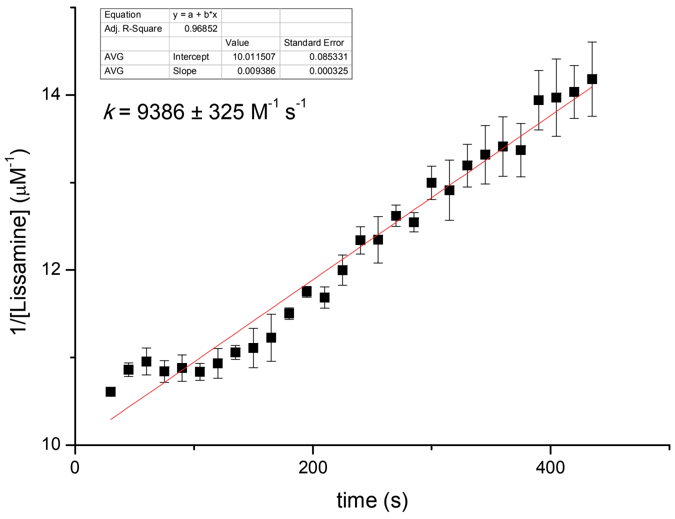

pH 3.89

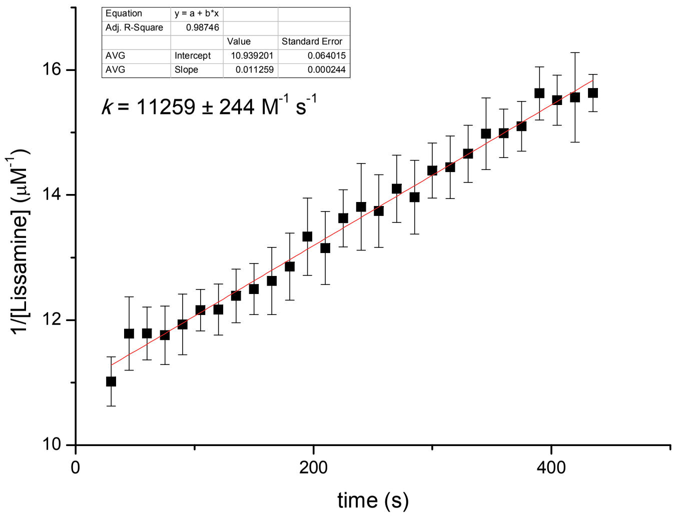

pH 4.49

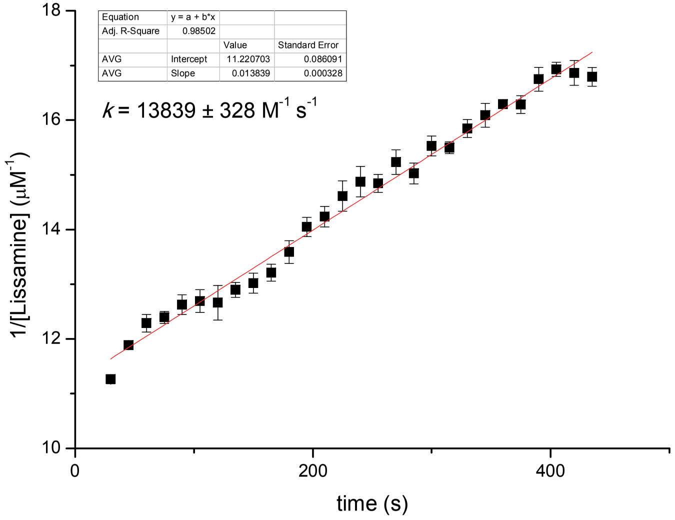

pH 5.00

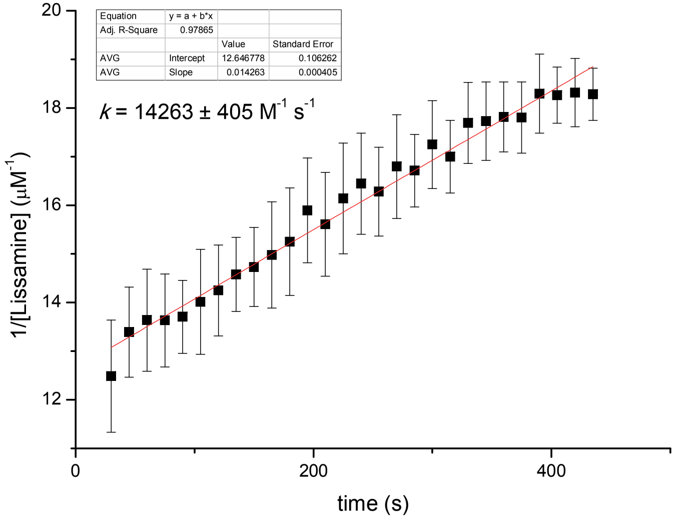

pH 5.53

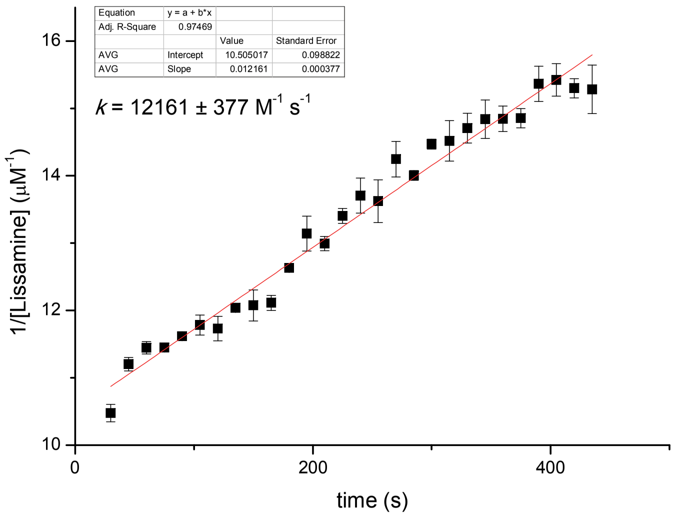

pH 6.14

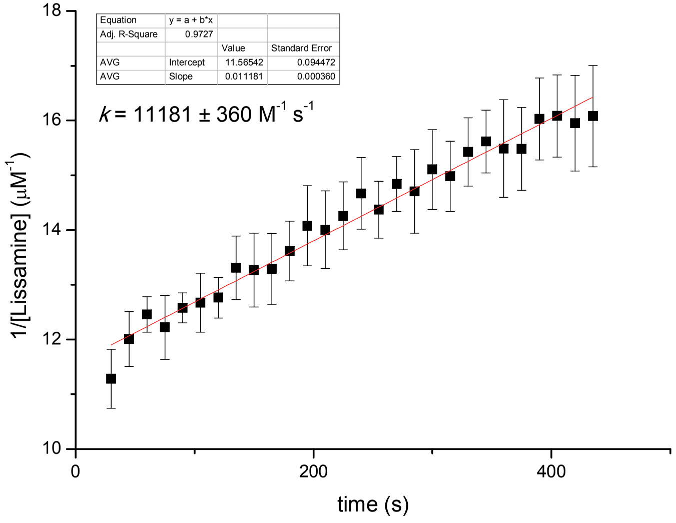

pH 7.20

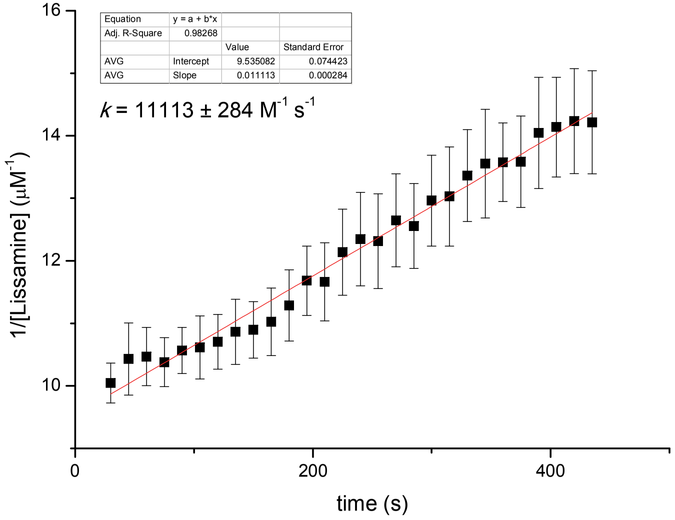

pH 8.05

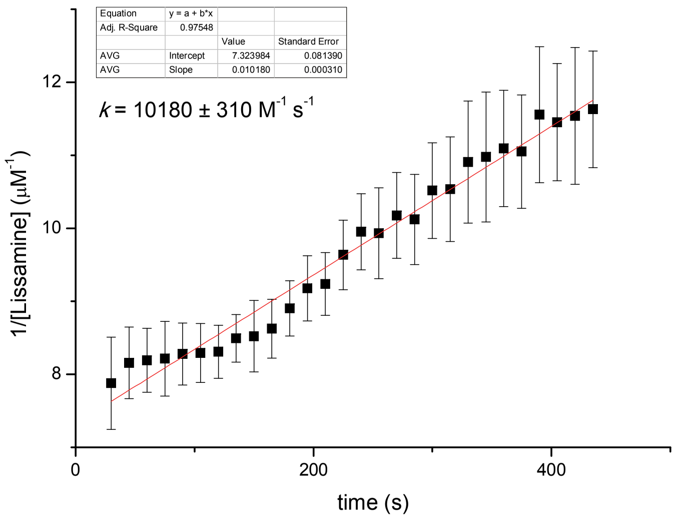

5. NMR stability and reversibility studies

5.1 General Information

The NMR experiments on the stability and reversibility of oxime **2a** were performed at 298 K on a BrukerAvance III NMR spectrometer operating at 600.13 MHz and equipped with a direct observe 5-mm BBFO smart probe. All 1D proton experiments were recorded using exactly the same parameters (256 scans, relaxation delay of 4.0 s and an acquisition time of 2.00 s) and were processed using TopSpin software from Bruker using line broadening of 0.5 Hz and after manual phase correction automated baseline correction was applied. Chemical shifts were referenced to the residual solvent peak of acetonitrile (1.94 ppm) and TMSP-d_4 (3-(trimethylsilyl)-2,2',3,3'-tetradeuteropropionic acid) was used as an internal standard. The pH (no correction for deuterium) was adjusted using a 827 pH Lab – Metrohm equipped with a glass minitrode which was pre-rinsed with D_2O.

5.2 ^{1}H-NMR stability test of oxime 2a

A stock solution of oxime **2a** was freshly prepared in CD_3CN. A K_3PO_4 buffer stock solution was freshly prepared in D_2O adjusting the pH to 7.20 with concentrated DCl from ABCR (DCl, 38% wt% in D_2O, 99.5% atom%D). A TMSP-d_4 stock solution was freshly prepared in D_2O. All experiments were conducted at 298 K in standard NMR tubes (throw away quality) which were rinsed thoroughly with D_2O and oven dried prior to use.

Setup for the ^{1}H-NMR stability experiment at 100 μM:

1. 430 μL of phosphate buffer (12.8 mM) were mixed inside the NMR tube with 110 μL of oxime **2a** (500 μM) and 10 μL of TMSP-d_4 (275 μM). The sample was then locked to acetonitrile and shimmed before recording the first ^{1}H-NMR spectrum.

The final volume of the NMR sample was 550 μL with the following final concentrations:

100 μM of oxime **2a**, 10 mM phosphate buffer and 5 μM TMSP-d_4 with a ratio of 4:1 (10 mM KP_i in D_2O/CD_3CN).

The stability of oxime **2a** was followed by recording ^{1}H-NMR spectra every 7 h for the first day and then one spectrum every day for another two days. The integration of the oxime proton (blue) was compared to the internal standard (pink) integral at 1, 24, 48 and 72 h (Figure S8).

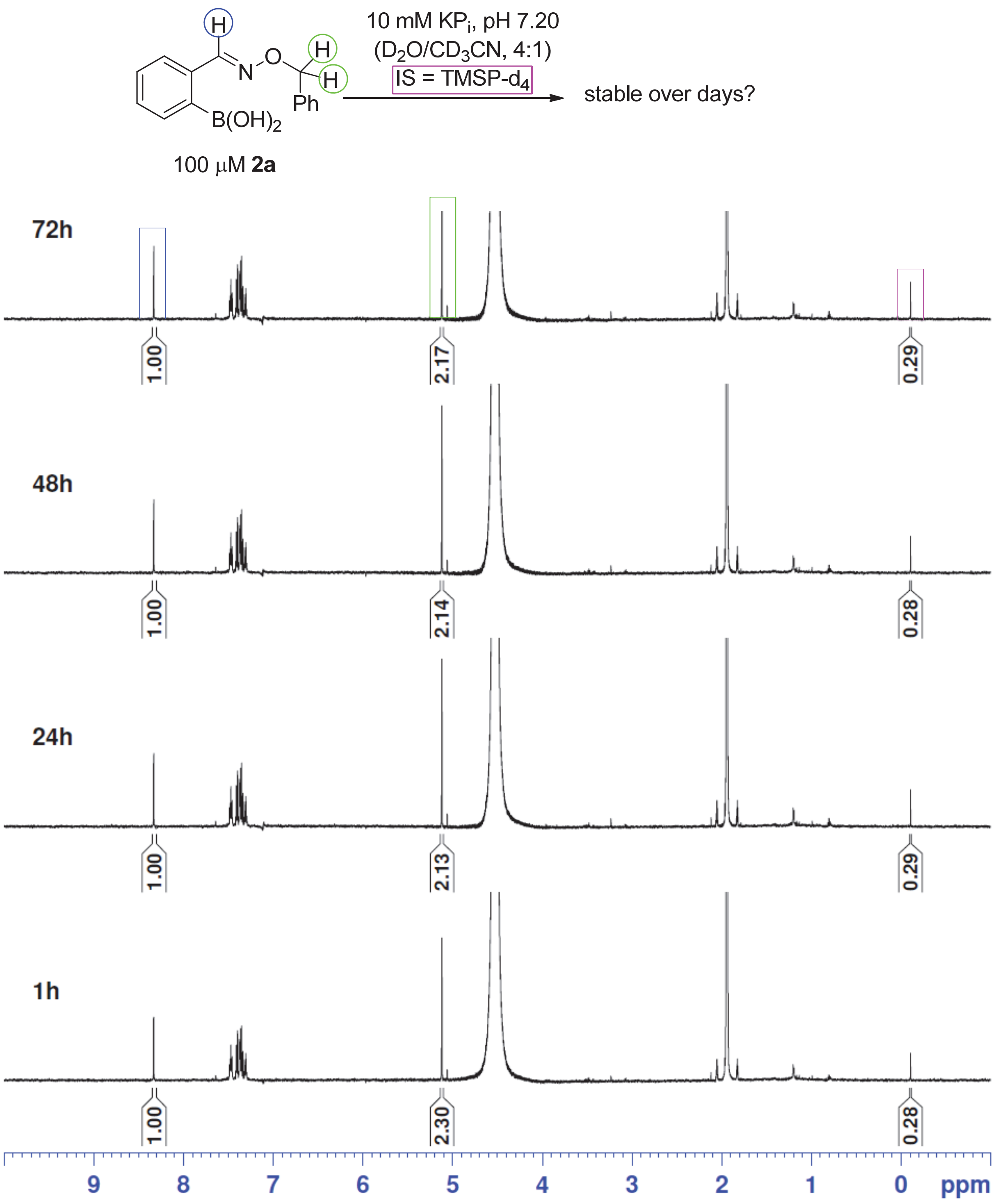

Figure S8: ^{1}H-NMR stability test of oxime **2a** over the course of 72 h at pH 7.20 and 100 µM. The change in concentration over the course of time was determined to be <5% which lies within the accuracy of integration at 100 µM.

5.3 ^{1}H-NMR reversibility assay of oxime 2a

Stock solutions of oxime **2a** (in CD_3CN) and Methoxyamine hydrochloride (Supelco) in D_2O were freshly prepared the day they were used. A K_3PO_4 buffer stock solution was freshly prepared in D_2O adjusting the pH to 7.20 with concentrated DCl from ABCR (DCl, 38% wt% in D_2O, 99.5% atom%D). A TMSP-d_4 stock solution was freshly prepared in D_2O. All experiments were conducted at 298 K in standard NMR tubes (throw away quality) which were rinsed thoroughly with D_2O and oven dried prior to use.

Setup for the ^{1}H-NMR reversibility experiment at 100 μM:

1. 420 μL of phosphate buffer (13.1 mM) were mixed inside the NMR tube with 110 μL of oxime **2a** (500 μM), 10 μL of TMSP-d_4 (275 μM) and 10 μL of Methoxyamine hydrochloride (27.5 mM). The sample was then sonicated, locked to acetonitrile and shimmed before recording the first ^{1}H-NMR spectrum.

The final volume of the NMR sample was 550 μL with the following final concentrations:

100 μM of oxime **2a**, 10 mM phosphate buffer, 5 μM TMSP-d_4 and 500 μM of Methoxyamine hydrochloride with a ratio of 4:1 (10 mM KP_i in D_2O/CD_3CN).

The reversibility of oxime **2a** was followed by recording ^{1}H-NMR spectra every 2 h for a total of 30 measurements (60 h) tracing the oxime protons **H_a** and **H_b** (Scheme S2). A stack of selected ^{1}H-NMR spectra is presented in Figure S9a and shows the process of oxime interconversion which reaches equilibrium between 9 and 19 h. The final ratio of the newly formed oxime **11** and oxime **2a** was found to be 3:1.

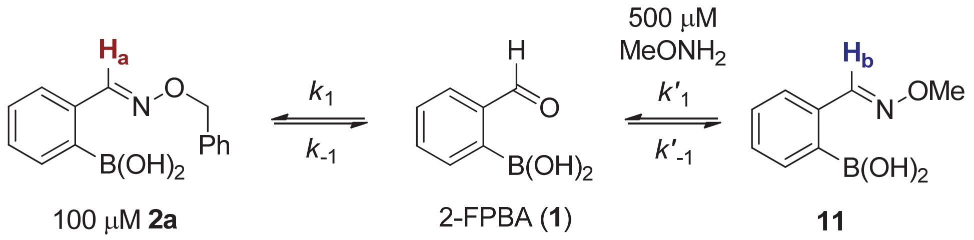

^{1}H-NMR conditions: 10 mM KPi, pH 7.20 (D_2O/CD_3CN, 4:1), IS = TMSP-d_4, 298 K, 60 h.

Scheme S2: Qualitative reversibility scheme of oxime **2a** which is treated with a fivefold excess of methoxyamine. The respective oxime protons **H_a** and **H_b** are highlighted for clear differentiation.

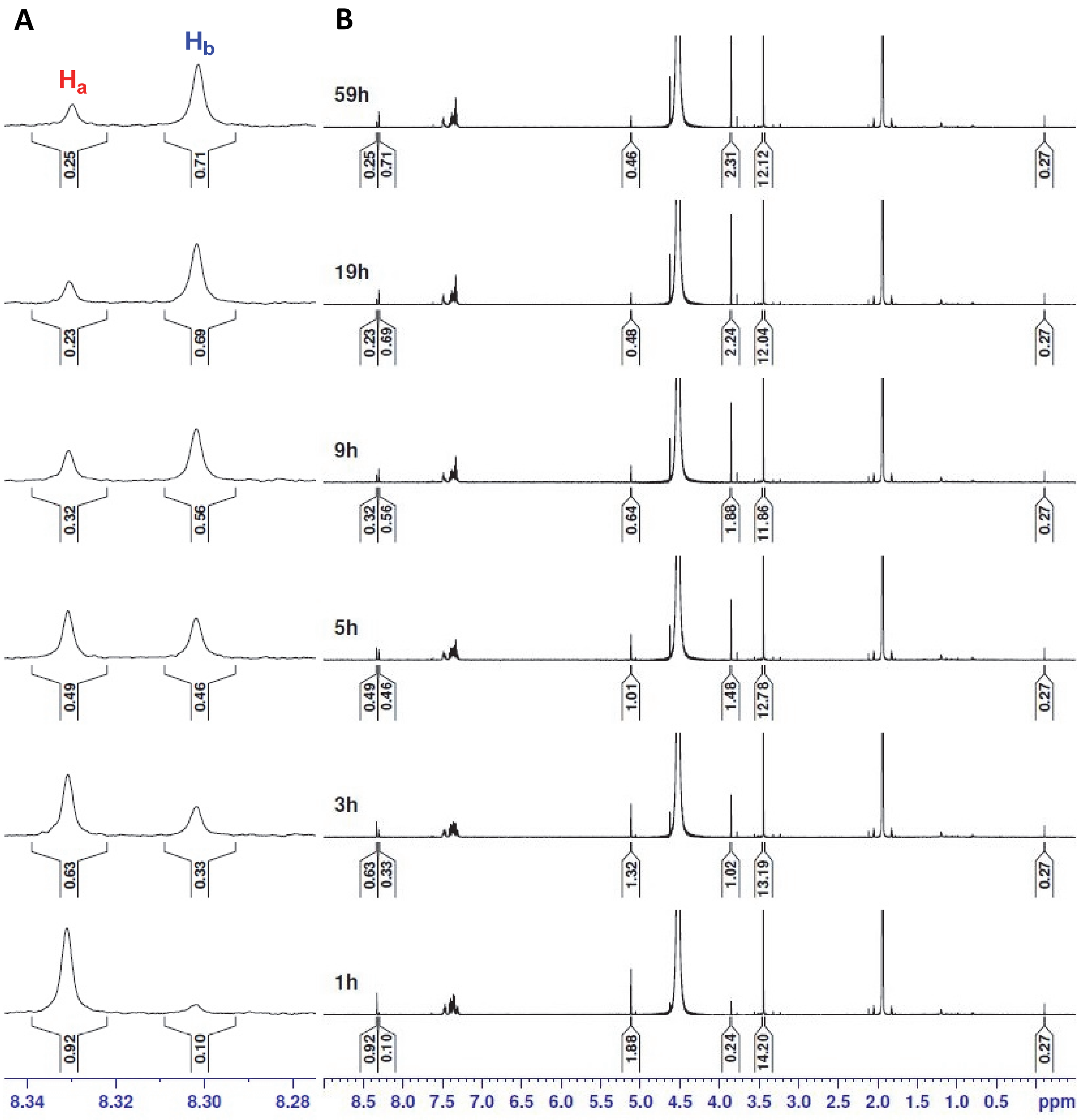

Figure S9a: ^{1}H-NMR reversibility test of oxime **2a** and methoxyamine. Only six selected time points are shown from a total of 30 measurements: **A**) zoomed region (8.28 - 8.34 ppm) of the oxime protons **H$_a$** and **H$_b$**. **B**) full ^{1}H-NMR spectrum of the reaction mixture at different time points containing integration values (up- to downfield) for the internal standard, methyl group of methoxyamine, methyl group of the newly formed oxime **11**, benzyl protons of **2a** and the two different oxime protons **H$_a$** and **H$_b$**.

The above proposed mechanism of oxime equilibration was confirmed separately in an additional ^{1}H-NMR experiment. Oxime **2a** was treated with a fivefold excess of aldehyde **12**[4] under neutral conditions using the experimental setup described above (scheme S3).

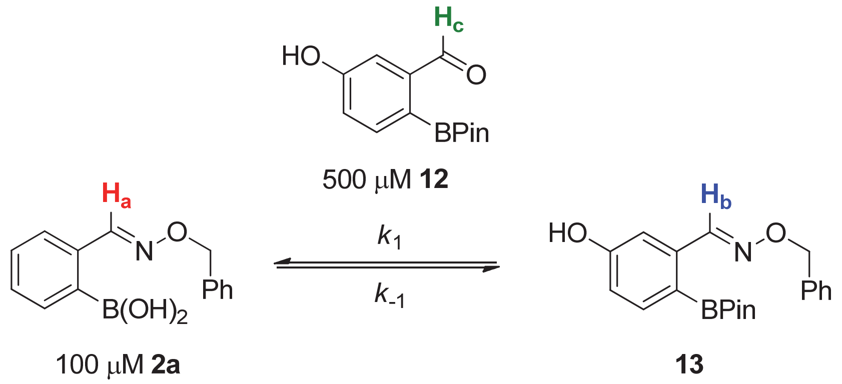

^{1}H-NMR conditions: 10 mM KPi, pH 7.20 (D_2O/CD_3CN, 4:1), IS = TMSP-d_4, 298 K, 24 h.

Scheme S3: Qualitative reversibility scheme of oxime **2a** which is treated with a fivefold excess of aldehyde **12**. The respective oxime protons $\mathbf{H_a}$, $\mathbf{H_b}$ and $\mathbf{H_c}$ are highlighted for clear differentiation.

A stack of selected ^{1}H-NMR spectra is presented in Figure S9b and shows the process of oxime interconversion from **2a** to **13** over 24 h. The data clearly indicates the exchange of reaction partner which is only possible through initial oxime hydrolysis of **2a** liberating O-benzylhydroxylamine which then undergoes another oxime condensation with **12** to give oxime **13** as the major species after 24 h. This reaction pathway shows that the oxime equilibration takes place by oxime hydrolysis and subsequent condensation rather than a direct addition of the hydroxylamine to the oxime as already indicated in scheme S2.

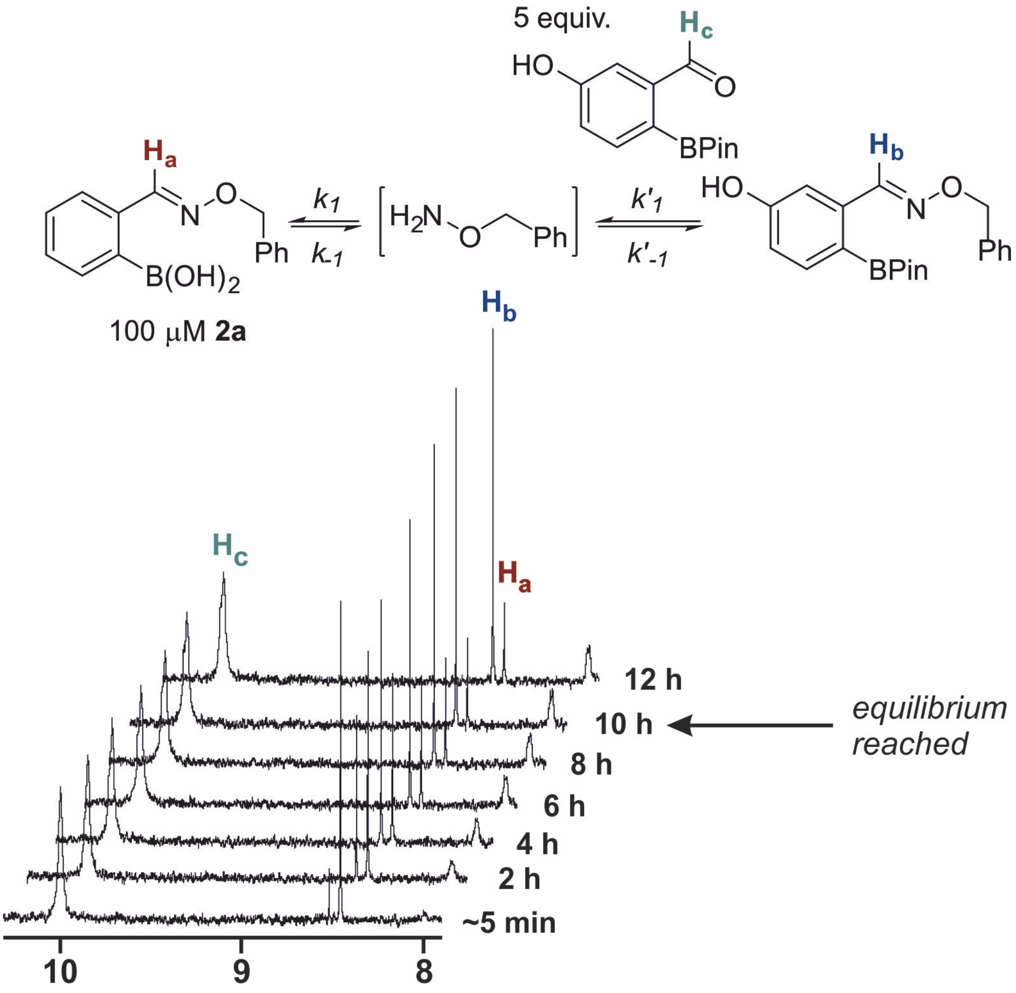

Figure S9b: Time dependent ^{1}H-NMR interconversion of oxime **2a** to oxime **13** using a fivefold excess of aldehyde **12**. The ^{1}H-NMR spectrum shows the zoomed regions (7.8 - 10.3 ppm) of the aldehyde H_c and oxime protons H_a and H_b. The equilibrium is reached after 10 h where the final ratio of **13** to **2a** is 10:1.

The integral values were converted into a concentration and a first order plot for the reverse reaction was done to determine k_{-1} (Figure S10).

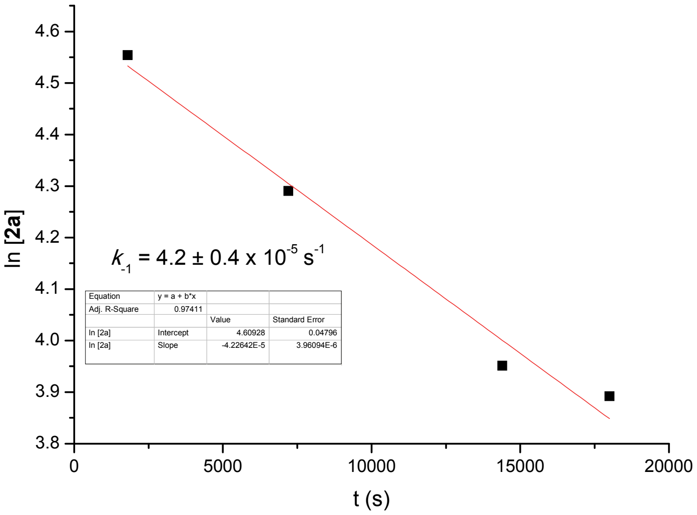

Figure S10: Plot of ln[**2a**] (determined from the oxime proton **H_a** integrals) vs. time. To evaluate the rate constant k_{-1} for the reverse reaction only time points between 0.5 and 5 h were considered.

The slope of the trend line (—) represents the rate constant k_{-1} for the reverse reaction of **2a**. Its value of $4.2 \pm 0.4 \times 10^{-5}$ s^{-1} is obtained directly from the graph along with the standard error. Using equations 1 and 2 the equilibrium constant K_{eq} and its uncertainty can be calculated inserting the following values:

$$k_1 = 11113 \pm 284 \text{ M}^{-1} \text{ s}^{-1}$$

$$k_{-1} = 4.2 \pm 0.4 \times 10^{-5} \text{ s}^{-1}$$

$$K_{eq} = \frac{k_1}{k_{-1}} \qquad (1)$$

$$\Delta K_{eq} = K_{eq} \times \sqrt{\left(\frac{\Delta k_1}{k_1}\right)^2 + \left(\frac{\Delta k_{-1}}{k_{-1}}\right)^2} \qquad (2)$$

This gives an equilibrium constant $K_{eq} = 2.6 \pm 0.3 \times 10^8$ M^{-1} for this particular type of oxime formation.

6. Boron-assisted ligation in complex environments

6.1 General Information

Stock solutions of 2-FPBA and O-benzylhydroxylamine were prepared in HPLC grade MeCN. Stock solutions of L-glutathione (reduced, Sigma Aldrich), sucrose (Fluka) and lysozyme (hen eggwhite, Roth) were prepared in nanopure water. The lysozyme concentration was determined using a NanoDrop 2000 spectrophotometer from Thermo Scientific with a path length of 1 mm and $\varepsilon_{(\text{lysozyme oxidized cystein 280 nm})} = 37970\ M^{-1}\ cm^{-1}$. Human Serum (male) type AB was purchased from Sigma Aldrich and stored at -20°C. The different stock solutions for condensation were allowed to warm to room temperature, vortexed and centrifuged prior to mixing and combined in the following order in an PP HPLC vial (BGB, Part Number: PPSV0903K): 1) 100 mM potassium phosphate buffer pH = 7.2, 2) hydroxylamine, 3) interfering additive 4) 2-FPBA. The resulting solution was briefly mixed with a Gilson Pipetman before injection (time between mixing and injection is around 30 seconds). The reactions (Table S3 entries 1-5) were followed by RP-HPLC on a Shimadzu Prominence UFLC Liquid Chromatograph using a C18 column (Eclipse XDB, 5 µm, 4.6 x 150 mm) from Agilent (gradient: 2-98% MeCN containing 1% H_2O in H_2O containing 1% MeCN over 18 min, flow rate 1 mL/min, room temperature, neutral conditions) monitoring and collecting the products at 254 nm or 280 nm if not otherwise specified.

Table S3: Tolerance of the condensation to biological interfering agents.

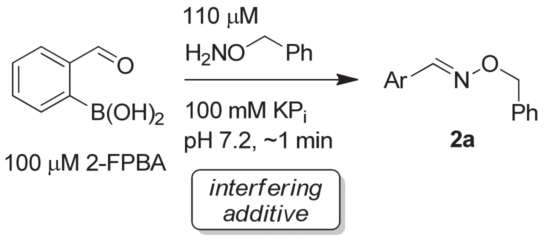

Entry	Additive (conc)	Normalized **2a** integral (%)	significance
1	None	100	-
2	Glutathione (5 mM)	98	Biothiols do not interfere
3	Sucrose (100 µM & 5 mM)	106/92	Boron chelators do not interfere
4	Lysozyme (100 µM)	105	Amino acid side-chains cannot compete with O-alkylhydroxylamine for 2-FPBA
5	Human serum (20% v/v)	80	Reaction is compatible with complex media

6.2 Corresponding UPLC/HPLC traces and ESI-MS spectra

The corresponding ESI-MS spectra for oxime **2a** were already shown in section **3.2** and can be looked up.

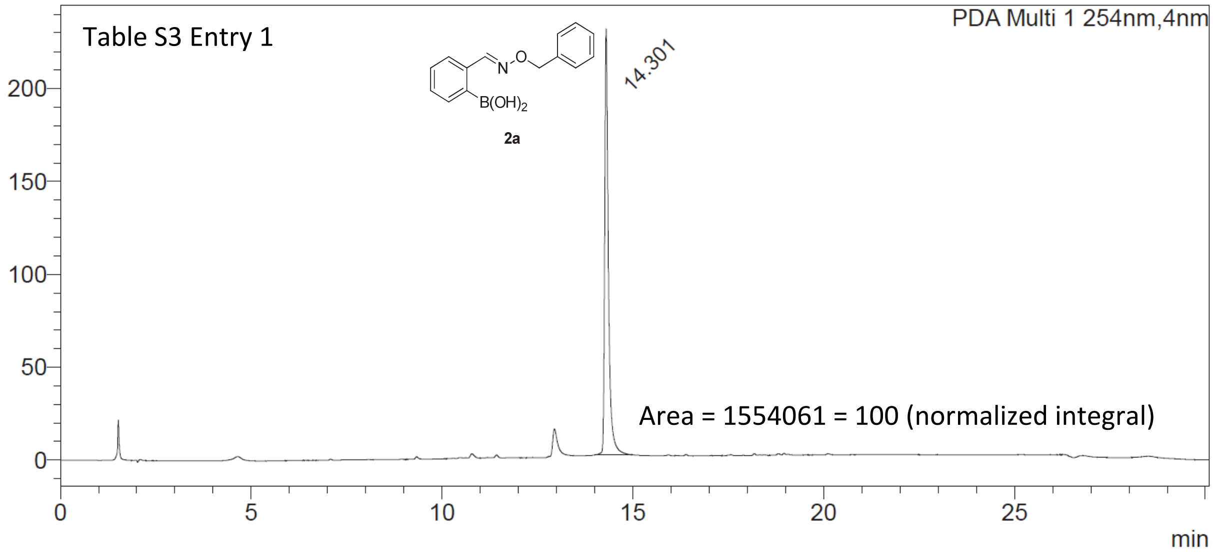

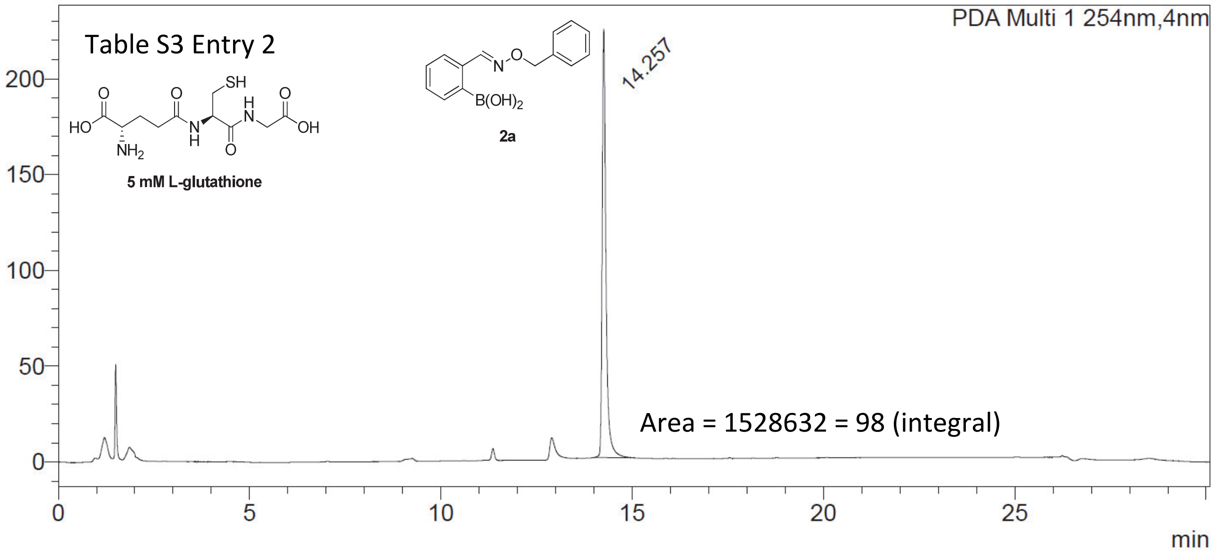

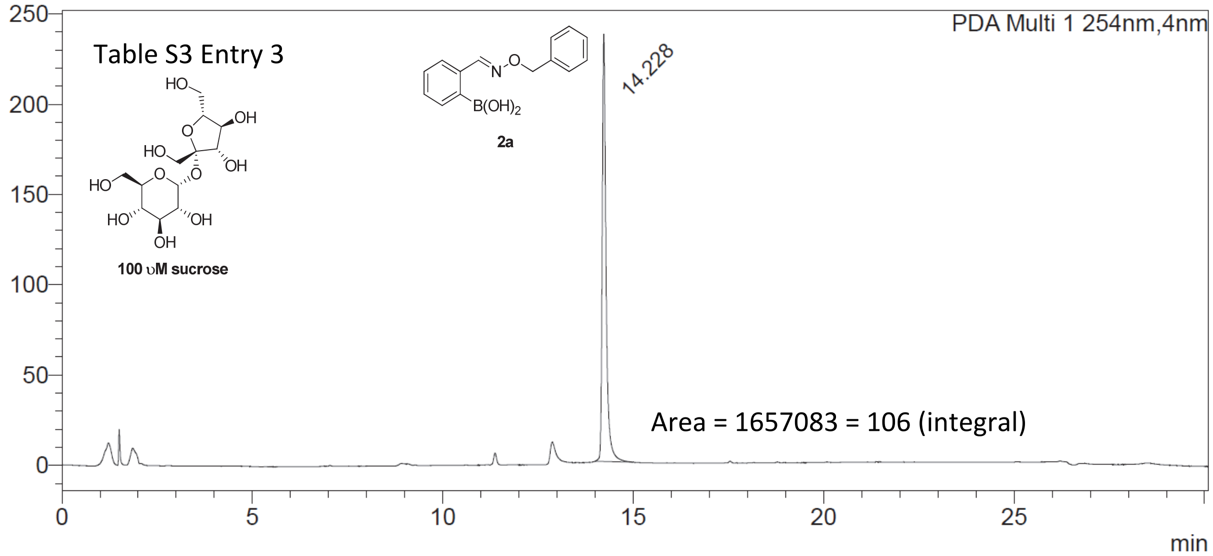

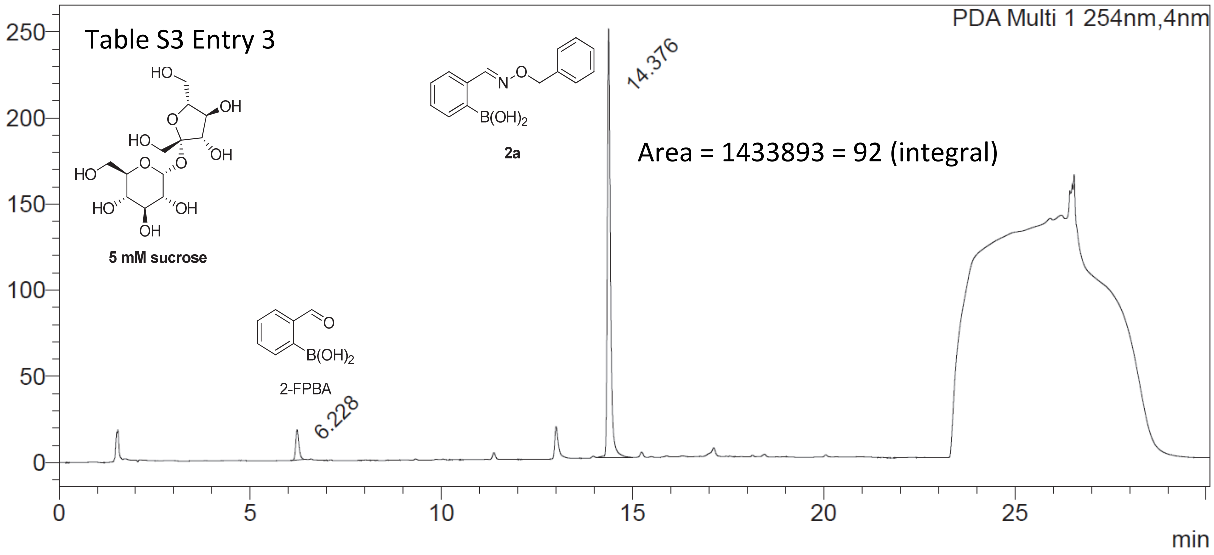

When employing sucrose at 5 mM it was observed that 5% of 2-FPBA was still present after one minute but the oxime **2a** was formed with a normalized integral of 92 as the exclusive product. The high concentration of sucrose used led probably to elution of accumulated compounds on the column which was observed as a big and broad peak between 23-29 minutes. This reaction was also performed by pre-mixing the boronic acid with the sucrose, with no measurable change in conjugation efficiency.

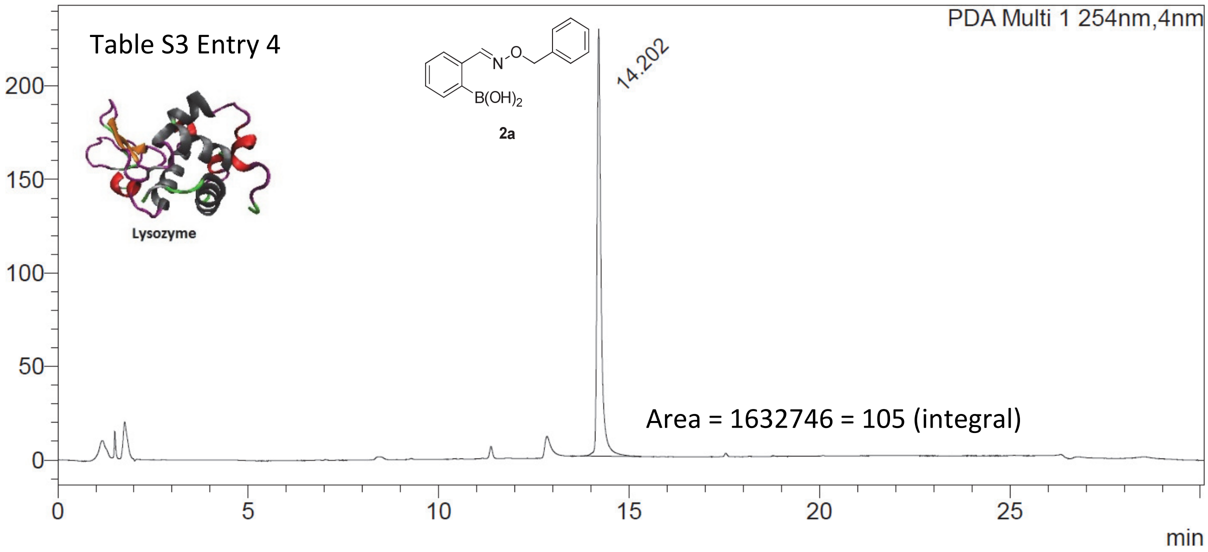

The reaction with interfering lysozyme additive was also analyzed by UPLC-MS under slightly acidic conditions (0.1% formic acid additive in the elution buffer) which showed no protein modification even after 12 h and complete conversion to the oxime **2a** (see Figures S11 and S12).

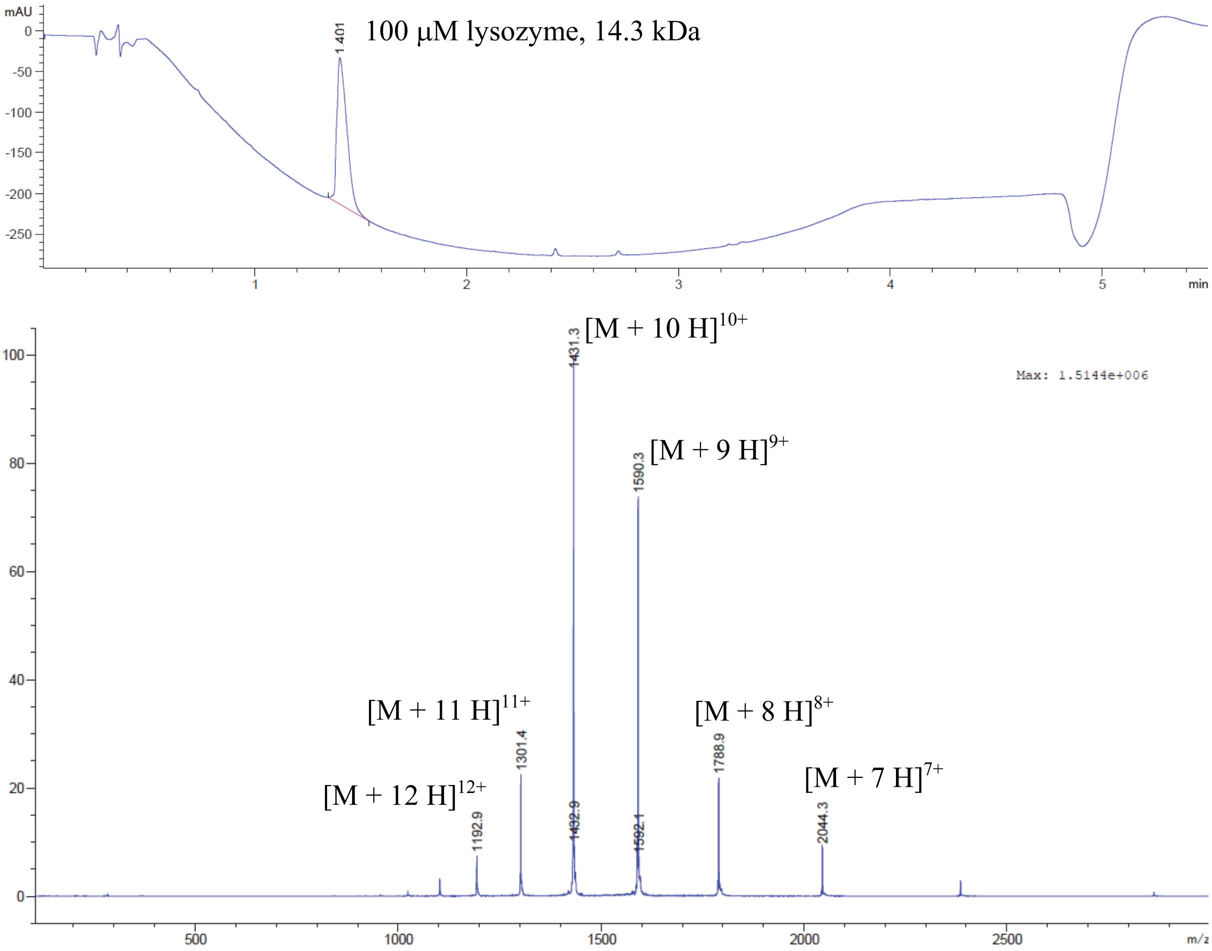

Figure S11: Top: Chromatogram of a 100 µM lysozyme solution in 100 mM KP_i, pH 7.20 recorded at 254 nm under acidic UPLC-MS conditions. **Bottom:** ESI-MS spectra of the unmodified lysozyme peak at t_R = 1.401 min containing the masses for the molecular ions with different protonation.

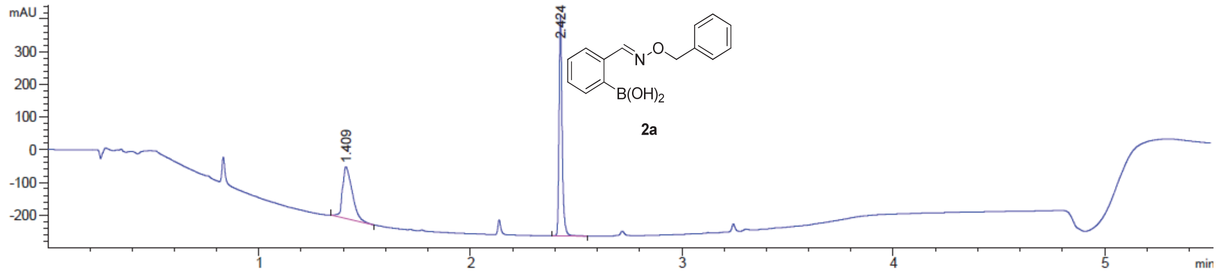

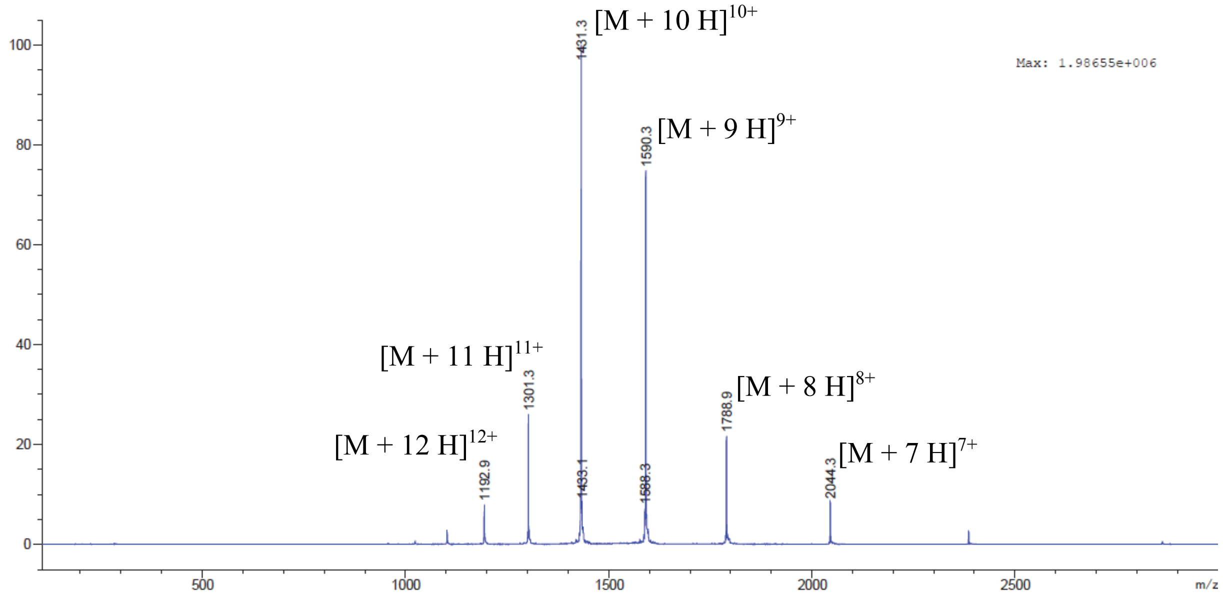

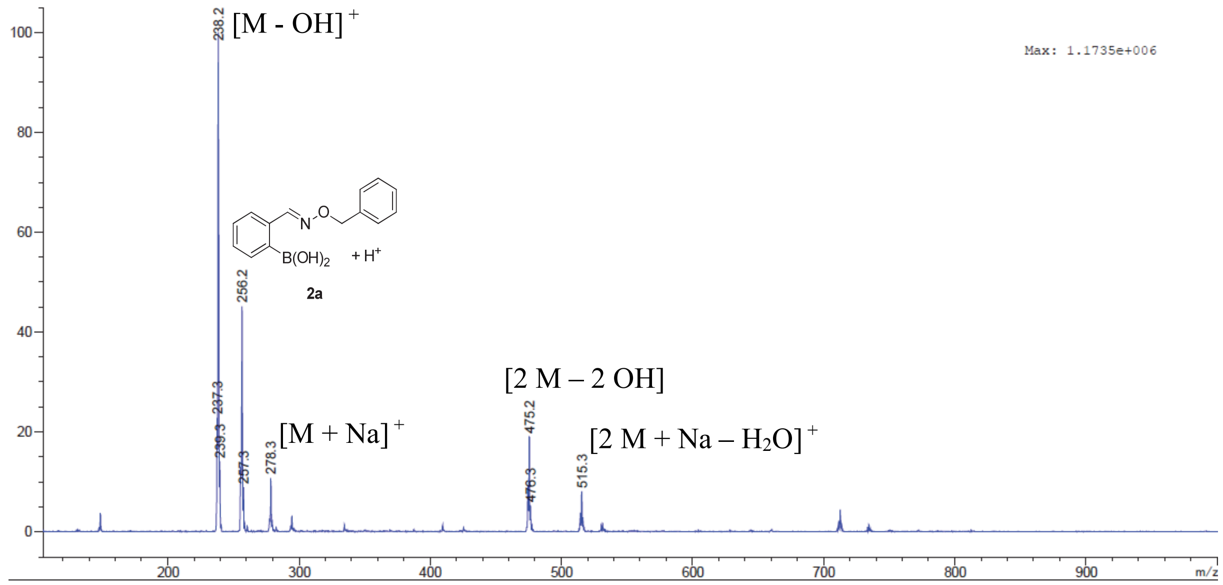

Figure S12: Top: Chromatogram of the reaction mixture of 100 µM 2-FPBA and 110 µM O-benzylhydroxylamine containing 100 µM of lysozyme as interfering additive in 100 mM KP_i, pH 7.20 recorded at 254 nm after 12 h under acidic UPLC-MS conditions. **Middle:** ESI-MS spectra of the unmodified lysozyme peak at t_R = 1.409 min. **Bottom:** ESI-MS spectra of the oxime **2a** peak at t_R = 2.424 min and its characteristic ionization adducts.

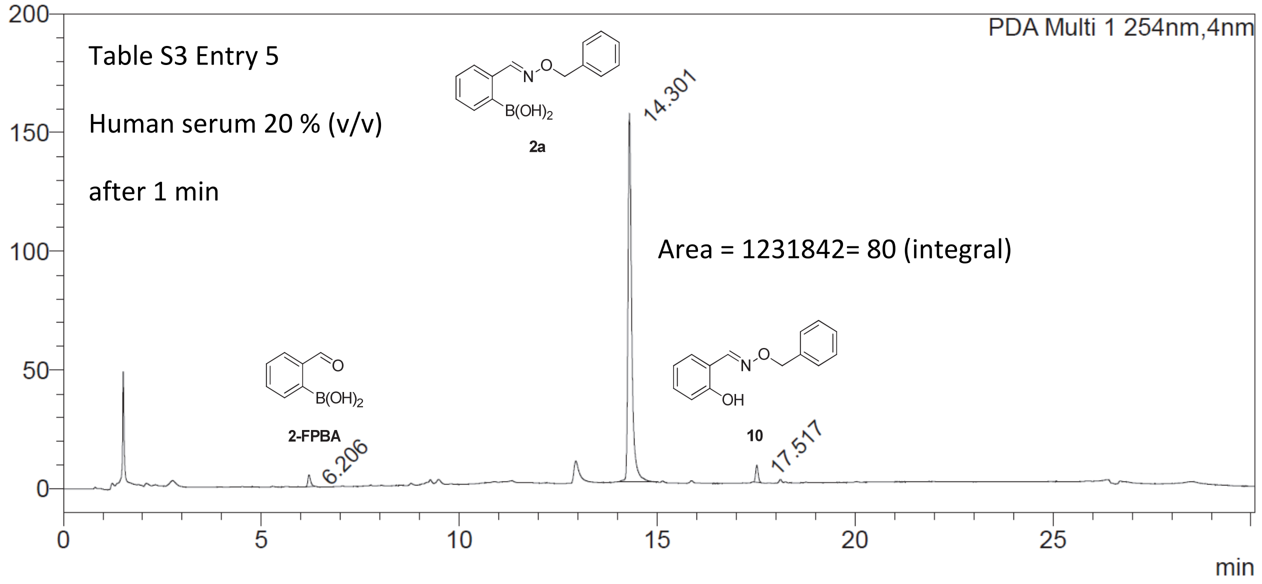

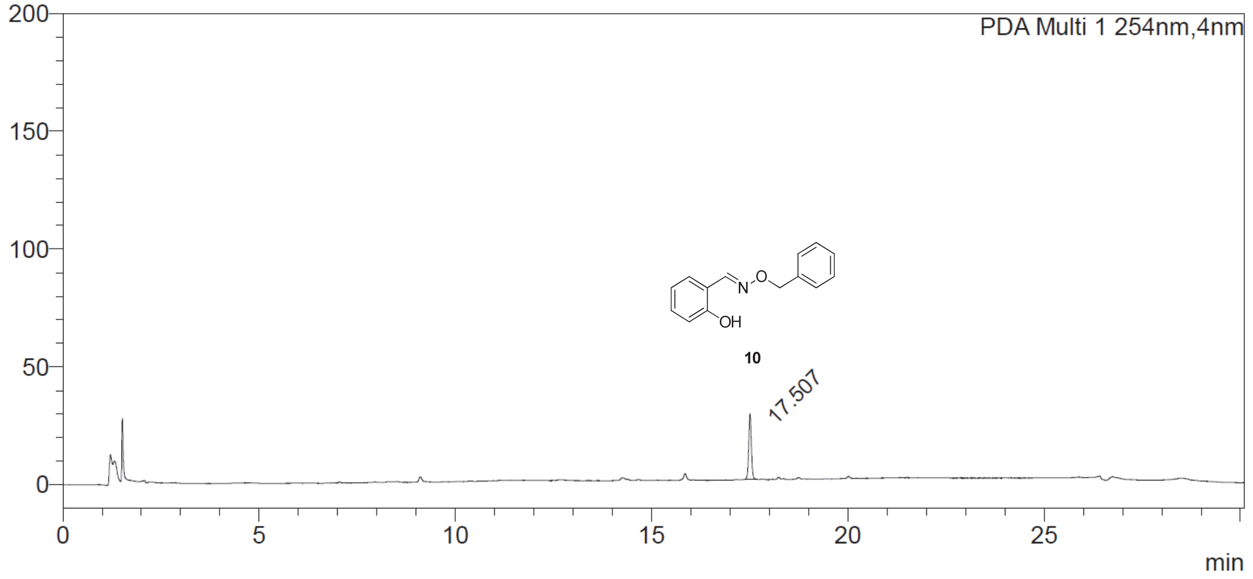

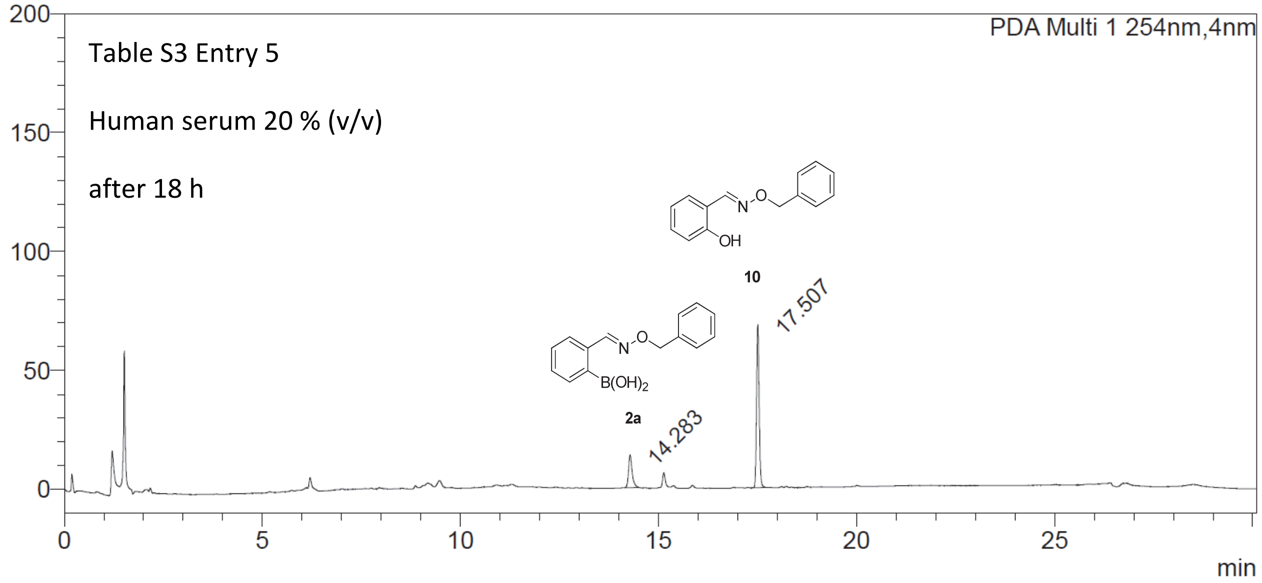

Figure S13: Top: Chromatogram of the reaction mixture of 100 µM 2-FPBA and 110 µM O-benzylhydroxylamine containing Human serum (20% v/v) as interfering additive in 100 mM KP_i, pH

7.20 recorded at 254 nm after 1 min under neutral HPLC conditions. The conversion of 2-FPBA is >98% and the normalized integral for oxime **2a** is 80. The peak at t_R = 17.5 min is the oxidation product **10** and was proven by separate injection (**middle**). **Bottom:** Chromatogram of the reaction mixture recorded after 18 h; clearly the oxime **2a** gets continuously oxidized in human serum and oxime **10** is the major product.

Oxime **2a** and **10** (Table S3 Entry 5) were separately verified by UPLC-MS injections after 5 min and 18 h and their corresponding ESI-MS traces are given in Figure S14.

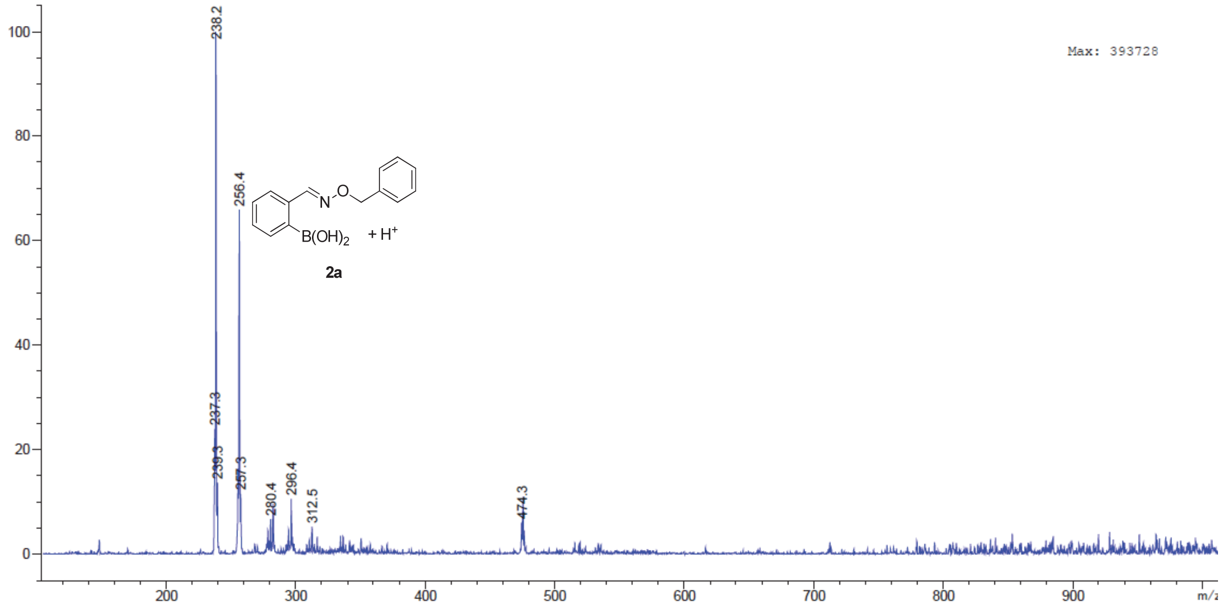

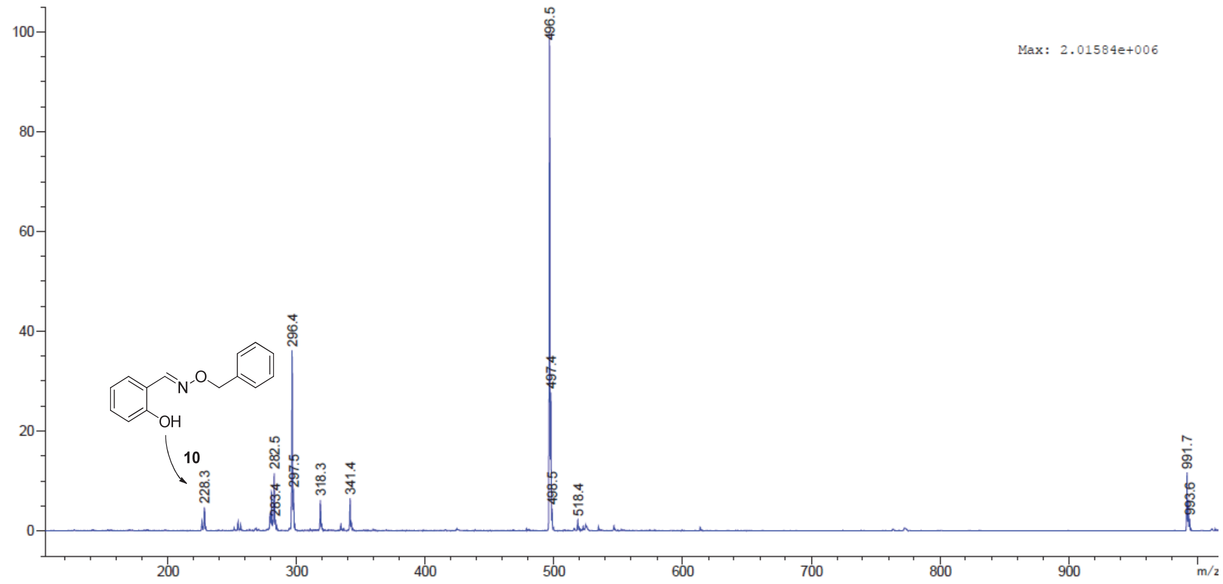

Figure S14: Top: ESI-MS trace of the peak at t_R = 14.3 min indicating oxime **2a**. **Bottom:** ESI-MS trace of the peak at t_R = 17.5 min indicating oxime **10**; masses which are not assigned with a reasonable structure are assumed to be a result of the complex components in human serum.

7. NMR spectra

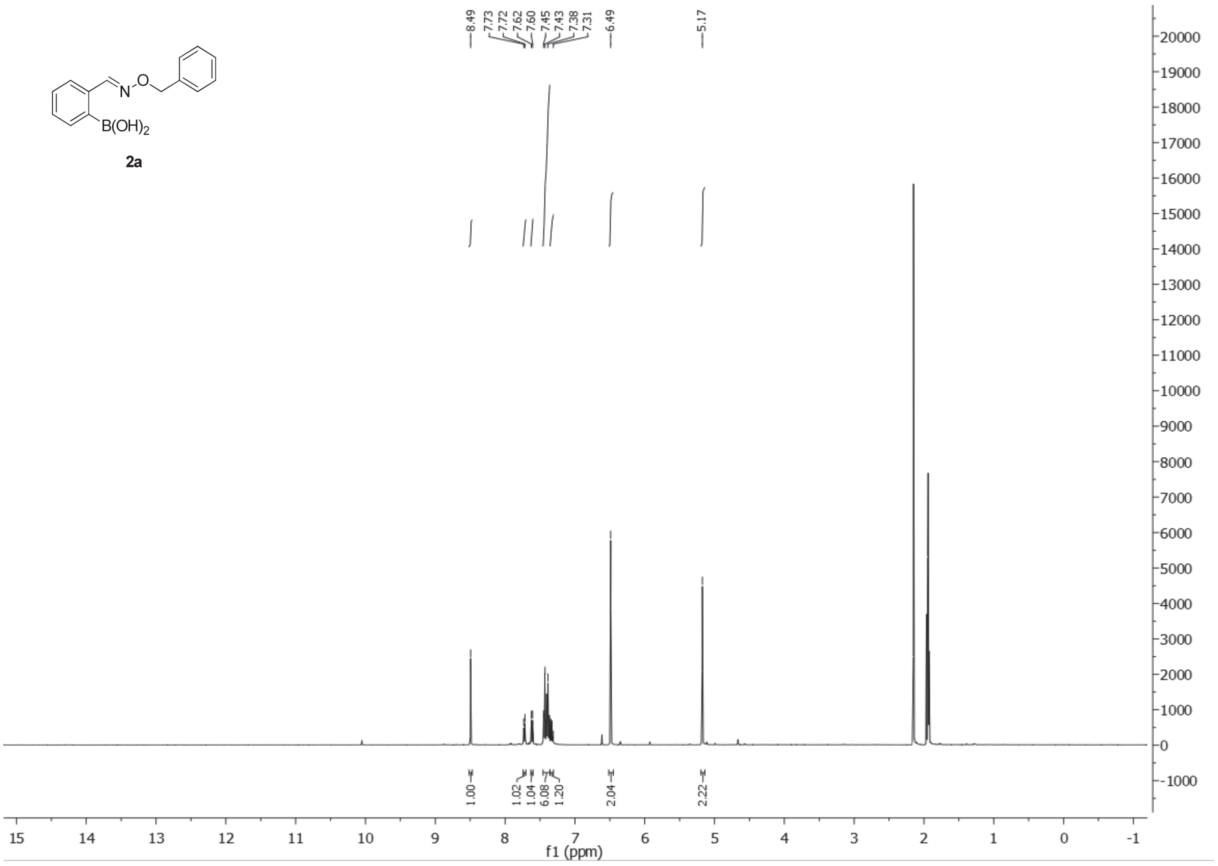

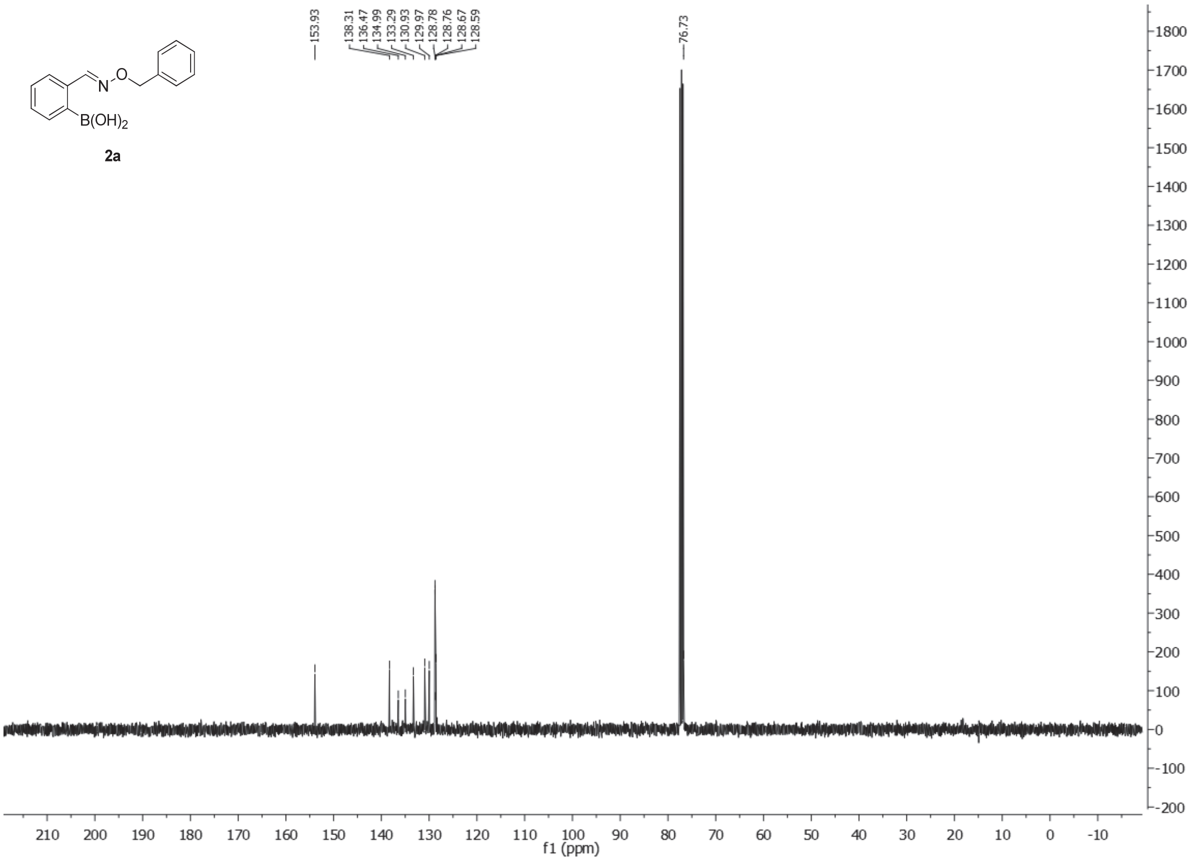

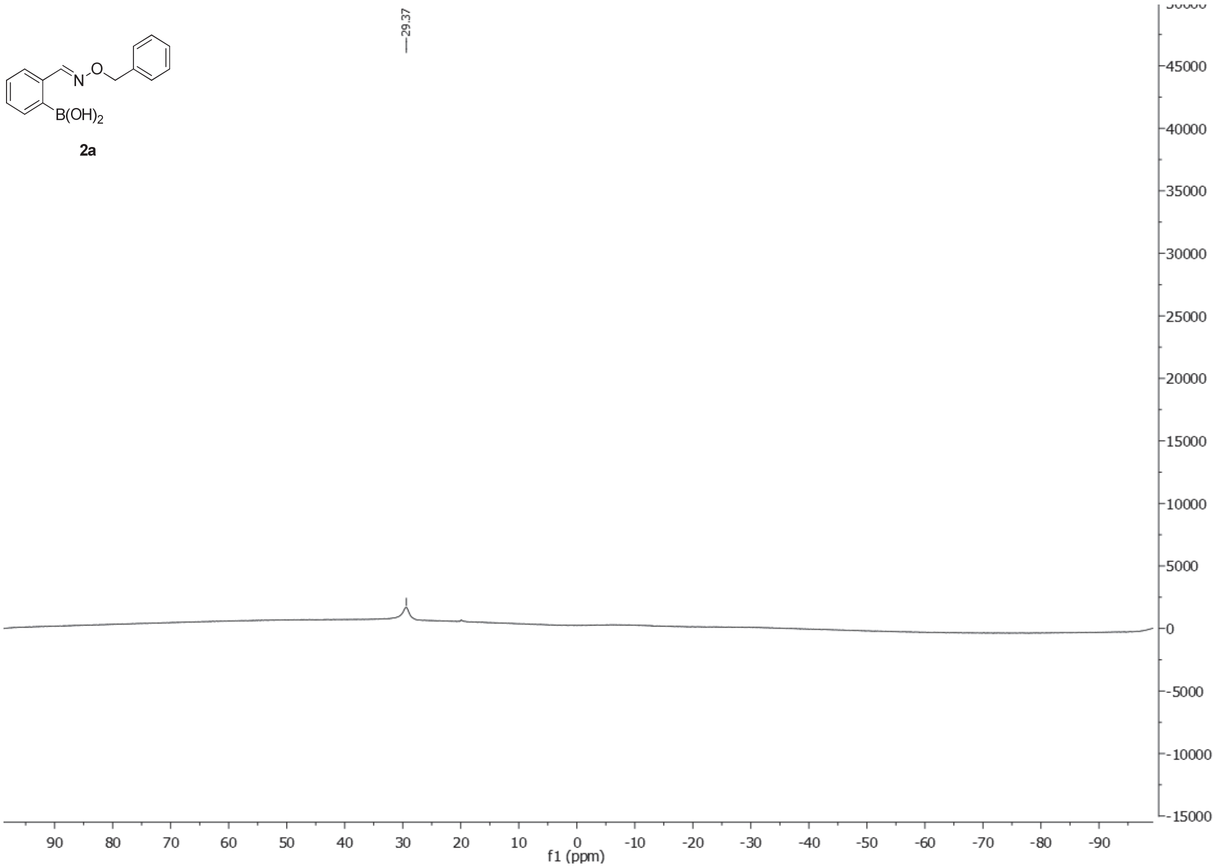
29.37
f1 (ppm)

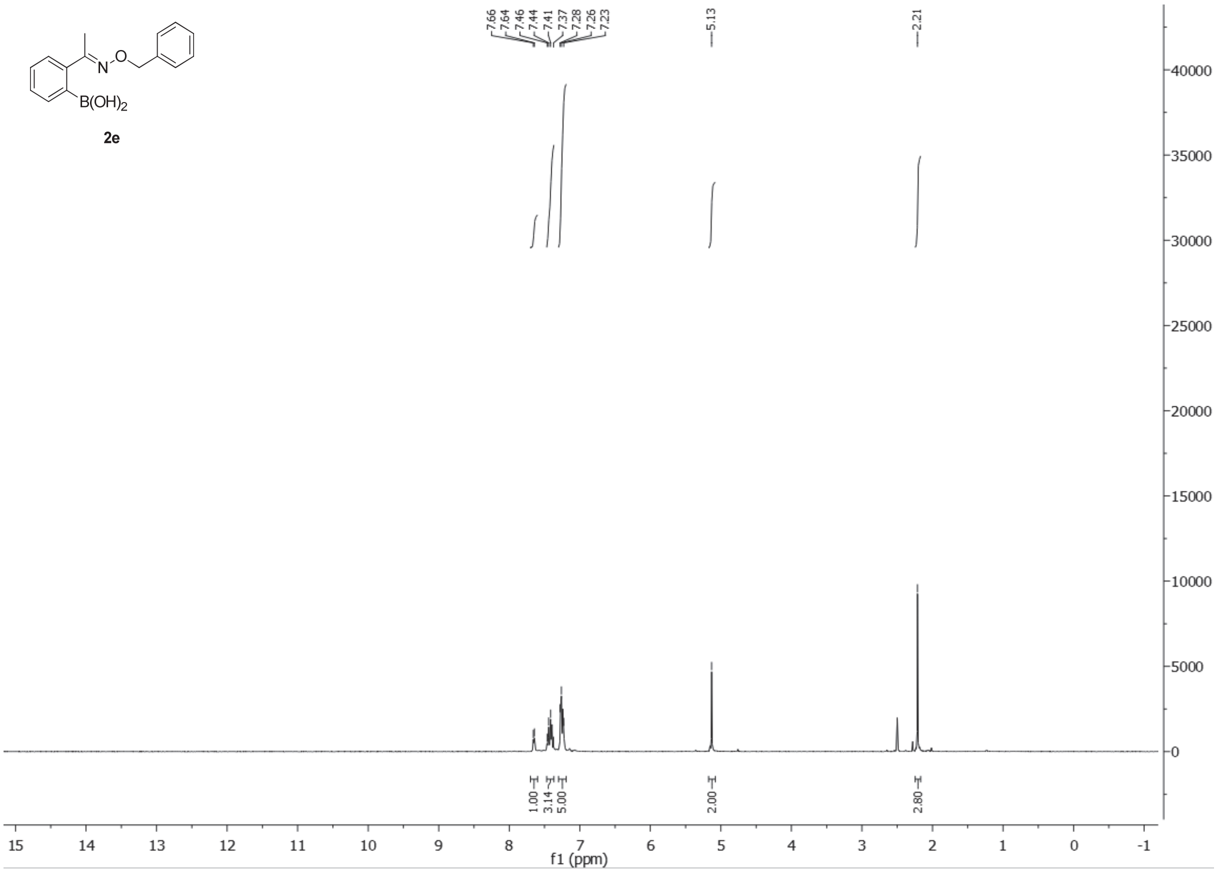
7.66
7.64
7.46
7.44
7.41
7.37
7.28
7.26
7.23
5.13
2.21
1.00
3.14
5.00
2.00
2.80
f1 (ppm)

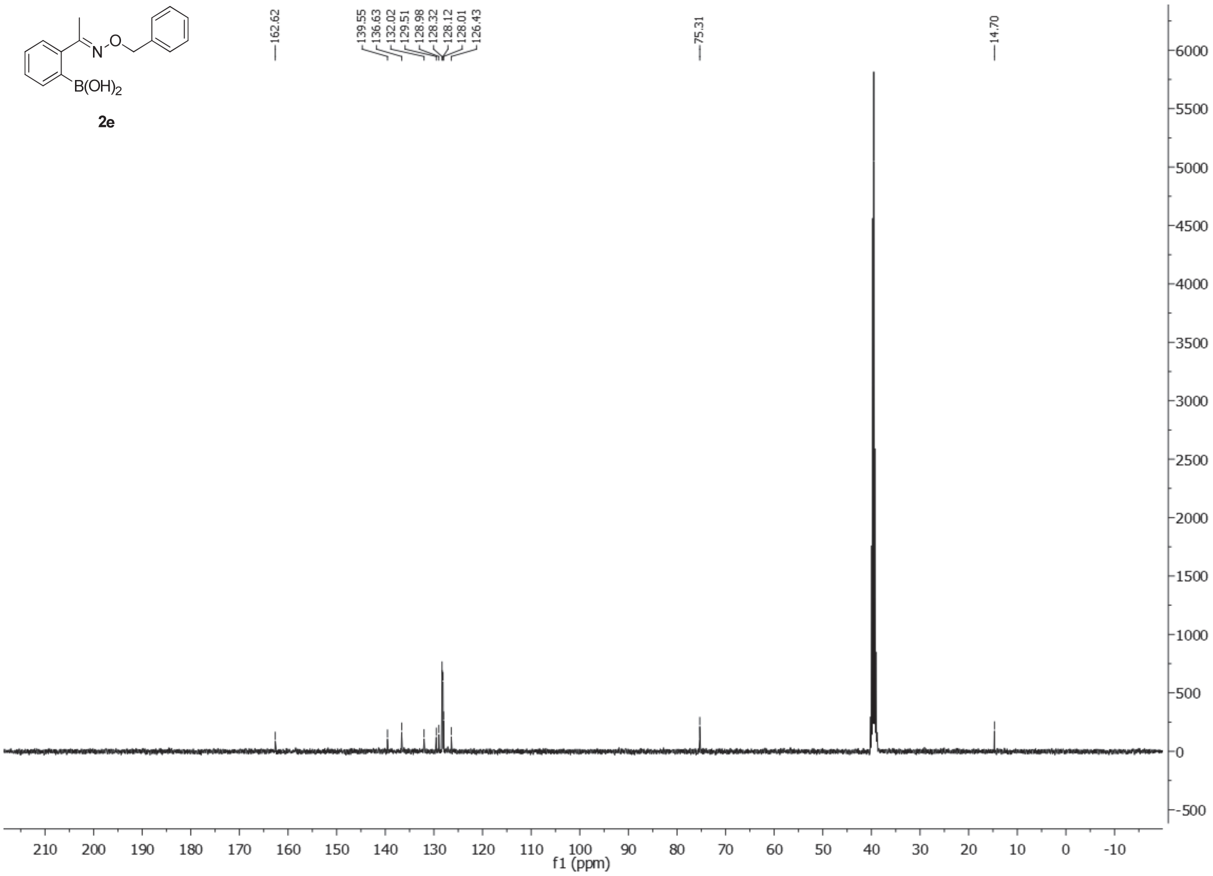
B(OH)2
N
O
162.62
139.55
136.63
132.02
129.51
128.98
128.32
128.12
128.01
126.43
75.31
14.70
f1 (ppm)

2e

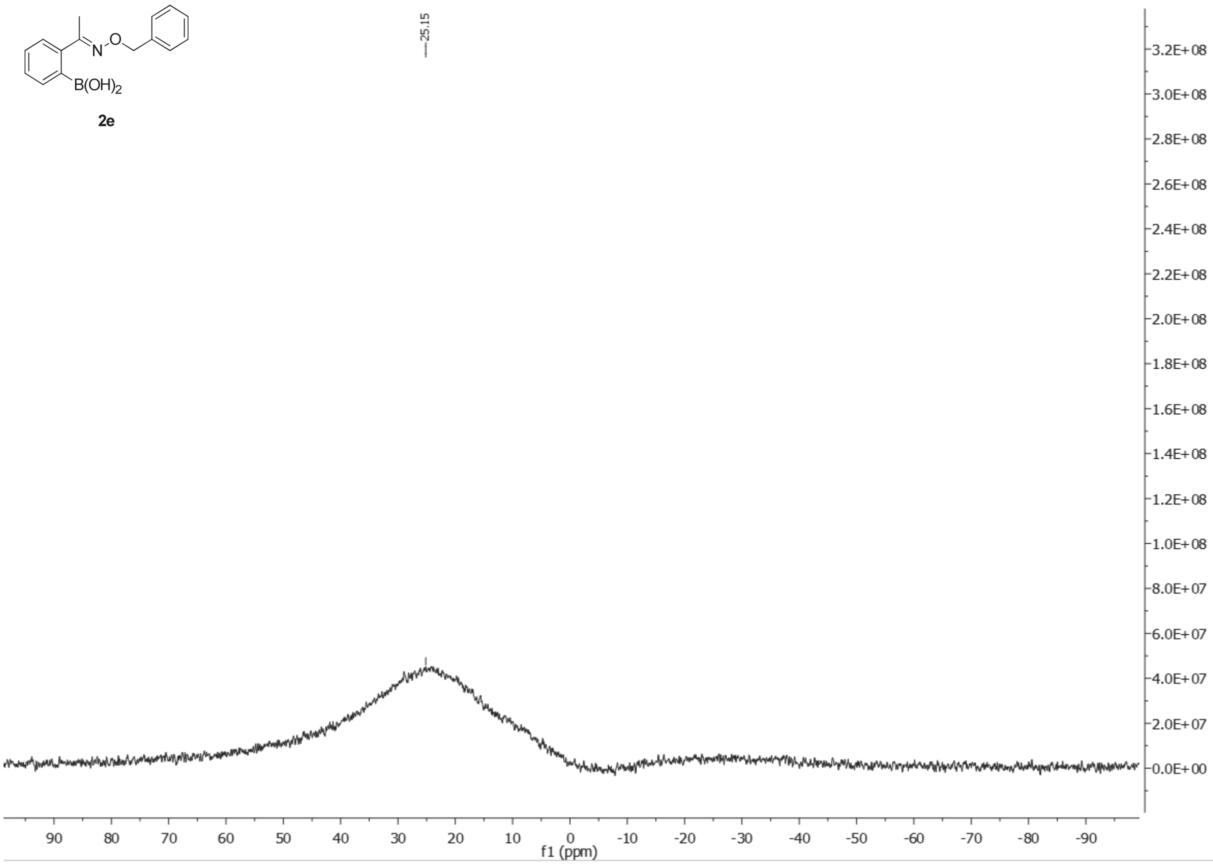
B(OH)2
N
O
25.15
f1 (ppm)

2e

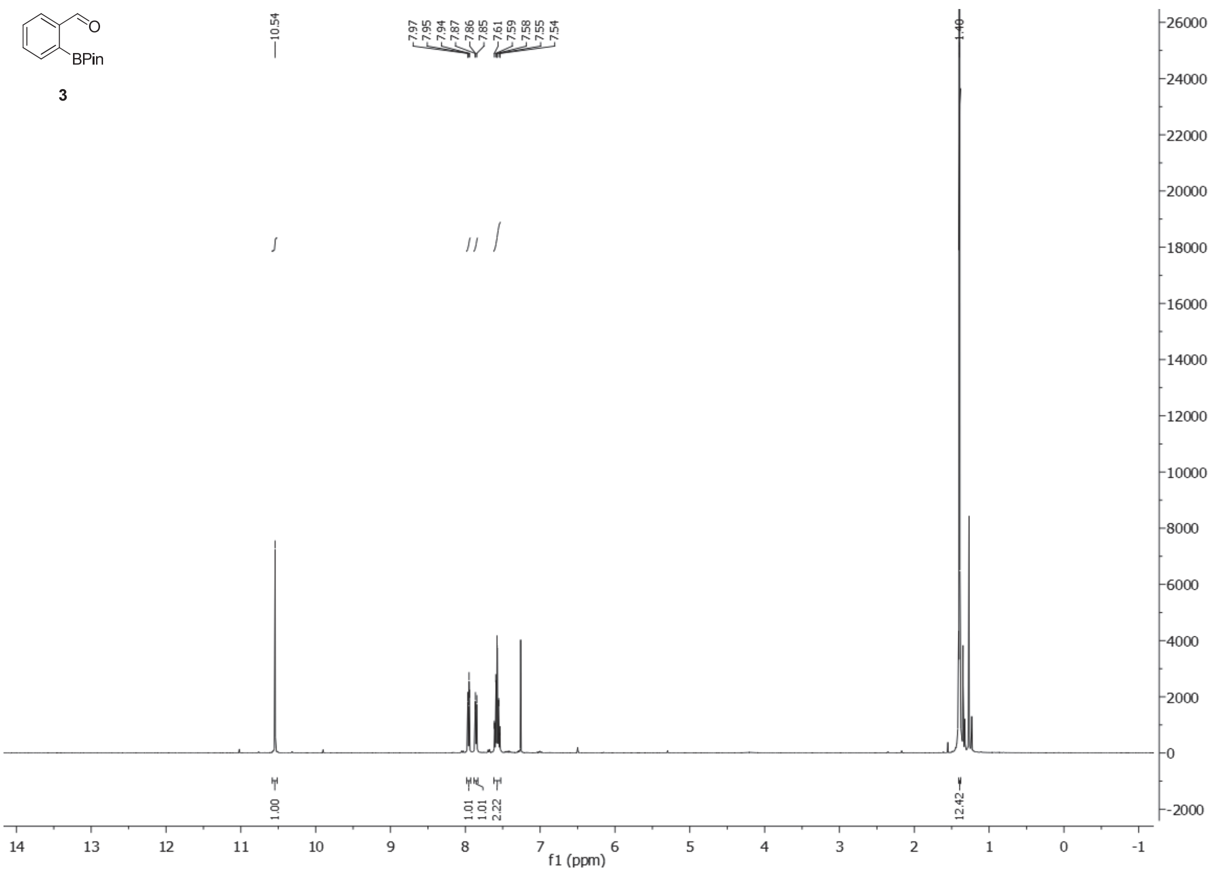

O
BPin
3
10.54
7.97
7.95
7.94
7.87
7.86
7.85
7.61
7.59
7.58
7.55
7.54
1.40
1.00
1.01
1.01
2.22
12.42
f1 (ppm)

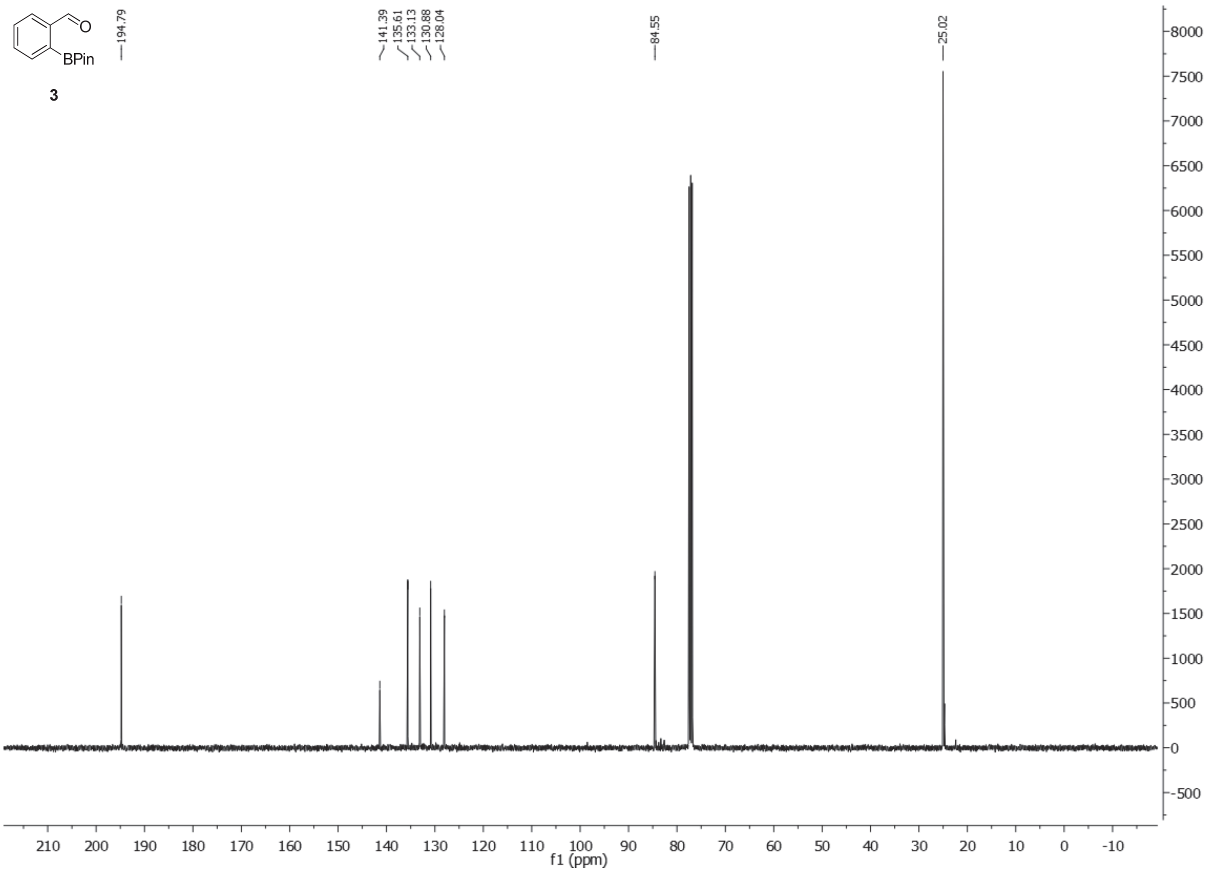

O
BPin
3
194.79
141.39
135.61
133.13
130.88
128.04
84.55
25.02
f1 (ppm)

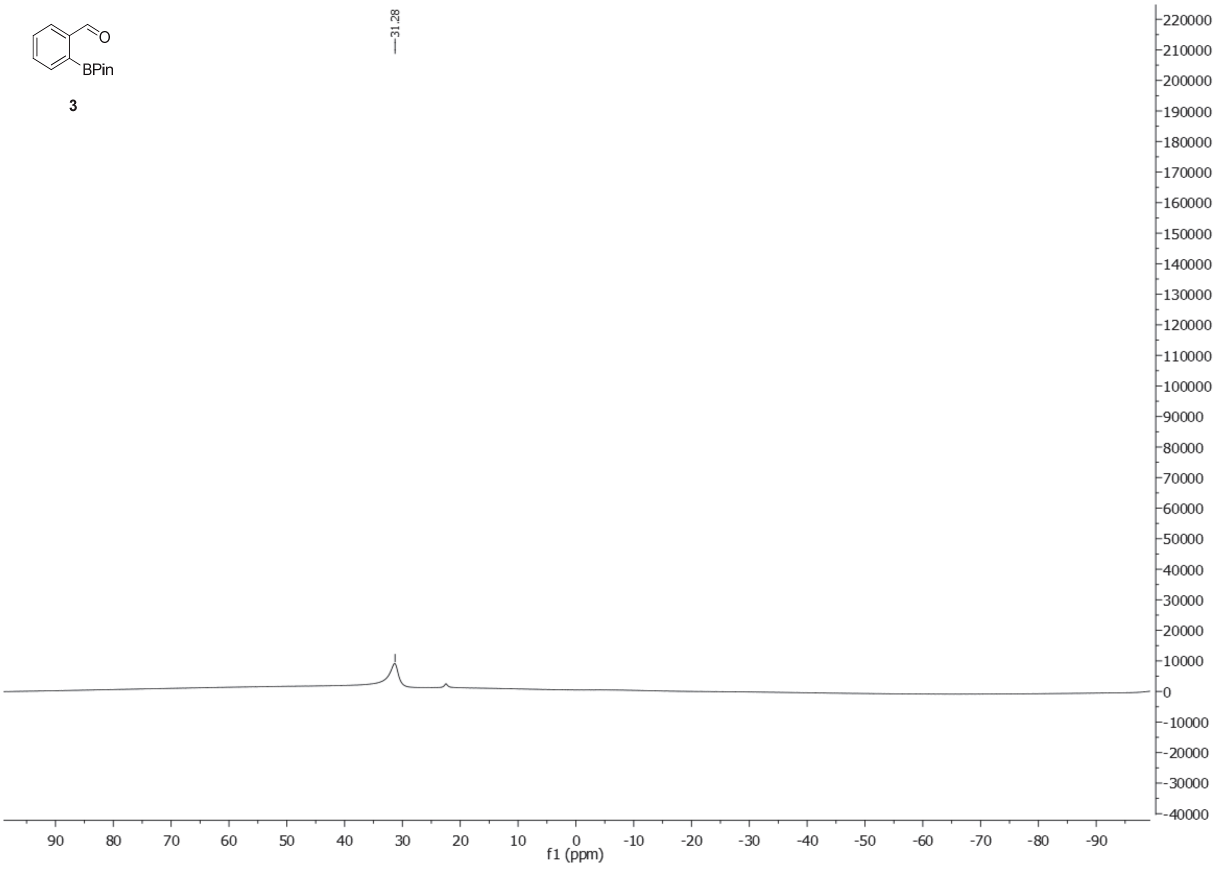

BPin
3
31.28
f1 (ppm)

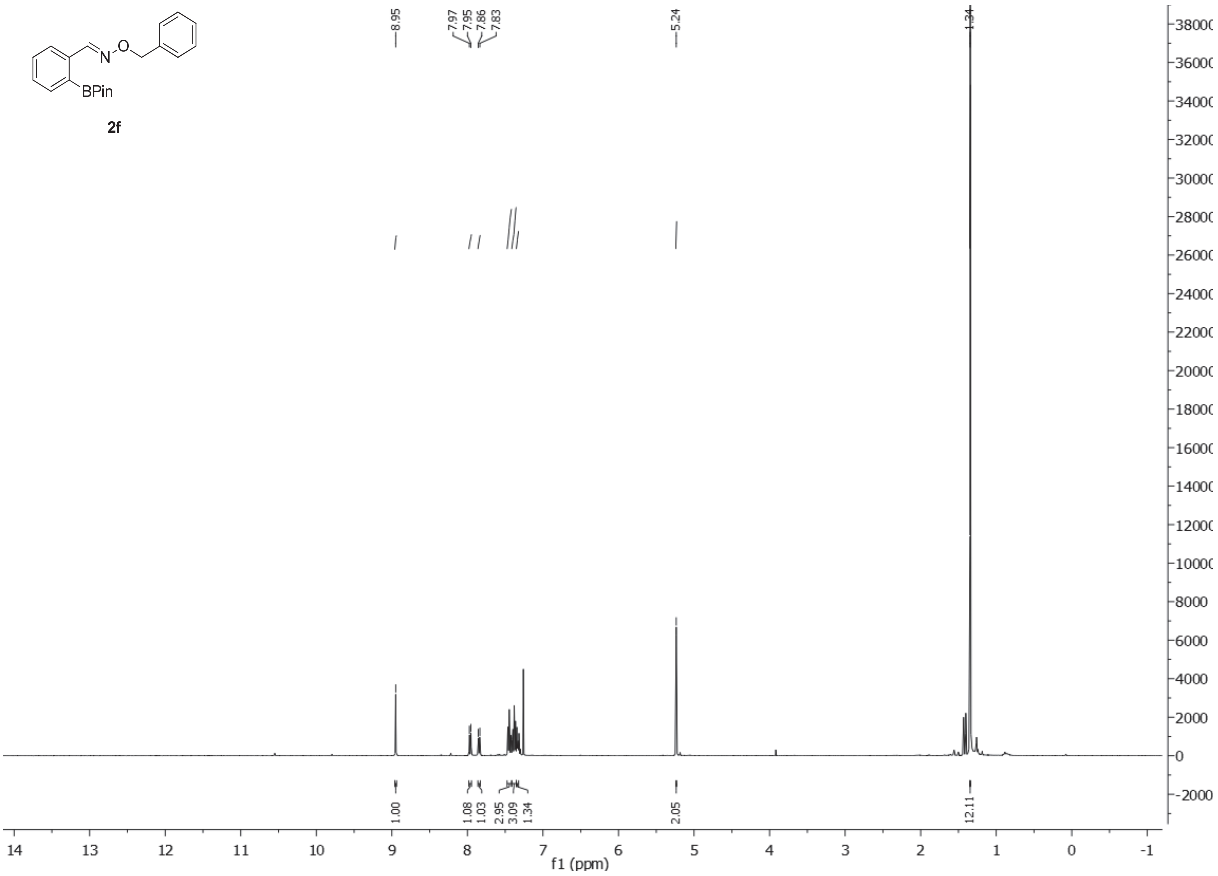

N
O
BPin
2f
8.95
7.97
7.95
7.86
7.83
5.24
1.34
1.00
1.08
1.03
2.95
3.09
1.34
2.05
12.11
f1 (ppm)

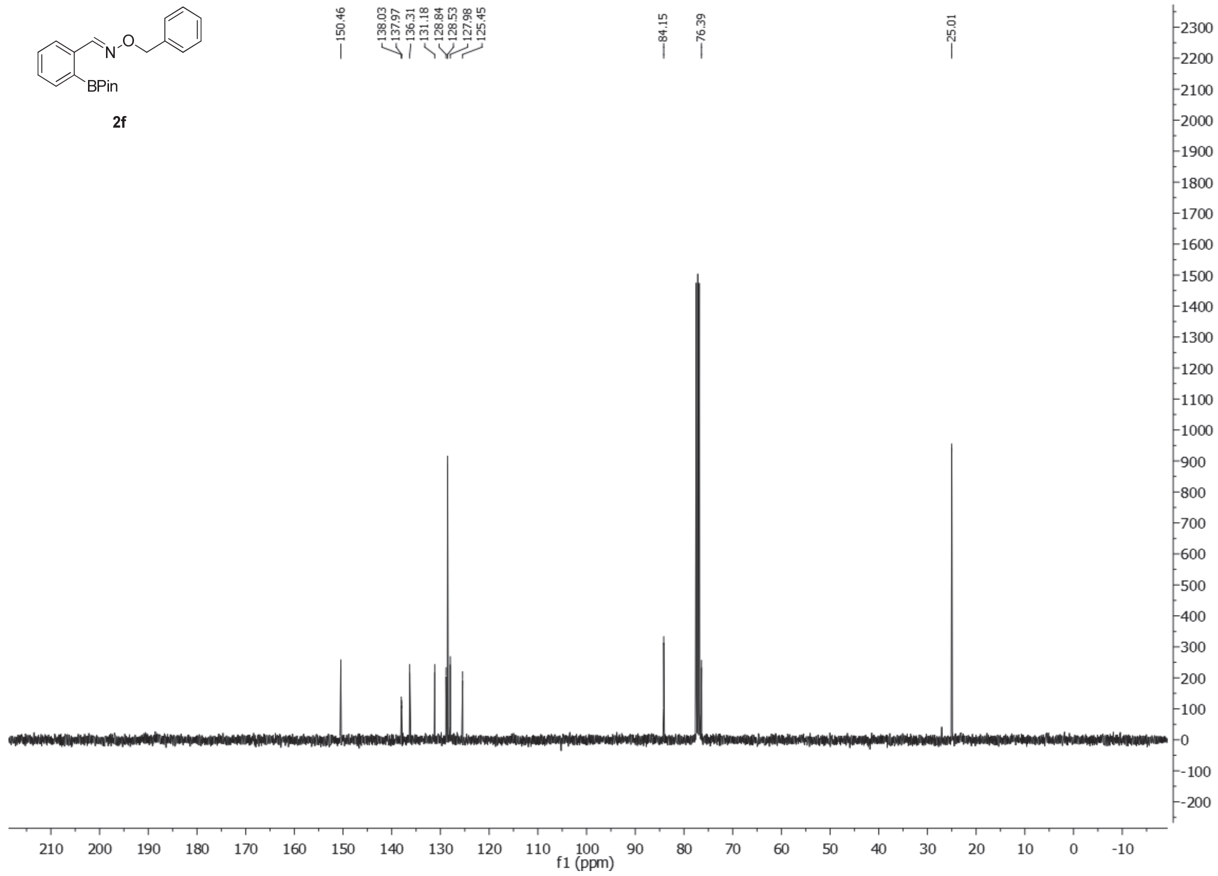
BPin
2f
150.46
138.03
137.97
136.31
131.18
128.84
128.53
127.98
125.45
84.15
76.39
25.01
f1 (ppm)

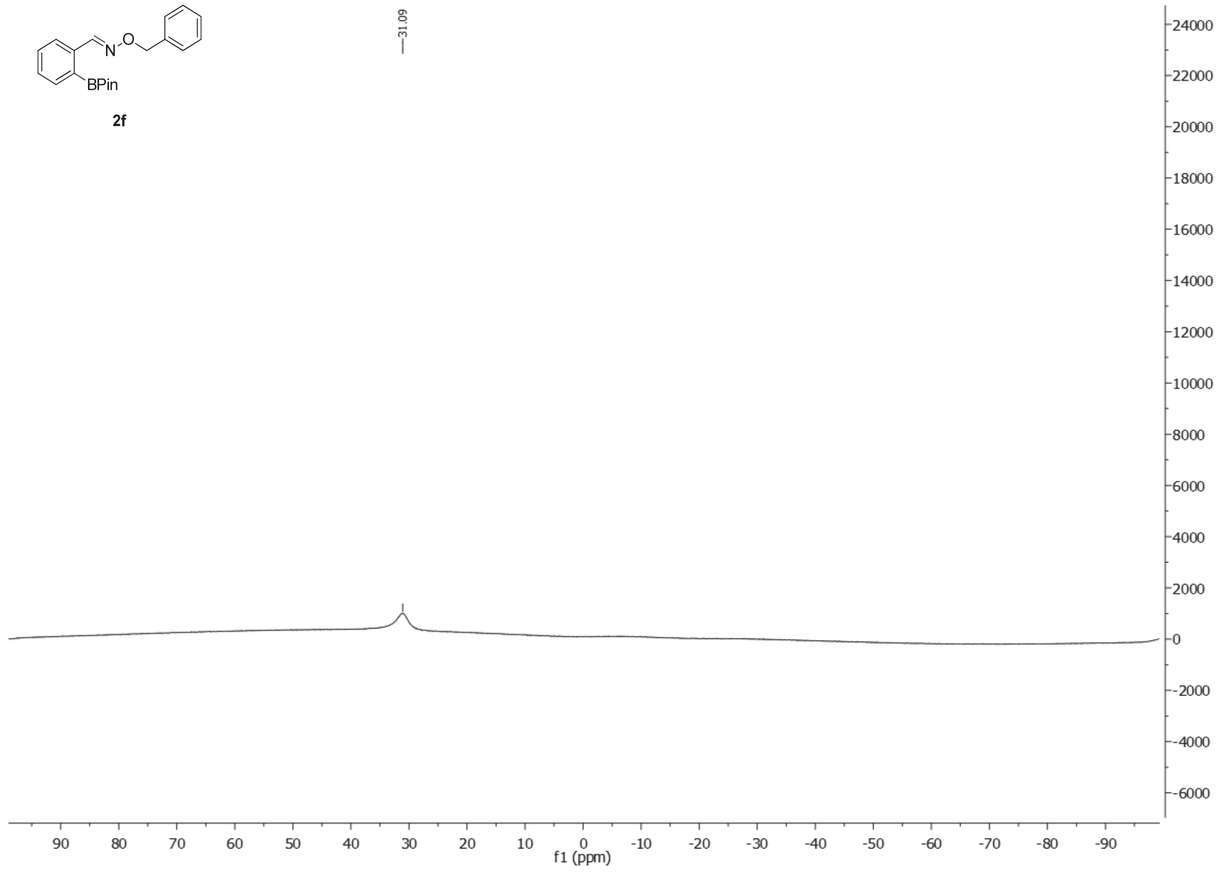
BPin
2f
31.09
f1 (ppm)

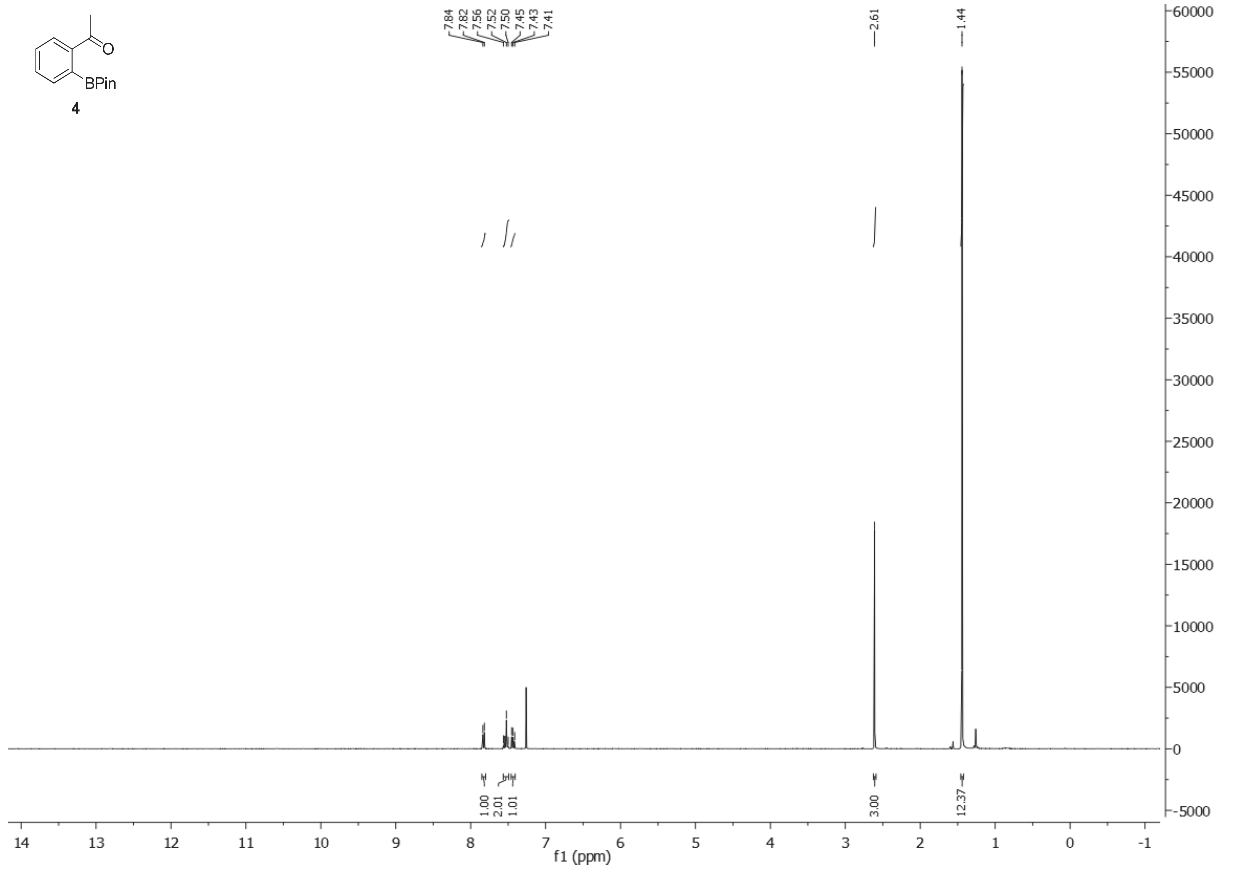

BPin
4
7.84 7.82 7.56 7.52 7.50 7.45 7.43 7.41
2.61
1.44
1.00 2.01 1.01
3.00
12.37
f1 (ppm)

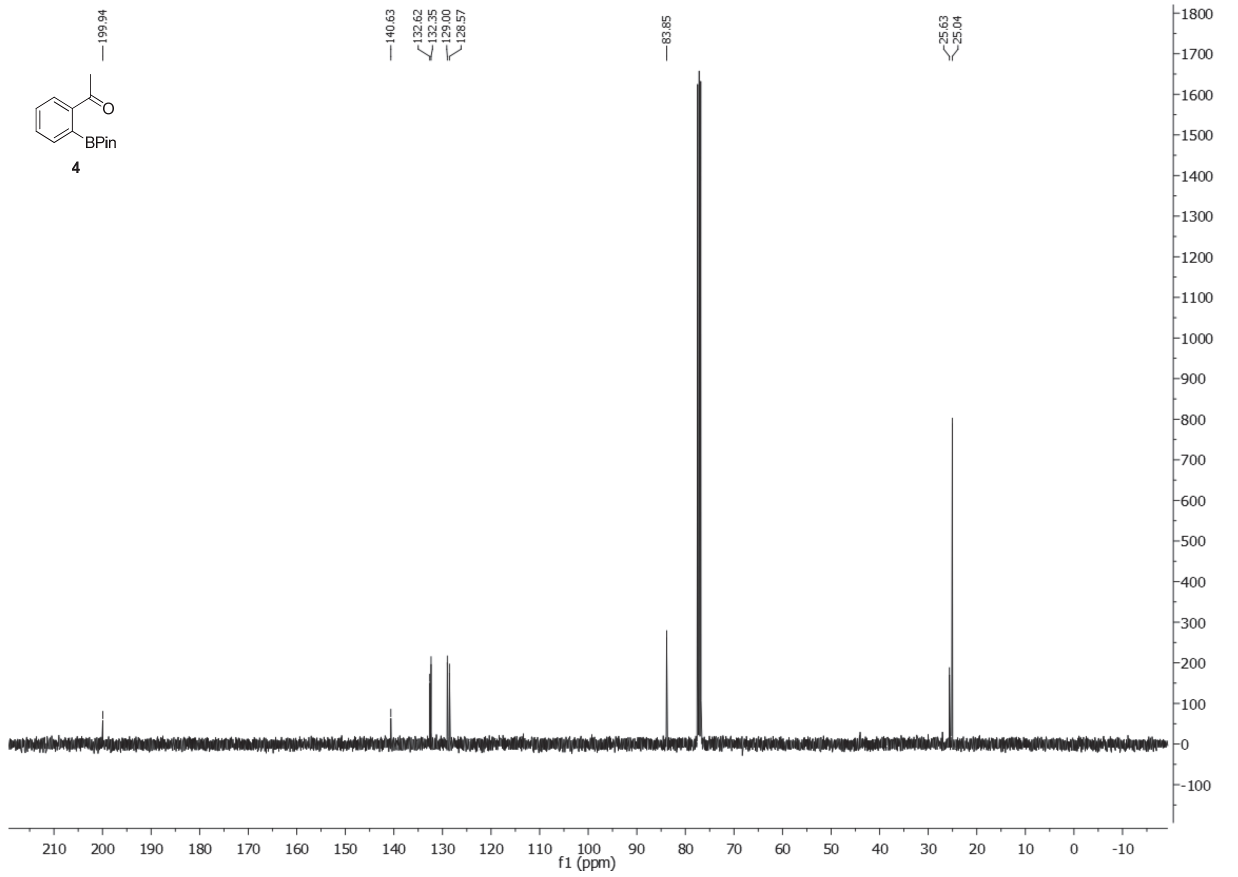

BPin
4
199.94
140.63 132.62 132.35 129.00 128.57
83.85
25.63 25.04
f1 (ppm)

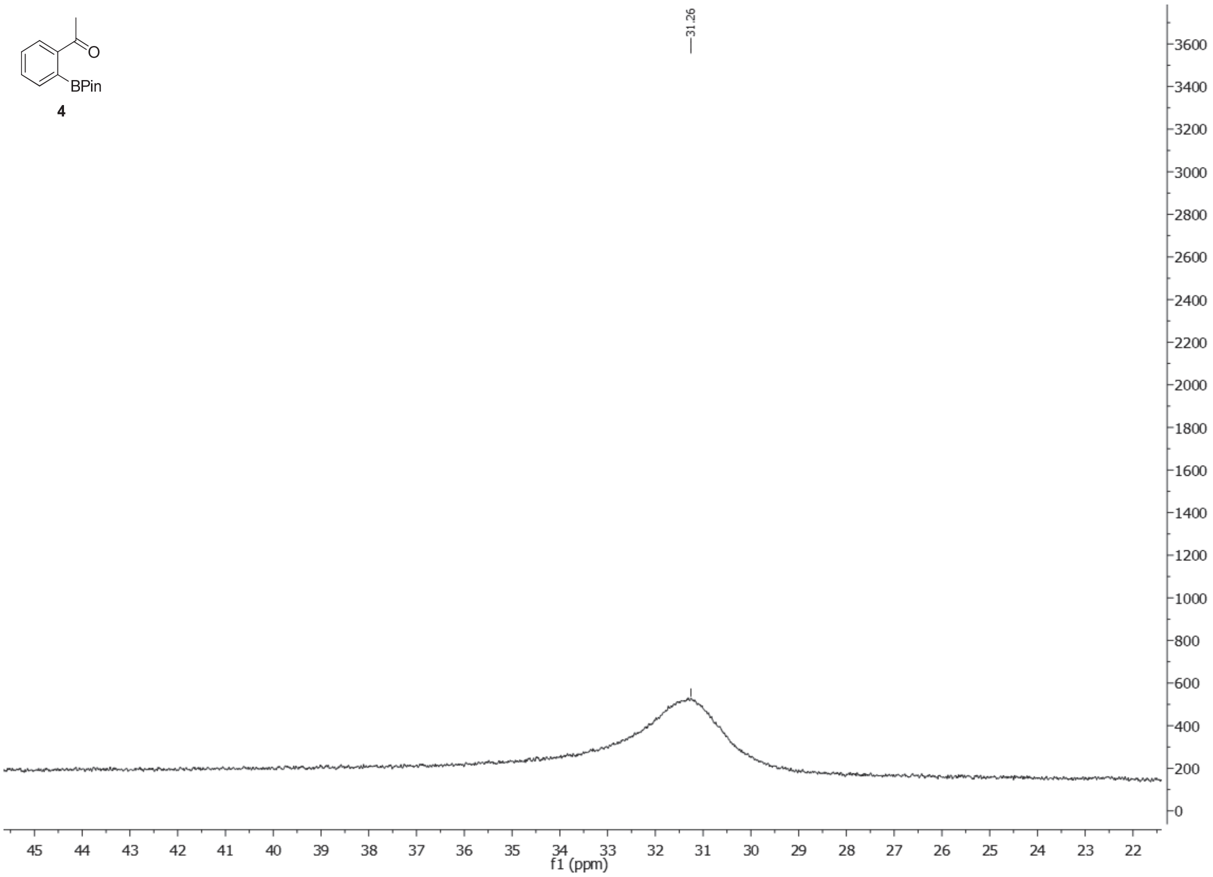
BPin
4
31.26
f1 (ppm)

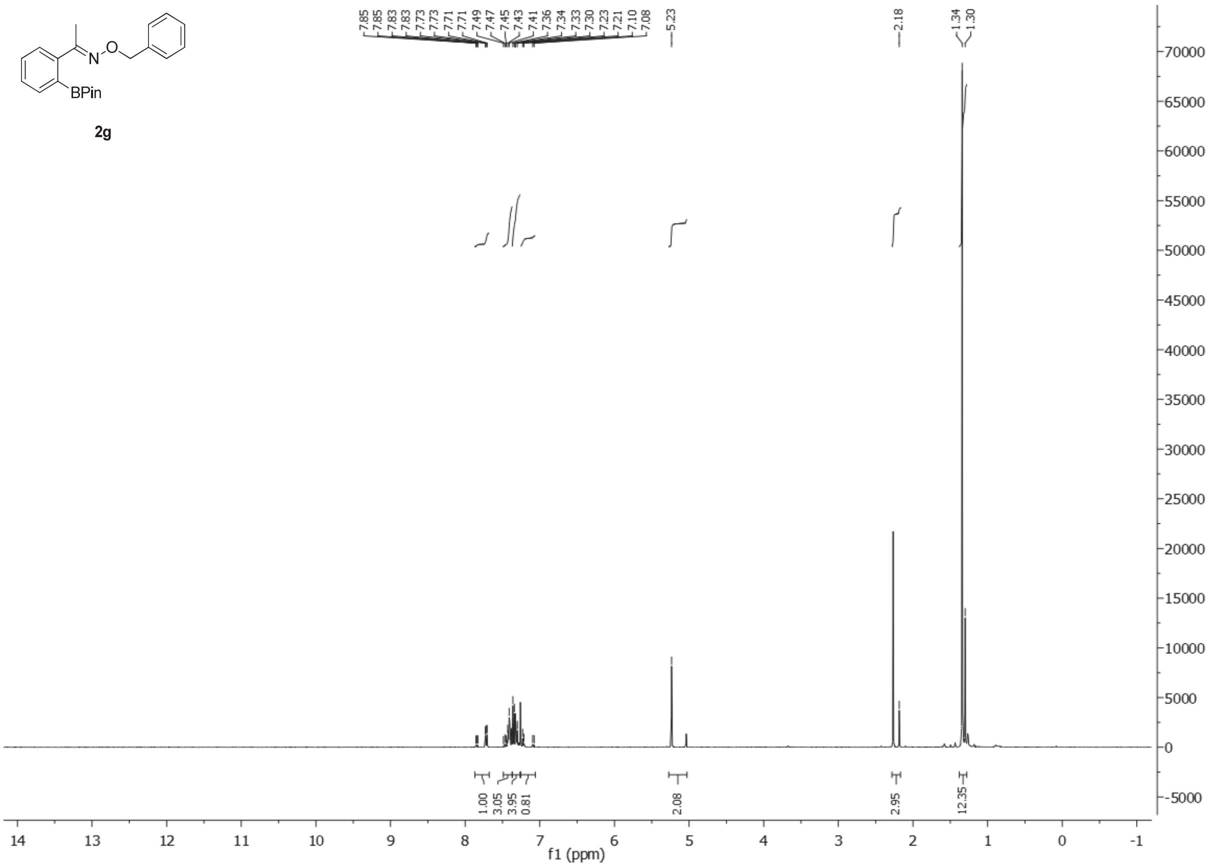
N
O
BPin
2g
7.85
7.85
7.83
7.83
7.73
7.73
7.71
7.71
7.49
7.47
7.45
7.43
7.41
7.36
7.34
7.33
7.30
7.23
7.21
7.10
7.08
5.23
2.18
1.34
1.30
1.00
3.05
3.95
0.81
2.08
2.95
12.35
f1 (ppm)

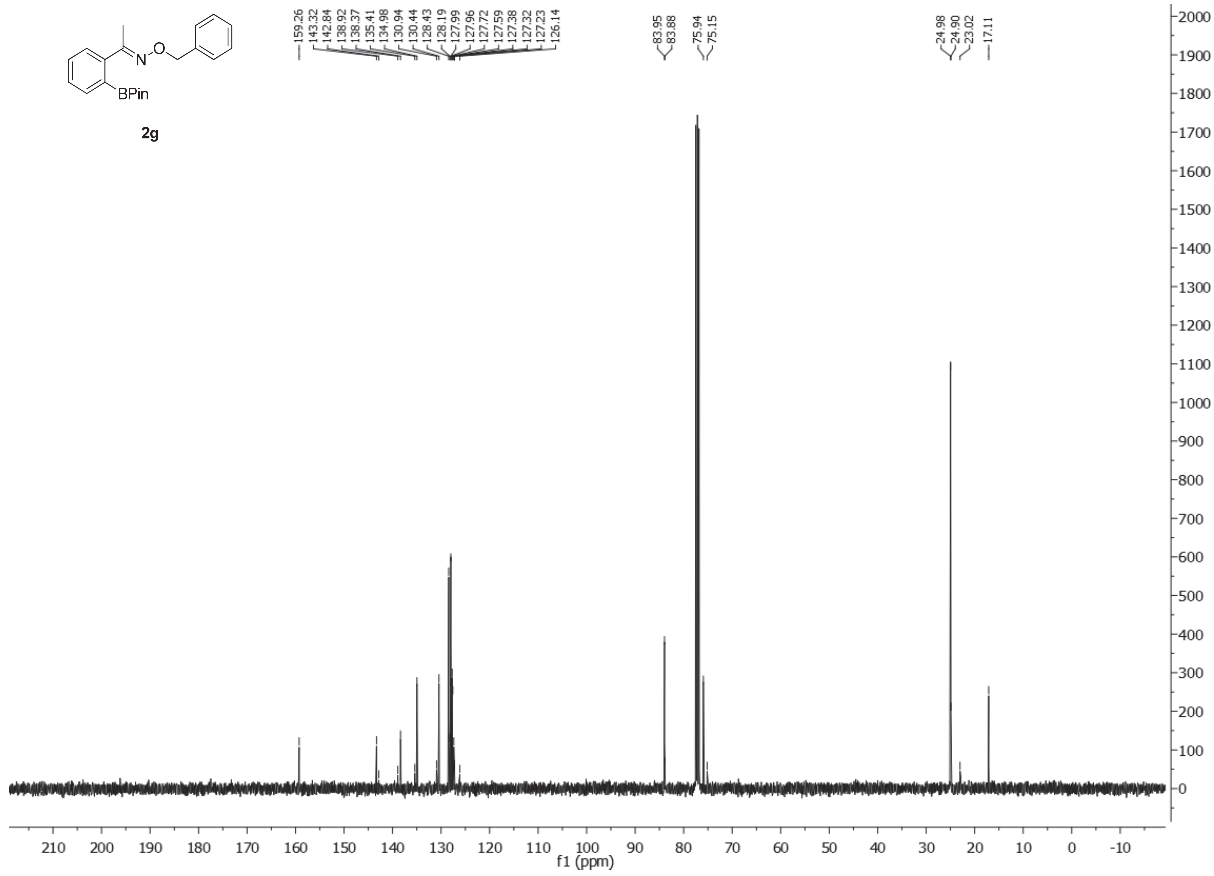
N
O
BPin
159.26
143.32
142.84
138.92
138.37
135.41
134.98
130.94
130.44
128.43
128.19
127.99
127.96
127.72
127.59
127.38
127.32
127.23
126.14
83.95
83.88
75.94
75.15
24.98
24.90
23.02
17.11
f1 (ppm)

2g

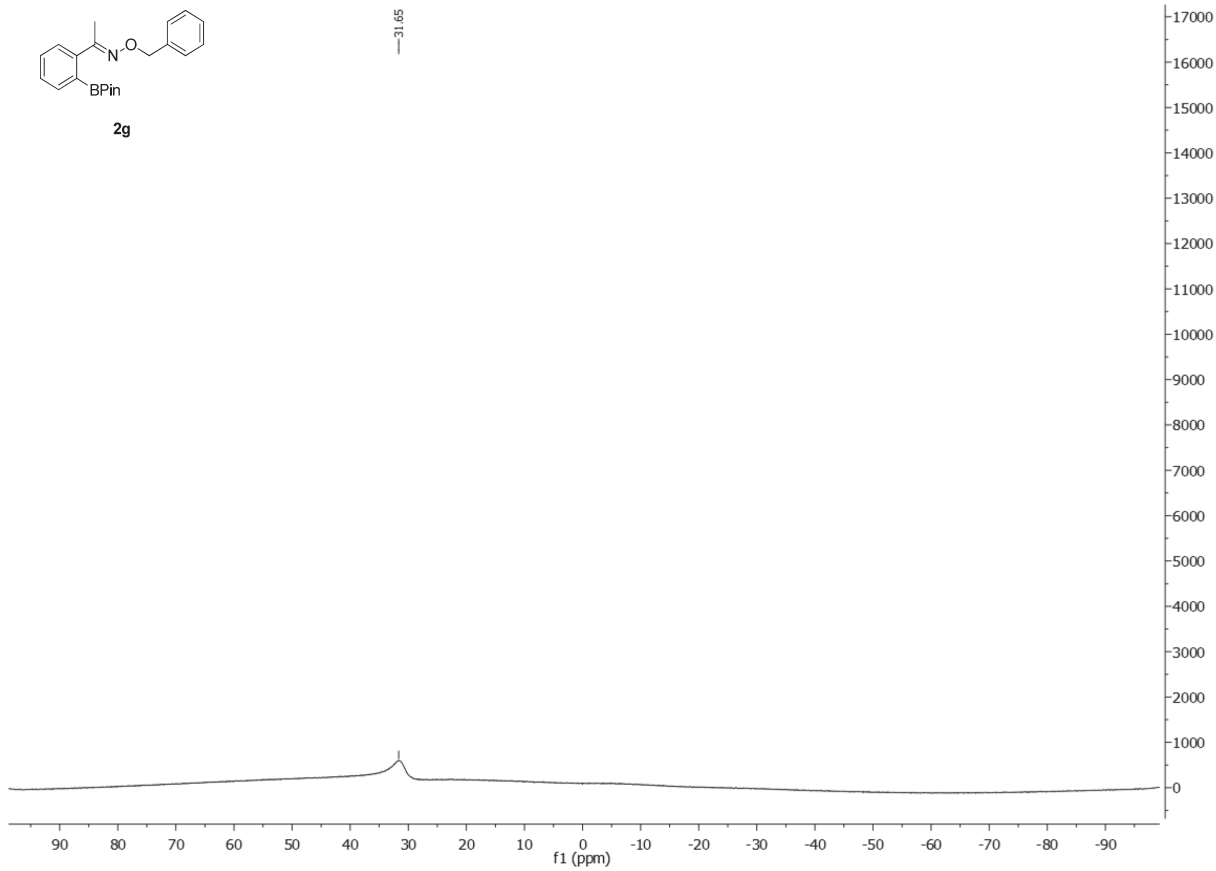
N
O
BPin
31.65
f1 (ppm)

2g

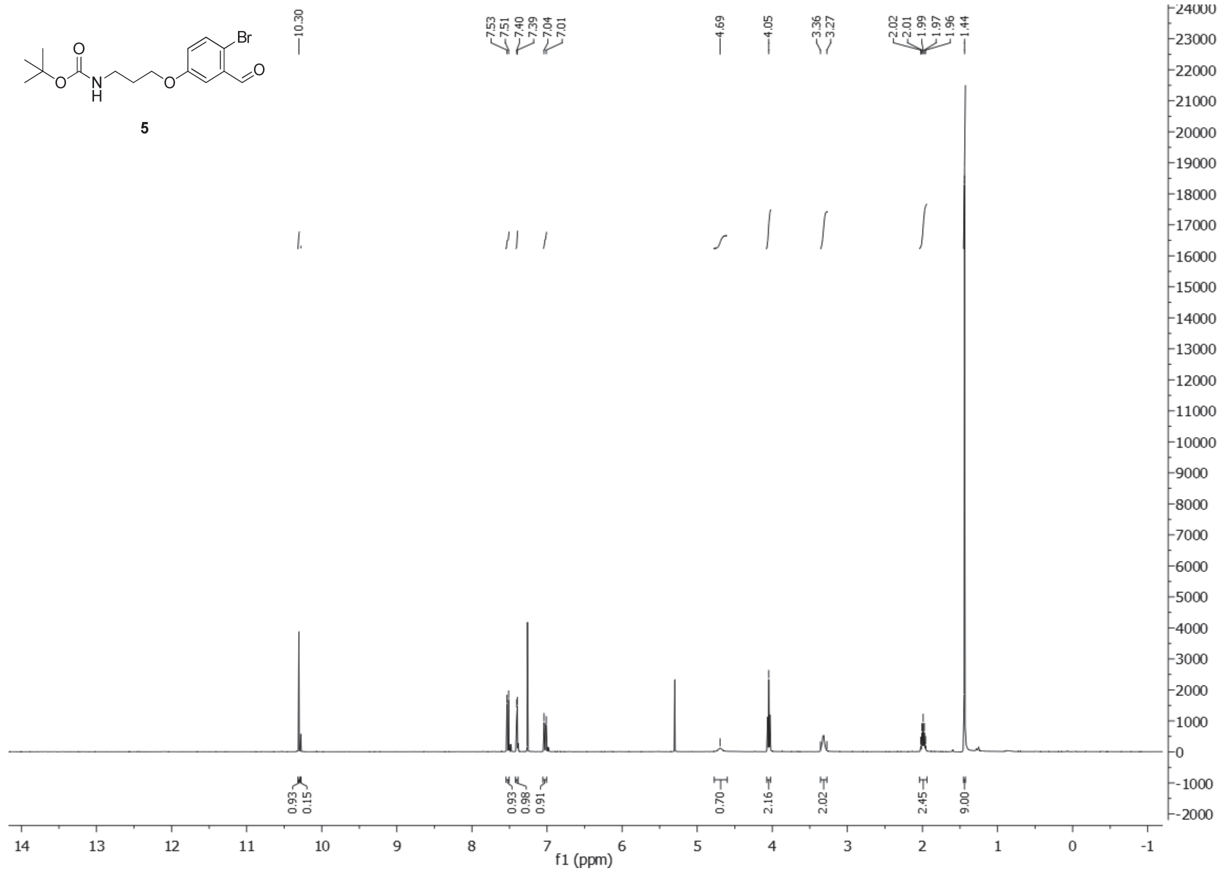
10.30
7.53
7.51
7.40
7.39
7.04
7.01
4.69
4.05
3.36
3.27
2.02
2.01
1.99
1.97
1.96
1.44
Br
O
O
N
H
O
O
5
0.93
0.15
0.93
0.98
0.91
0.70
2.16
2.02
2.45
9.00
f1 (ppm)

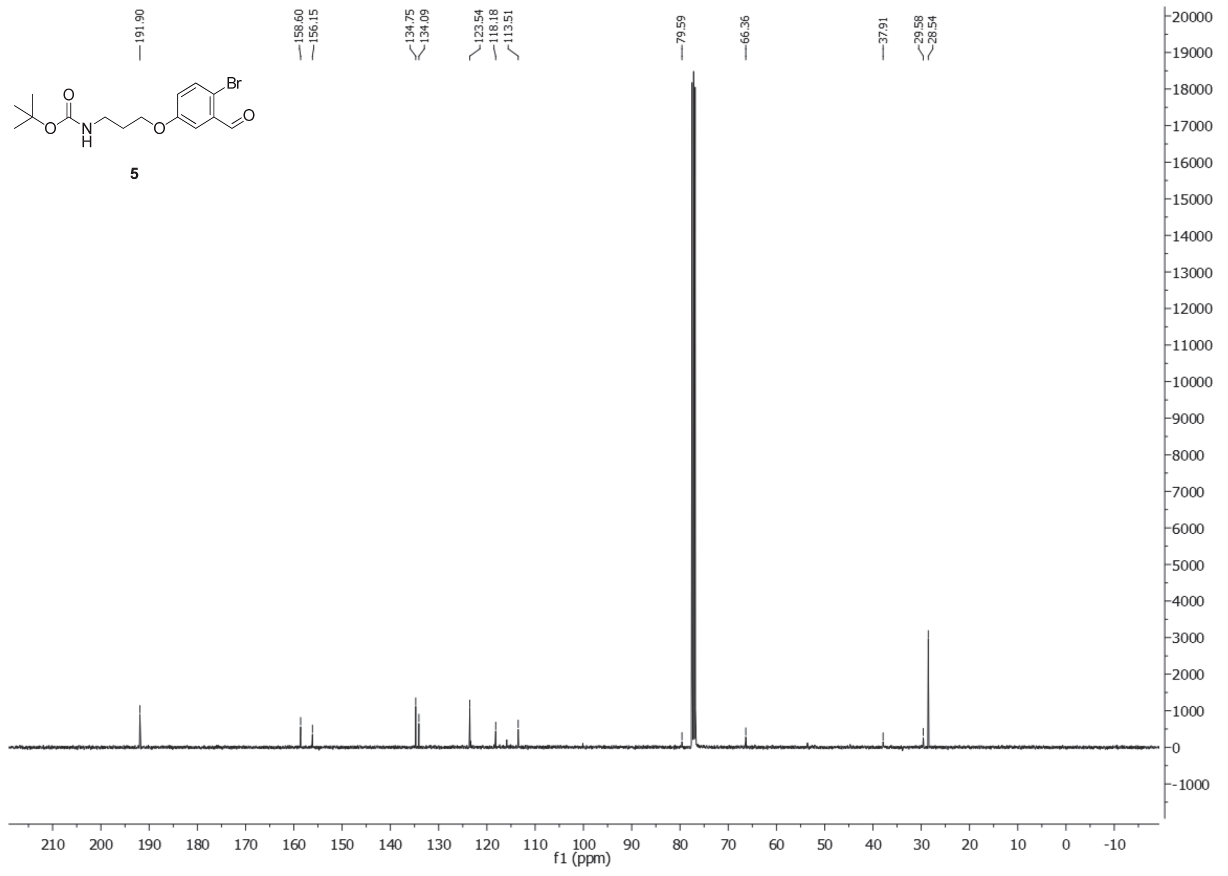
191.90
158.60
156.15
134.75
134.09
123.54
118.18
113.51
79.59
66.36
37.91
29.58
28.54
Br
O
O
N
H
O
O
5
f1 (ppm)

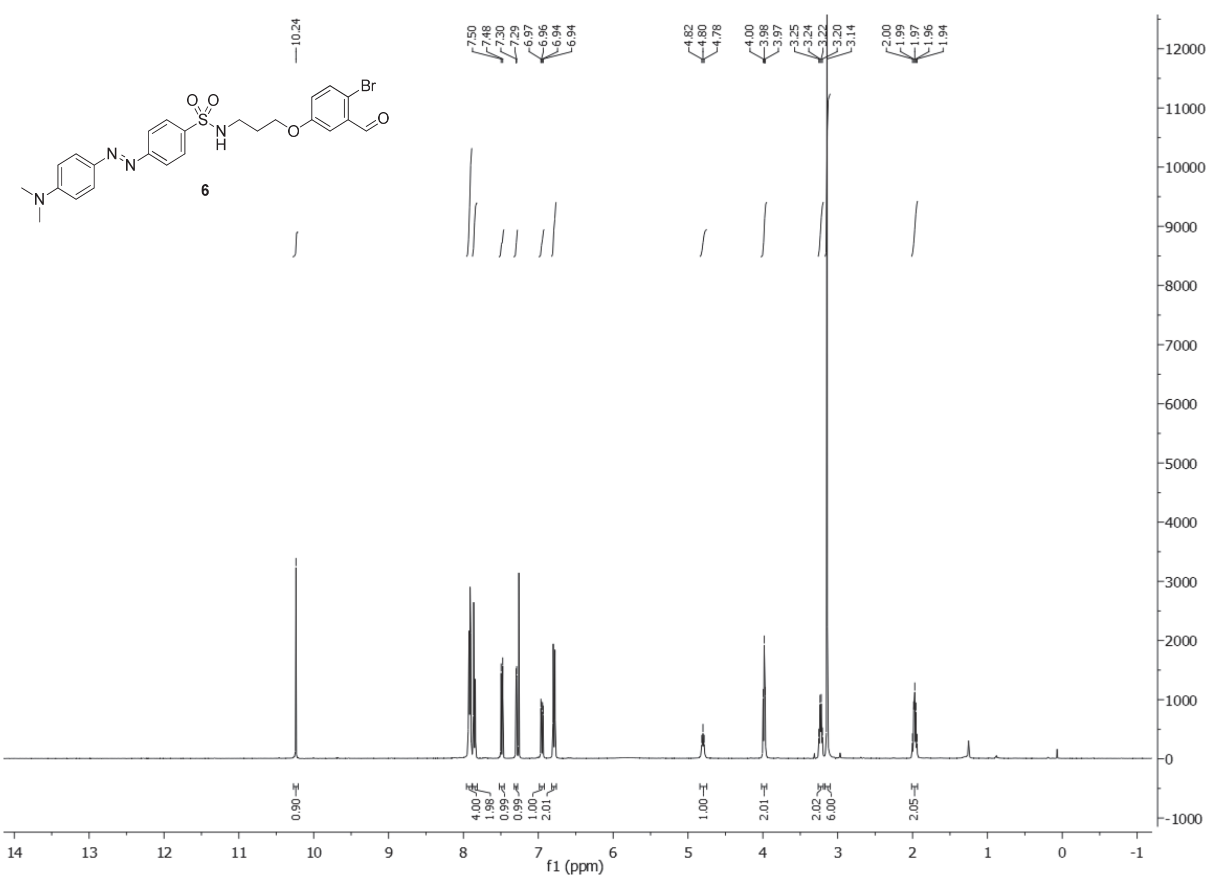

10.24
7.50 7.48 7.30 7.29 6.97 6.96 6.94 6.94
4.82 4.80 4.78
4.00 3.98 3.97
3.25 3.24 3.22 3.20 3.14
2.00 1.99 1.97 1.96 1.94
Br
O
S
O O
N
H
N
N
N
6
0.90
4.00 1.98 0.99 0.99 1.00 2.01
1.00
2.01
2.02 6.00
2.05
f1 (ppm)

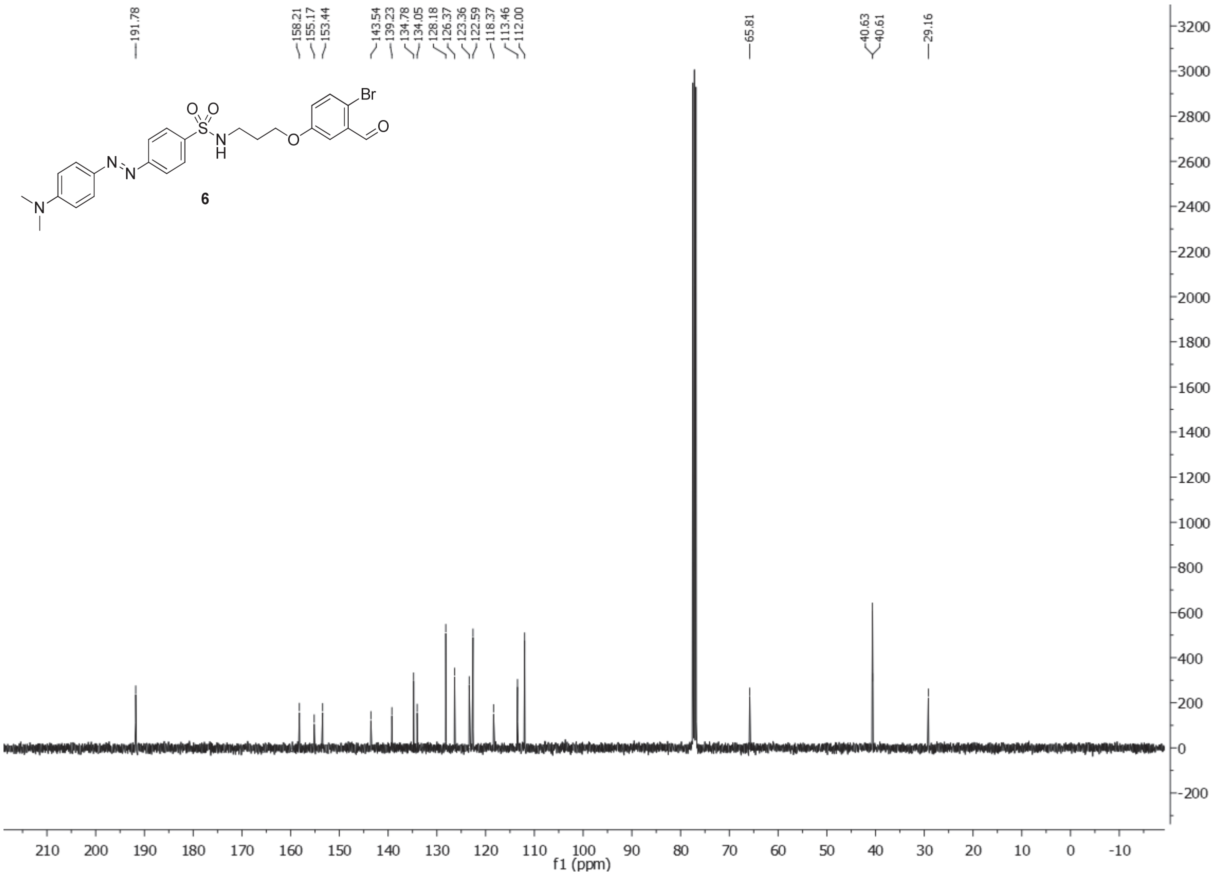

191.78
158.21 155.17 153.44
143.54 139.23 134.78 134.05 128.18 126.37 123.36 122.59 118.37 113.46 112.00
65.81
40.63 40.61
29.16
Br
O
S
O O
N
H
N
N
N
6
f1 (ppm)

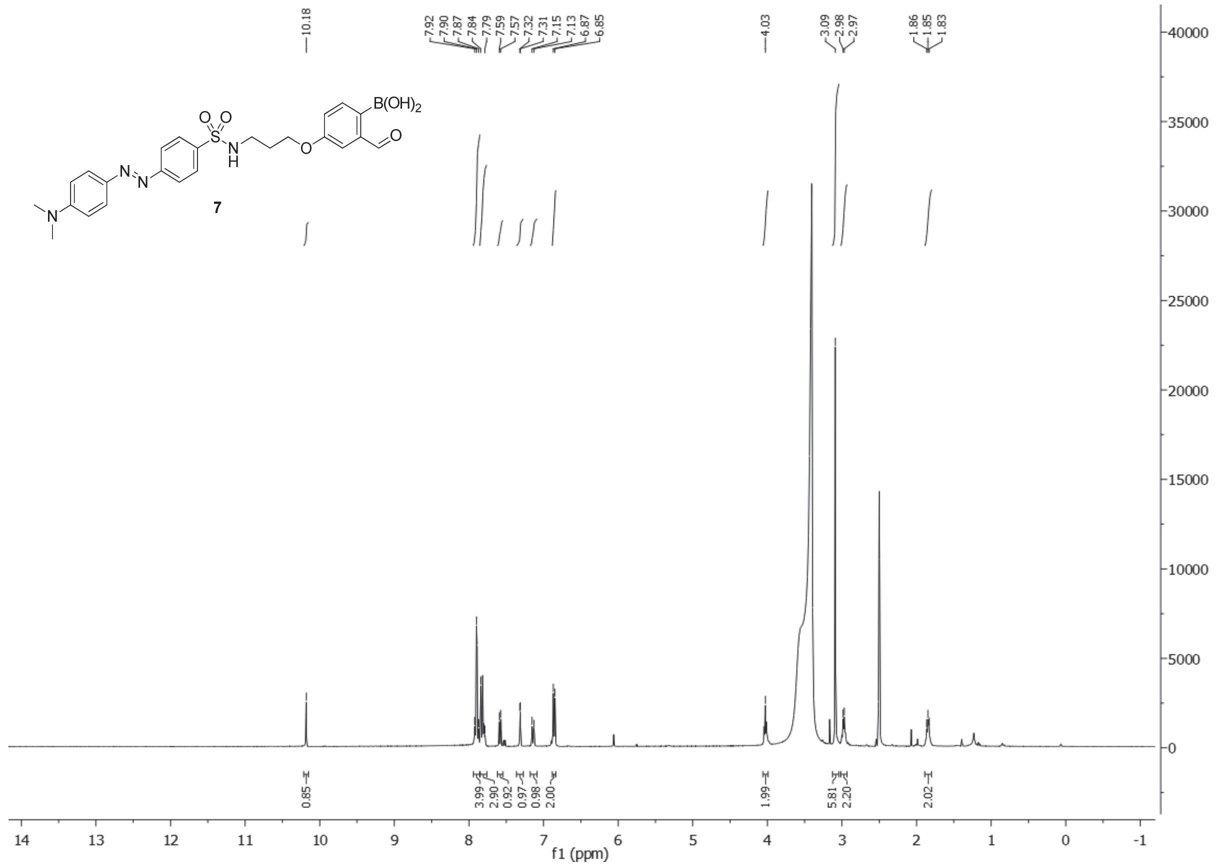
10.18
7.92
7.90
7.87
7.84
7.79
7.59
7.57
7.32
7.31
7.15
7.13
6.87
6.85
4.03
3.09
2.98
2.97
1.86
1.85
1.83
B(OH)2
7
0.85
3.99
2.90
0.92
0.97
0.98
2.00
1.99
5.81
2.20
2.02
f1 (ppm)

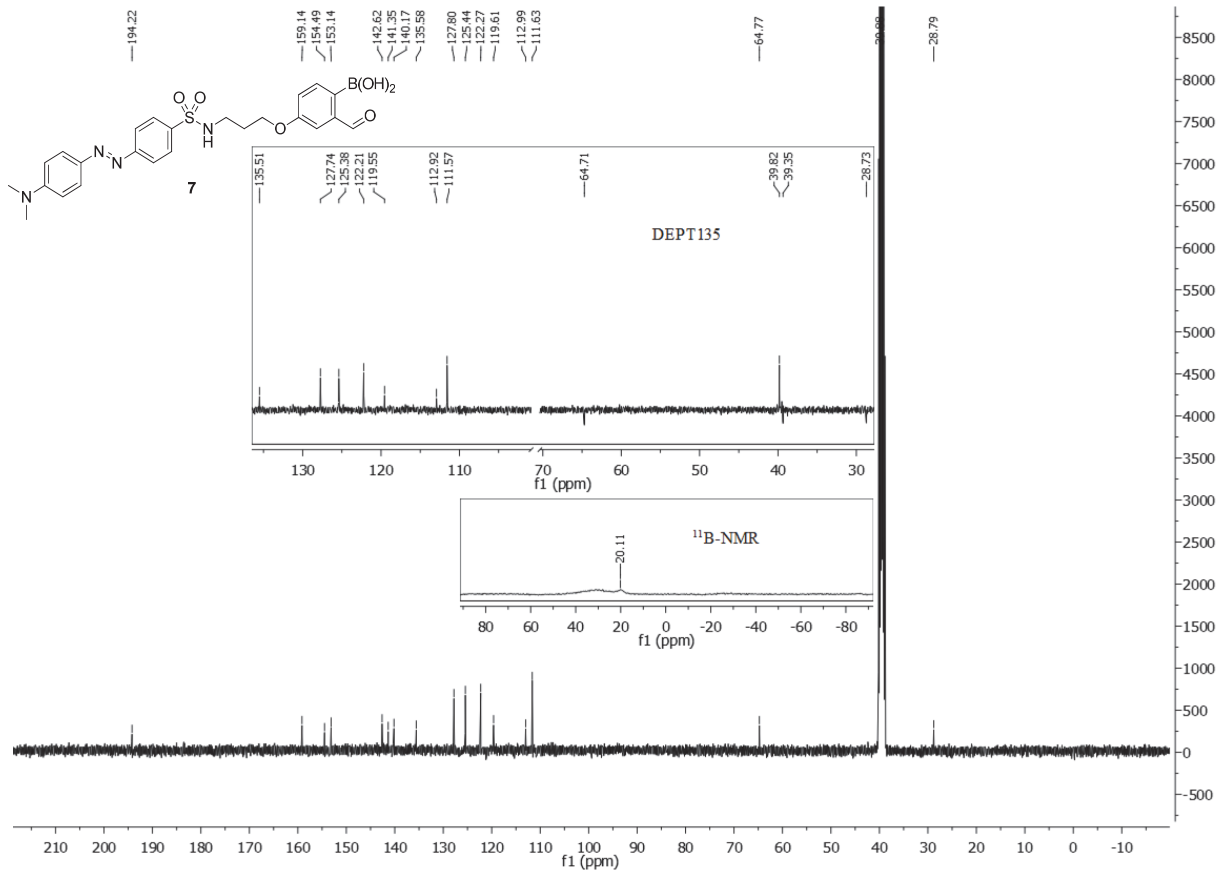
194.22
159.14
154.49
153.14
142.62
141.35
140.17
135.58
127.80
125.44
122.27
119.61
112.99
111.63
64.77
28.79
B(OH)2
7
135.51
127.74
125.38
122.21
119.55
112.92
111.57
64.71
39.82
39.35
28.73
DEPT135
f1 (ppm)
20.11
11B-NMR
f1 (ppm)
f1 (ppm)

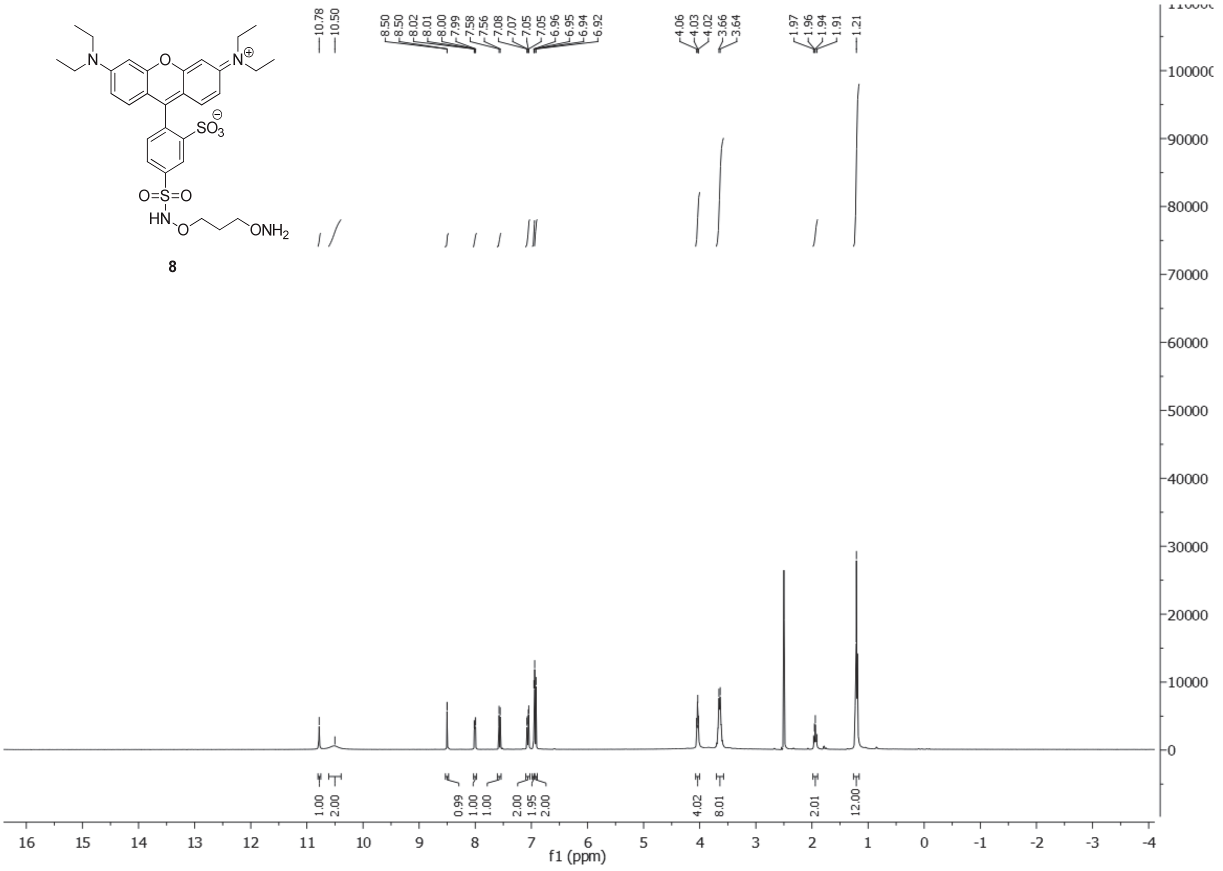
8
f1 (ppm)

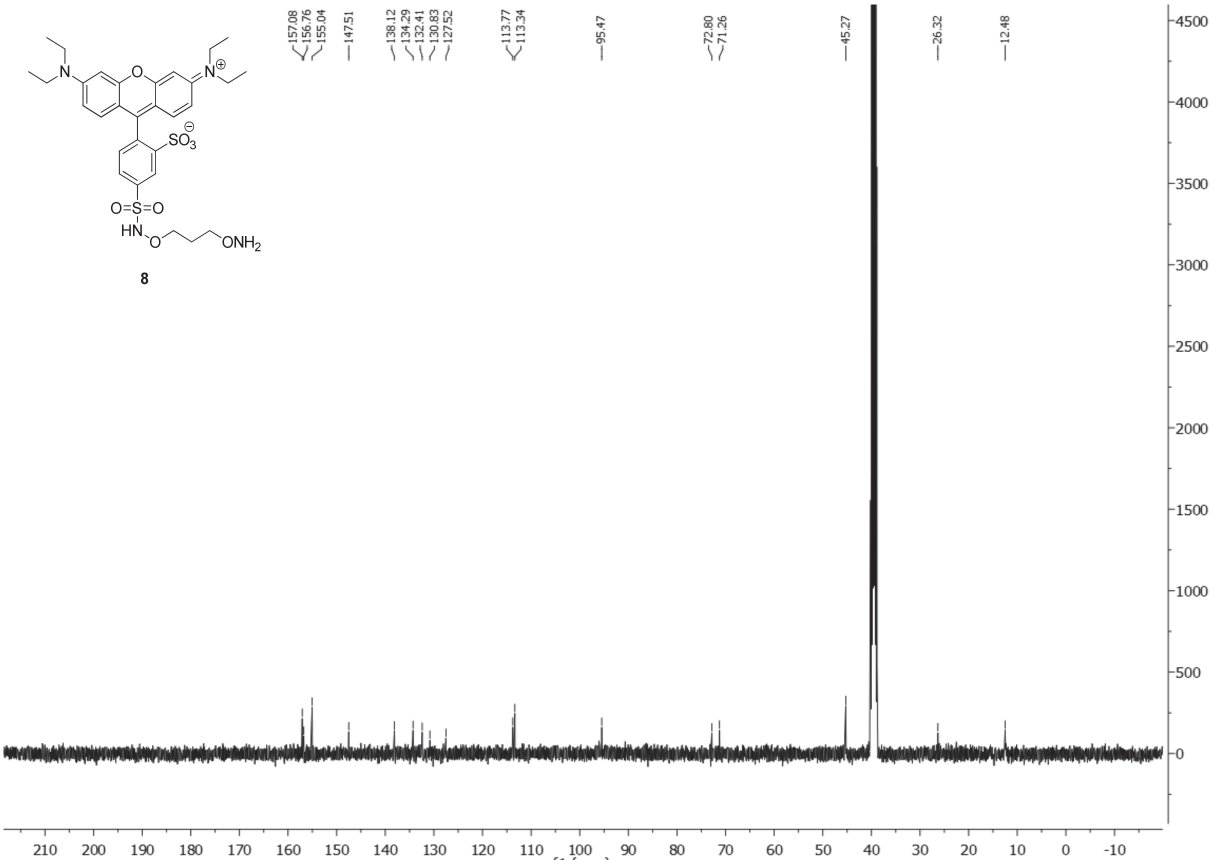
8
f1 (ppm)

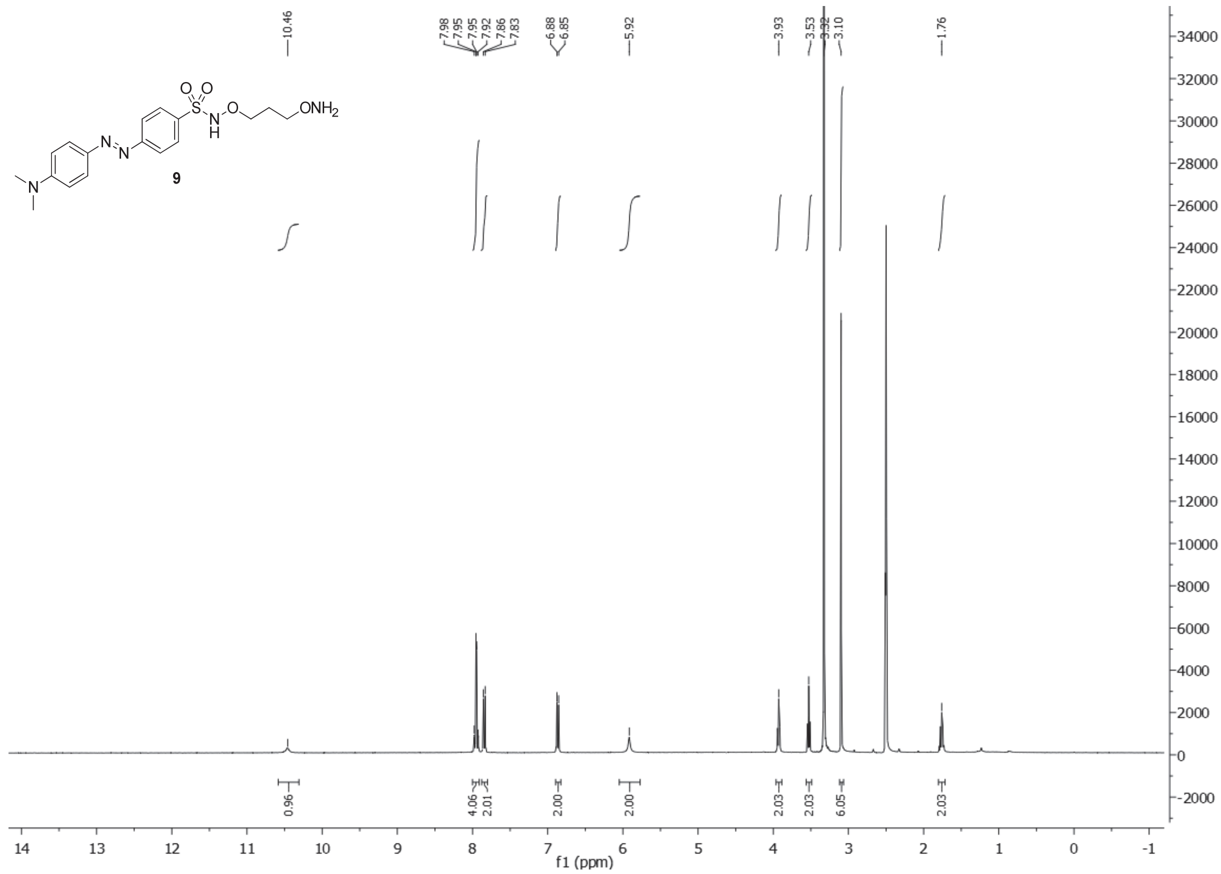

10.46
7.98
7.95
7.95
7.92
7.86
7.83
6.88
6.85
5.92
3.93
3.53
3.32
3.10
1.76
ONH2
9
0.96
4.06
2.01
2.00
2.00
2.03
2.03
6.05
2.03
f1 (ppm)

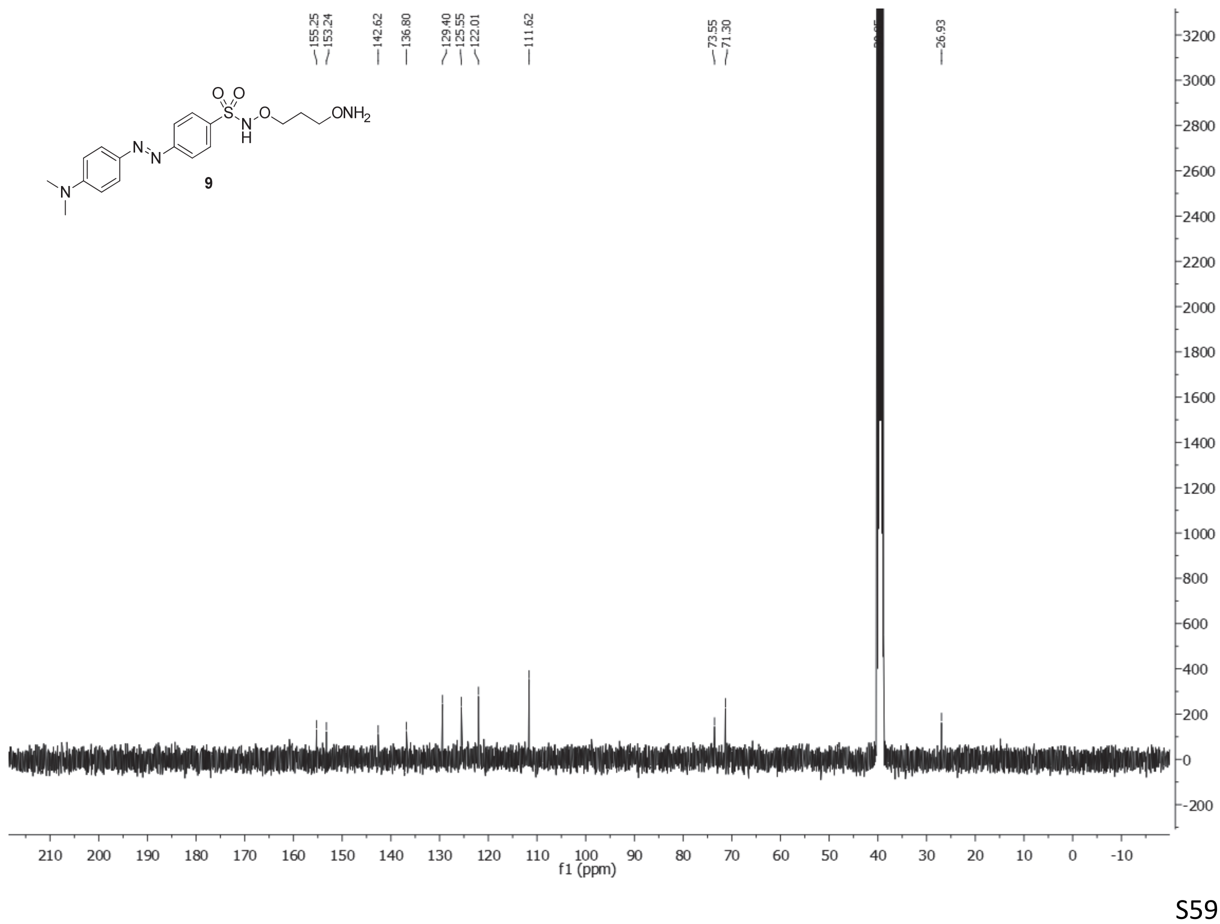

155.25
153.24
142.62
136.80
129.40
125.55
122.01
111.62
73.55
71.30
26.93
ONH2
9
f1 (ppm)

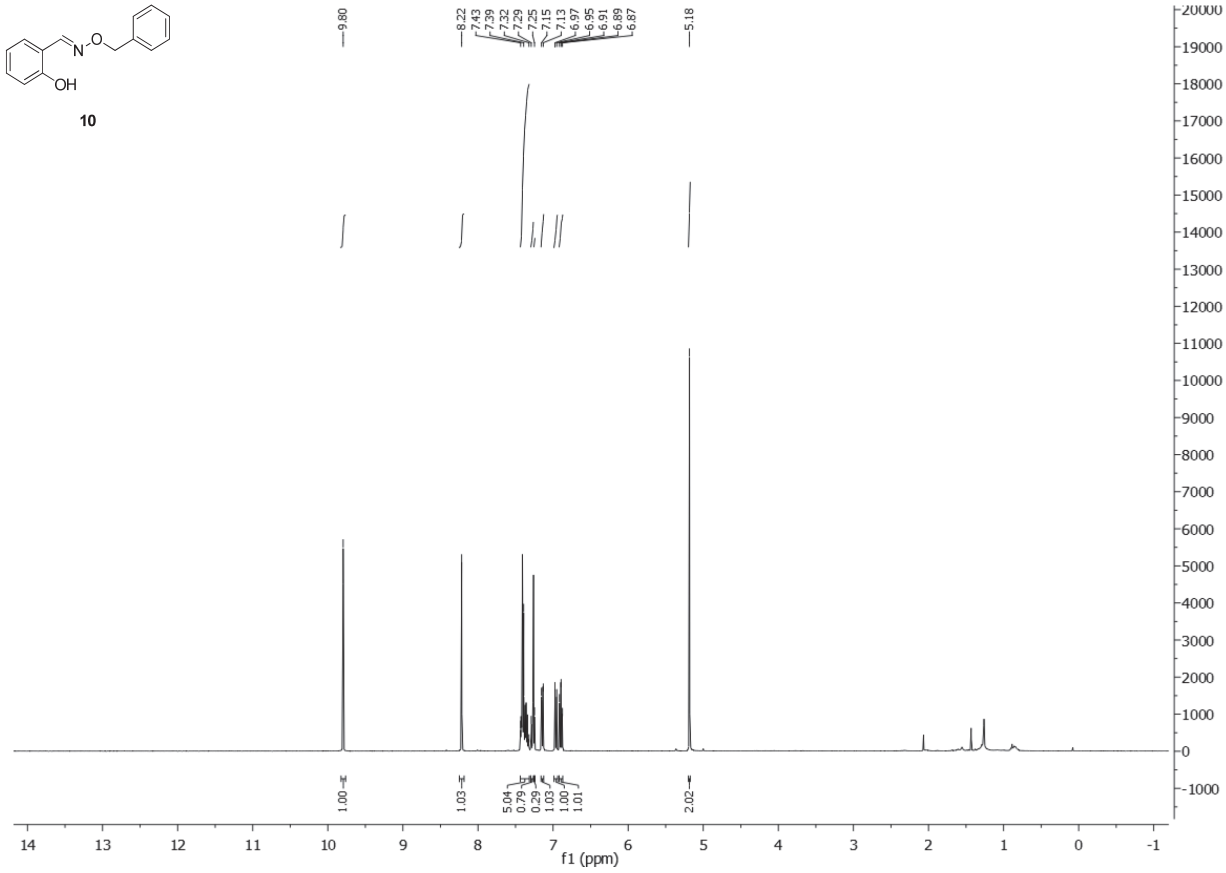
10
9.80
8.22
7.43
7.39
7.32
7.29
7.25
7.15
7.13
6.97
6.95
6.91
6.89
6.87
5.18
1.00
1.03
5.04
0.79
0.29
1.03
1.00
1.01
2.02
f1 (ppm)

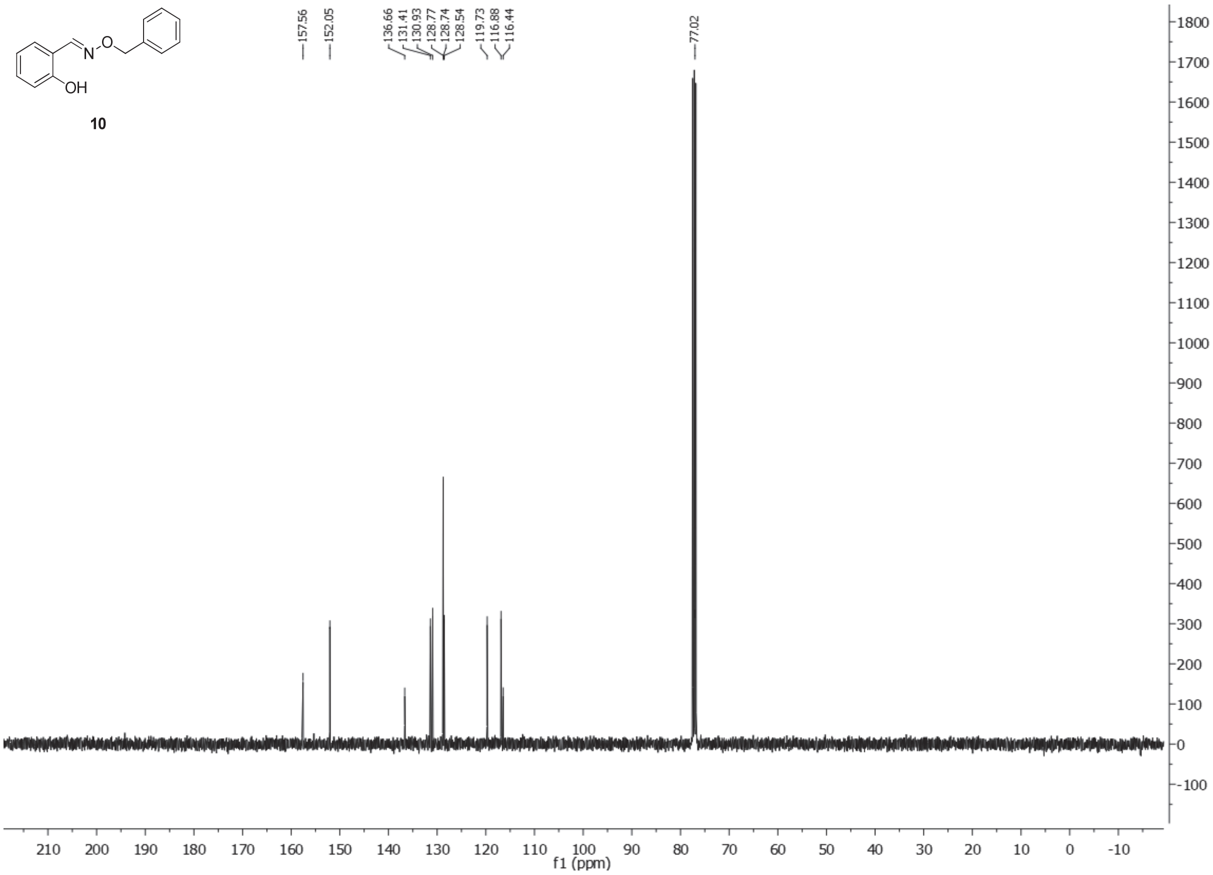
10
157.56
152.05
136.66
131.41
130.93
128.77
128.74
128.54
119.73
116.88
116.44
77.02
f1 (ppm)

8. References

[1] M. Szabo, C. Klein Herenbrink, A. Christopoulos, J. R. Lane, B. Capuano, *J. Med. Chem.*, 2014, **57**, 4924-4939.

[2] L. Nieto, A. Mascaraque, F. Miller, F. Glacial, C. R. Martínez, M. Kaiser, R. Brun, C. Dardonville, *J. Med. Chem*., 2011, **54**, 485-494.

[3] P. Schmidt, L. N. Zhou, K. Tishinov, K. Zimmermann, D. Gillingham, *Angew. Chem. Int. Ed.*, 2014, **53**, 10928-10931.

[4] E. Sella, D. Shabat, *Org. Biomol. Chem.*, 2013, **11**, 5074-5078.
